# Analysis and Optimal Velocity Control of a Stochastic Convective Cahn–Hilliard Equation

**DOI:** 10.1007/s00332-021-09702-8

**Published:** 2021-04-03

**Authors:** Luca Scarpa

**Affiliations:** 1 Faculty of Mathematics, University of Vienna, Oskar-Morgenstern-Platz 1, 1090 Vienna, Austria; 2 Department of Mathematics, Politecnico di Milano, Via E. Bonardi 9, 20133 Milan, Italy

**Keywords:** Stochastic Cahn–Hilliard equation, Convection, Well-posedness, Optimal velocity control, Optimality conditions, 35K25, 35R60, 60H15, 80A22

## Abstract

A Cahn–Hilliard equation with stochastic multiplicative noise and a random convection term is considered. The model describes isothermal phase-separation occurring in a moving fluid, and accounts for the randomness appearing at the microscopic level both in the phase-separation itself and in the flow-inducing process. The call for a random component in the convection term stems naturally from applications, as the fluid’s stirring procedure is usually caused by mechanical or magnetic devices. Well-posedness of the state system is addressed, and optimisation of a standard tracking type cost with respect to the velocity control is then studied. Existence of optimal controls is proved, and the Gâteaux–Fréchet differentiability of the control-to-state map is shown. Lastly, the corresponding adjoint backward problem is analysed, and the first-order necessary conditions for optimality are derived in terms of a variational inequality involving the intrinsic adjoint variables.

## Introduction

The aim of this paper is to analyse the stochastic Cahn–Hilliard equation with convection

1.1 $$\begin{aligned} {\mathrm d}\varphi - \Delta \mu \,{\mathrm d}t + {{\mathbf {u}}}\cdot \nabla \varphi \,{\mathrm d}t = B(\varphi )\,{\mathrm d}W \qquad&\text {in } (0,T)\times {\mathcal {O}}=:Q\,, \end{aligned}$$

1.2 $$\begin{aligned} \mu = -\Delta \varphi + \Psi '(\varphi ) \qquad&\text {in } (0,T)\times {\mathcal {O}}\,, \end{aligned}$$

1.3 $$\begin{aligned} \mathbf{n}\cdot \nabla \varphi = \mathbf{n}\cdot \nabla \mu = 0 \qquad&\text {in } (0,T)\times \partial {\mathcal {O}}\,, \end{aligned}$$

1.4 $$\begin{aligned} \varphi (0)=\varphi _0 \qquad&\text {in } {\mathcal {O}}\,, \end{aligned}$$

where ${\mathcal {O}}$ is a smooth bounded domain in ${\mathbb {R}}^d$, $d=2,3$, $T>0$ is a fixed final time, and $\mathbf{n}$ denotes the normal outward unit vector on $\partial {\mathcal {O}}$. The system (1.1)-(1.4) models isothermal phase-separation occurring in a moving fluid occupying the space region ${\mathcal {O}}$ during the time interval [0, *T*]. The order parameter, or phase-variable, $\varphi$ represents the relative concentration between the pure phases, the variable $\mu$ represents the chemical potential of the system, and the nonlinearity $\Psi :{\mathbb {R}}\rightarrow {\mathbb {R}}$ is a double-well potential with two global minima. The term ${{\mathbf {u}}}$ is an external random velocity field acting on the system, modelling possible stirring and mixing processes of the fluid which may affect phase-separation itself. The stochastic forcing describing the thermal fluctuations affecting phase-separation is modelled by means of a cylindrical Wiener process *W* on a given probability space and a *W*-integrable coefficient *B*, possibly depending on the phase variable itself, which calibrates the intensity of the noise.

The Cahn–Hilliard equation is a classical model employed in phase-separation, and has nowadays numerous applications to physics, biology, and engineering. Its introduction dates back to the pioneering work by Cahn and Hilliard (1958), where it was proposed, in the deterministic version, to adequately describe spinodal decomposition in binary metallic alloys. In the last decades, the model has been extensively refined in several directions. For example, the description of possible viscous behaviours has been originally presented in Elliott and Stuart (1996), Elliott and Songmu (1986), Novick-Cohen (1988), and then generalised in Gurtin (1996). The presence of a further evolution close to boundary due to the interaction with the hard walls has been accounted for by proposing several choices of dynamic boundary conditions, for which we refer to (Fischer et al. 1997; Kenzler et al. 2001; Gal 2012).

The deterministic Cahn–Hilliard equation has been proven to be extremely effective in describing phase-separation phenomena. Nevertheless, it presents some drawbacks. Indeed, the phase-separation process inevitably presents some disruptions, acting at a microscopic level. These are due to unpredictable movements at the atomistic level, which may be caused, for example, by temperature oscillations, magnetic effects, or configurational interactions. As such, the classical Cahn–Hilliard system is unable to capture the erratic nature of the separation process. The most natural way to overcome this problem is to switch to a random setting instead, by introducing a suitable noise term in the equation that could effectively describe the unpredictability of the phenomenon at a small scale. This was proposed by Cook (1970) for Wiener-type noises and gave rise to the well-known Cahn–Hilliard–Cook stochastic model for phase-separation. The stochastic version of the model was then confirmed multiple times (Binder 1981; Pego 1989) to be the only one that can genuinely describe phase-separation in alloys. Since then, the random version of the equation has been increasingly studied, both in the physics literature (Rogers et al. 1988; Elder et al. 1988; Grant et al. 1985; Langer et al. 1975; Milchev et al. 1988) and in the direction of model validation and numerical simulations (Blömker et al. 2001, 2008, 2016; Hawick 2010; Hawick and Playne 2010; Hawick 2008; Lee et al. 2014).

The classical Cahn–Hilliard equation is the gradient flow associated with the free energy functional

$$\begin{aligned} \varphi \mapsto \frac{1}{2}\int _{\mathcal {O}}|\nabla \varphi |^2 + \int _{\mathcal {O}}\Psi (\varphi )\,, \end{aligned}$$

with respect to the metric of $H^1({\mathcal {O}})^*$. The gradient term penalises the oscillation of the order parameter, while the double-well potential models the tendency of each phase to concentrate. The form of the chemical potential in (1.2) appears then naturally from the differentiation of the free energy. Typical examples of $\Psi$ are given by

1.5 $$\begin{aligned} \Psi _{log}(r):= & {} \frac{\theta }{2}\left( (1+r)\ln (1+r) + (1-r)\ln (1-r)\right) \frac{\theta _0}{2}r^2\,,\nonumber \\&- \quad r\in (-1,1)\,, \qquad 0<\theta <\theta _0\,, \end{aligned}$$

and

1.6 $$\begin{aligned} \Psi _{pol}(r):=\frac{1}{4}(r^2-1)^2\,, \quad r\in {\mathbb {R}}. \end{aligned}$$

Although (1.5) is the most relevant choice in terms of thermodynamical consistency, its singular behaviour in $\pm 1$ could be hard to tackle from the mathematical viewpoint, and in several models the polynomial approximation (1.6) is often employed.

The velocity field ${{\mathbf {u}}}$ models the transport effects due to convection terms acting on the system. In our analysis, this will be a prescribed external forcing field which will play the role of velocity control in a typical optimisation problem. Optimisation involving phase-separating fluids where the velocity is the control arises naturally in applications. For example, this is the case of block solidification of silicon crystals in photovoltaic applications. Here, the flow of the fluid acts as a control to optimise the distribution of certain impurities, at the atomistic level, in a process of solidification of silicon melt. For more details about the applications of optimal velocity control problem in phase-separating fluids, we refer to (Kudla et al. 2013; Rocca and Sprekels 2015). In practice, the motion of the fluid can be achieved in several ways: as pointed out in Colli et al. (2018a), Rocca and Sprekels (2015), the most common choices consist in employing either mechanical stirring devices or ultrasound emitters directly into the container. Another possibility is to prescribe a velocity on the fluid by means of magnetic fields: this is widely employed, for example, in the case of molten metals (Kudla et al. 2013) or bulk semiconductor crystals. Nevertheless, it is worthwhile noting that in all these scenarios, the velocity field is usually obtained in an *indirect* way, meaning that the motion of the fluid is achieved only as a consequence of more *direct* controls, such as mechanical devices or magnetic effects. This being noticed, it is clear then that the external prescription of a given velocity is strongly affected by microscopic noises, which may be caused, depending on the type of motion-inducing devices, by configurational or electromagnetic disturbances occurring in the flow-creating process. Also, the effective induction of the flow is strongly affected by the imprecision of the above-mentioned devices.

From the modelling point of view, this strongly calls for the introduction of a further source of randomness in the velocity field ${{\mathbf {u}}}$ and for abandoning the classical deterministic setting of the problem. Let us stress that the random component of the velocity field prescinds from the stochastic nature of the noise in equation (1.1): while the Wiener process *W* models microscopic turbulences occurring in phase-separation, the random nature of ${{\mathbf {u}}}$ takes into account the imprecision of the flow-inducing mechanisms. For example, in typical situations ${{\mathbf {u}}}$ would satisfy a further stochastic equation involving a further Wiener process, independent of *W*. Clearly, this extra equation would specifically depend on the model in consideration: here, in order to make the treatment as general and light as possible, we only require *u* to be a stochastic process. Let us point out that this choice implies that the microscopic fluctuations in ${{\mathbf {u}}}$ coming from a possible further noise are not taken into account explicitly here. Indeed, the box constraint for the controls (see Sect. 2 below) only requires some general measurability and integrability conditions on ${{\mathbf {u}}}$, and does not prescribe any specific requirement on the microscopic fluctuations of ${{\mathbf {u}}}$. To fix the ideas, the reader can naturally think about focusing only on macroscopic controls, e.g. controls which are $C^1$ in time and $W^{1,p}$ in space, and neglecting thus the microscopic turbulence in ${{\mathbf {u}}}$. Here, since the methodology can be directly adapted to more general controls, we preferred to consider a broader class of admissible controls, for sake of mathematical generality.

The importance of allowing the control variable to be random is crucial when dealing with a controlled stochastic equation (see, for example, Yong and Zhou 1999). Indeed, bearing in mind the typical perspective of Monte Carlo simulations, restricting to deterministic controls would mean to choose *a priori* a control which is independent of the possible outcomes of the evolution according to the prescribed underlying probability space. By contrast, stochastic controls ensure more freedom from the point of view of the controller, as they allow to adapt the control to the random outcomes of the phenomenon itself. With this in mind, in our analysis ${{\mathbf {u}}}$ will be a prescribed stochastic process satisfying some natural box-constraints, possibly taking into account the random imprecision of the velocity-inducing devices. The model that we study presents then two main sources of randomness: the first one is given by the Wiener noise in equation (1.1), taking into account the microscopic turbulence affecting phase-separation, and the second one is the stochastic component of the convection term, modelling the imprecision of the stirring procedure. Hence, one can think the two random forcings as acting on two separate levels: a microscopic scale described by *W*, and a different uncorrelated scale rendered by ${{\mathbf {u}}}$.

The mathematical literature dealing with the Cahn–Hilliard equation is extremely developed. In the deterministic case, attention has been widely devoted to the study of well-posedness, regularity, long-time behaviour of solutions, and asymptotics. Due to the considerable size of the literature, we prefer to quote the detailed overview by Miranville (2019) and the references therein for completeness. Let us only point out the contributions (Colli et al. 2014; Cherfils et al. 2011; Gilardi et al. 2009) dealing with well-posedness and (Colli et al. 2015a, b, 2016; Hintermüller and Wegner 2012) in the direction of distributed and boundary control problems. Possible relaxations and asymptotics of the Cahn–Hilliard equation have been recently studied in Bonetti et al. (2017, 2018, 2020), Colli and Scarpa (2016), Scarpa (2019a) also with nonlinear viscosity terms.

In the stochastic case, the original contribution dealing with Cahn–Hilliard equation is (Da Prato and Debussche 1996), on the existence of mild solutions in the case of polynomial potentials. Further studies have been then carried out in the works (Cornalba 2016; Elezović and Mikelić 1991) again in the polynomial setting, and in Scarpa (2018, 2020) in the case of more general potentials in variational framework. The stochastic Cahn–Hilliard equation with logarithmic potential has been studied in Debussche and Zambotti (2007), Debussche and Goudenège (2011); Goudenège (2009) in relation to reflection measures, and in Scarpa (2019) in the case of degenerate mobility. In the context of phase-field modelling with stochastic forcing, it is worthwhile mentioning the contributions (Antonopoulou et al. 2016; Feireisl and Petcu 2019a, b), as well as (Bauzet et al. 2017; Bertacco 2020; Orrieri and Scarpa 2019) on the stochastic Allen–Cahn equation. In the direction of optimal control, we point out (Scarpa 2019b) dealing with a distributed optimal control problem of the stochastic Cahn–Hilliard equation, and the recent work (Orrieri et al. 2020) on a stochastic phase-field model for tumour growth.

Concerning specifically the Cahn–Hilliard equation with convection, in the deterministic case well-posedness has been studied in Colli et al. (2018a) under general choices of dynamic boundary conditions, in Porta and Grasselli (2015) in a local version with reaction terms, while some related optimal velocity control problems have been analysed in Colli et al. (2018b, 2019), Rocca and Sprekels (2015), Zhao and Liu (2013), and Zhao and Liu (2014). Also, the relationship between the behaviour of the convection term and phase-separation has been analysed in the recent work (Feng et al. 2020): here, the authors show that if the velocity field is sufficiently mixing, then no phase-separation occurs, and the solutions of the respective advective Cahn–Hilliard equation converge exponentially to a homogenous mixed state instead. This may have important connections to related optimal control problems with a target distribution at a final time: in particular, the above-mentioned result makes the optimisation problem meaningful also when the final target state is not necessarily separated, but is a homogenous mixed state. Also, it points out how powerful the action of the convection term is on the phase-separation, and motivates the study of phase-optimisation problems where the control is the velocity itself. The convective Cahn–Hilliard equation has also been considered in coupled systems, with a further equation equation for the velocity field: it is the case, for example, of Cahn–Hilliard–Navier–Stokes systems, studied in Abels (2009), and Frigeri et al. (2019, 2020, 2016). By contrast, despite its strong relevance in application to stochastic optimal velocity control, the convective Cahn–Hilliard has not been analysed yet. The only results available in the stochastic setting deal with coupled systems, for example in the context of stochastic Cahn–Hilliard–Navier–Stokes models (Deugoué and Medjo 2018b, a; Medjo 2017). This paper constitutes a first contribution to optimal velocity control for the stochastic convective Cahn–Hilliard equation.

The literature on stochastic optimal control is also quite extensive: for a general overview we refer to the monograph (Yong and Zhou 1999). Stochastic optimal control is also studied in Fuhrman et al. (2012, 2013, 2018), Fuhrman and Orrieri (2016), Guatteri et al. (2017) in the context of the heat equation and reaction-diffusion systems. For completeness, we refer also to the works (Du and Meng 2013; Lü and Zhang 2014) concerning the stochastic maximal principle. Relaxation of the optimality conditions has been addressed in Brzeźniak and Serrano (2013) and Barbu et al. (2018) for dissipative SDPEs and the Schrödinger equation, respectively. Deterministic optimal control problems of stochastic reaction–diffusion equations have been analysed in Stannat and Wessels (2019).

Let us describe now the main points that will be addressed in this work. First of all, we concentrate on the well-posedness of the state-system (1.1)–(1.4), where the control ${{\mathbf {u}}}$ is arbitrary but fixed. Using a Yosida approximation on the nonlinearity and a time-regularisation on the velocity field, we show existence-uniqueness of solutions by means of variational techniques and stochastic compactness arguments. Thanks to monotone analysis tools, we are able to cover very general potentials, not necessarily of polynomial growth. Also, we prove continuous dependence of the variables with respect to the control, and this allows to define a suitable control-to-state map $S:{{\mathbf {u}}}\mapsto (\varphi ,\mu )$. Secondly, we focus on the optimisation problem, which consists in minimising a tracking-type cost functional in the form:

$$\begin{aligned} J(\varphi ,{{\mathbf {u}}}):= \frac{\alpha _1}{2}{{\mathbb {E}}}\int _Q|\varphi -\varphi _Q|^2 +\frac{\alpha _2}{2}{{\mathbb {E}}}\int _{\mathcal {O}}|\varphi (T) - \varphi _T|^2 +\frac{\alpha _3}{2}{{\mathbb {E}}}\int _Q|{{\mathbf {u}}}|^2 \end{aligned}$$

subject to the state-system (1.1)–(1.4) and the constraint that ${{\mathbf {u}}}$ is an admissible control, meaning that ${{\mathbf {u}}}\in {\mathcal {U}}_{ad}$ with ${\mathcal {U}}_{ad}$ being a suitable bounded, closed subset of the space *p*-integrable progressively measurable process with values in $L^3({\mathcal {O}})^d$. Here, $\varphi _Q$ and $\varphi _T$ represent some running and final targets, while $\alpha _1, \alpha _2, \alpha _3$ are nonnegative weights.

Cost functionals in this form arise very naturally from applications. Roughly speaking, the optimisation problem amounts to identify the optimal way of stirring and mixing the fluid in such a way that the state variable $\varphi$ is as close as possible to the running target $\varphi _Q$ during the evolution and to the final target $\varphi _T$ at the end of the evolution, without wasting too much energy in inducing the flow ${{\mathbf {u}}}$. As we have anticipated above, a typical example that we have in mind appears in the solidification process of silicon crystals in the context of industrial photovoltaic applications (Kudla et al. 2013; Rocca and Sprekels 2015). Here, a certain mixture of impurities needs to be moved by convection from within the silicon melt to its boundary, in order to refine the quality of the final silicon block. The flow ${{\mathbf {u}}}$ of the fluid behaves then as a control on the silicon melt in order to make the relative distribution of impurities $\varphi$ be close enough to some prescribed targets. In particular, the final target distribution $\varphi _T$ of impurities can be seen here as concentrated on the boundary and diluted in the interior. Analogous applications arise more generally in optimal distribution problems of melting materials: the local distribution of some substance contained in the separating fluid is optimised close to some desired targets by inducing a flow in the material itself.

The starting point in the analysis consists in addressing existence of optimal controls. This is one of the main differences with respect to the deterministic optimal control problem. Indeed, in the deterministic setting existence of optimal controls follows with no particular effort from the direct method of calculus of variations, since one is able to obtain enough compactness from the well-posedness of the state system and the boundedness of the set of admissible controls. By contrast, in the stochastic case these uniform estimates on the minimising sequence of controls do not ensure enough compactness in probability, due to the stochastic nature of the problem itself. Also, classical stochastic tools that are usually employed to bypass this problem, such as the well-known criterion à la Gyöngy–Krylov, do not work here: this is due to the non-uniqueness of optimal controls, which is caused by the highly nonlinear nature of the minimisation problem. To overcome this issue, we propose instead a relaxed notion of optimality, which may be considered as optimality in law, i.e. requiring that the stochastic basis and the Wiener process are part of the definition of optimal control themselves. This technique mimics the definition of probabilistically weak solution for stochastic evolution equations, and has been employed in other settings such as (Barbu et al. 2018; Orrieri et al. 2020). In this framework, we prove existence of relaxed optimal controls, and we show that when one restricts the attention only to deterministic controls, then it is possible to get existence in the classical (probabilistically strong) sense.

We move then to the study of the differentiability properties of the control-to-state map *S*. More specifically, we prove that *S* is Gâteaux and Fréchet differentiable between suitable Banach spaces. This is done by showing well-posedness of the so-called linearised system, obtained from (1.1)–(1.4) formally differentiating with respect to ${{\mathbf {u}}}$, and by carefully proving that the unique linearised solution actually coincides with the derivative of *S*. This will allow to explicitly characterise, thanks to the chain rule in Banach spaces, the derivative of the reduced cost functional $J\circ S$, so that the optimisation problem could be seen only in terms of the control ${{\mathbf {u}}}$. Consequently, it is possible to obtain a first rudimental version of necessary conditions for optimality, by imposing the classical first-order variational inequality $D(J\circ S)({{\mathbf {u}}})\ge 0$ on a given optimal control.

The last part of the paper aims at refining the first version of necessary conditions, by removing any explicit dependence on the linearised variables. This is done by introducing and studying a suitable adjoint problem, which is formally related to the dual problem of the linearised system. The adjoint problem consists of a backward-in-time stochastic partial differential equation, and its analysis is the most challenging point of the work. The first main difficulty is indeed the backward nature of the equation: although this is not a great limitation in deterministic problems, in the stochastic case it calls for the introduction of an extra variable, in order to preserve adaptability of the processes in play, and requires different analytical techniques such as martingale representation theorems. The second and most crucial difficulty depends instead on the nonlinear nature of the system. Indeed, the presence of the nonlinear term $\Psi ''(\varphi )$ and the dual structure of the equation prevent from obtaining uniform estimates directly on the adjoint system. Consequently, well-posedness cannot be obtained classically by tackling the adjoint problem straightaway, and a different idea is needed. In this regard, we use a duality method. We consider a more general version of the linearised system, where an arbitrary forcing term is added, and we show that this is well posed and the solutions depend continuously on the forcing term. Then, we prove that such system is in duality with the adjoint problem that we want to study, and this allows to recover by comparison some first uniform estimates on the adjoint variables. This tool is extremely powerful, as it allows to bound the adjoint variables without even working on the adjoint system itself: the main intuition behind this is that the linearised system is usually much simpler to study, and the duality between linearised-adjoint systems allows to “transfer” uniform bounds on the solutions from one problem to the other. Once these first crucial estimates are obtained, using classical techniques we are then able to prove well-posedness of the adjoint problem. Lastly, the duality relation is employed to refine the first-order conditions for optimality and to write them as a variational inequality only depending on the intrinsic adjoint variables.

The main novelty of the work is the presence of two sources of randomness in equation (1.1), accounting for noises both in the phase-separation process and in the flow-inducing procedure. As interesting as it may be from the applied point of view, certainly this novel framework does not come without effort on the mathematical side. Indeed, let us stress that the fact that ${{\mathbf {u}}}$ is assumed to be a stochastic process, and not a deterministic function, causes several non-trivial issues in estimating the solutions: this is due to a lack of satisfactory computational tools of Gronwall type in the genuinely pure stochastic case. Such difficulties are evident especially in the study of the forward problems, i.e. in the state system (1.1)–(1.4) and in the corresponding linearised system. Here, the idea is to argue instead combining carefully the Hölder inequality and several iterative patching arguments, in order to avoid applying the Gronwall lemma, which does not work. In the adjoint problem, the situation is slightly better: we will show that the backward nature of the equation allows indeed to use a very general and recent backward-in-time version of the stochastic Gronwall lemma (see Lemma 6.1).

We conclude by summarising here the structure of the paper. Section 2 contains the description of the setting of the work, the precise assumptions, and the main results that we prove. In Sect. 3, we prove well-posedness of the state-system, while Sect. 4 focuses on the existence of optimal controls. Then, in Sects. 5 and 6, we study the linearised system and the adjoint system, respectively. Finally, in Sect. 7, we prove the two versions of the first-order conditions for optimality.

## Setting and Assumptions

In this section, we specify the general setting, notation, and assumptions of the work. We then present the main results of the paper.

Let $(\Omega ,{\mathscr {F}}, ({\mathscr {F}}_t)_{t\in [0,T]}, {\mathbb {P}})$ be a filtered probability space satisfying the usual conditions, where $T>0$ is a fixed final time and *W* is a cylindrical Wiener process on a separable Hilbert space *K*. For convenience, let us fix now once and for all a complete orthonormal system $(e_j)_j$ of *K*. The progressive $\sigma$-algebra on $\Omega \times [0,T]$ is denoted by ${\mathscr {P}}$.

As far as notation is concerned, the dual of a given real Banach space *E* is denoted by $E^*$, and the duality pairing between $E^*$ and *E* is denoted by $\left\langle \cdot ,\cdot \right\rangle _{E^*,E}$. Weak convergence in *E* and weak$^*$ convergence in $E^*$ will be denoted by the respective symbols $\rightharpoonup$ and ${\mathop {\rightharpoonup }\limits ^{*}}$. Also, for all $q\in [1,+\infty ]$ we employ the usual symbols $L^q(\Omega ; E)$ and $L^q(0,T; E)$ for the spaces of *q*-Bochner integrable functions, and $C^0([0,T]; E)$ and $C^0_w([0,T]; E)$ for the spaces of strongly and weakly continuous functions from [0, *T*] to *E*, respectively. For spaces of stochastic processes, we use the notation $L^{q_1}_{\mathscr {P}}(\Omega ; L^{q_2}(0,T; E))$ to further specify that measurability is also intended with respect to the progressive $\sigma$-algebra ${\mathscr {P}}$. In the case that $q>1$ and *E* is separable, we explicitly set $L^q_w(\Omega ; L^\infty (0,T; E^*))$ as the dual space of $L^{\frac{q}{q-1}}(\Omega ; L^1(0,T; E))$, which we recall can be characterised (Edwards 1965, Thm. 8.20.3) as the space of weak*-measurable random variables $y:\Omega \rightarrow L^\infty (0,T; E^*)$ with finite *q*-moment in $\Omega$. Finally, if $E_1$ and $E_2$ are separable Hilbert spaces, we use the notation ${\mathscr {L}}^2(E_1,E_2)$ for the space of Hilbert–Schmidt operators from $E_1$ to $E_2$.

In the proofs, the symbol *c* is reserved to denote any generic positive constant, whose value depends on the structure of the problem and may be updated from line to line in the proofs.

Let ${\mathcal {O}}\subset {\mathbb {R}}^d$ ($d\ge 2$) be a smooth bounded domain. We use the classical notation $Q:=(0,T)\times {\mathcal {O}}$, $Q_t:=(0,t)\times {\mathcal {O}}$, and $Q_t^T:=(t,T)\times {\mathcal {O}}$ for every $t\in (0,T)$. The outward normal unit vector on the boundary $\partial {\mathcal {O}}$ is denoted by $\mathbf{n}$. We introduce the functional spaces

$$\begin{aligned}&H:=L^2({\mathcal {O}})\,, \qquad V_1:=H^1({\mathcal {O}})\,, \\&V_2:=\{v\in H^2({\mathcal {O}}):\;\mathbf{n}\cdot \nabla v = 0\quad \text {a.e. on } \partial {\mathcal {O}}\}\,, \qquad V_3:=V_2\cap H^3({\mathcal {O}})\,, \end{aligned}$$

endowed with their natural norms $\left\| \cdot \right\| _H$, $\left\| \cdot \right\| _{V_1}$, $\left\| \cdot \right\| _{V_2}$, and $\left\| \cdot \right\| _{V_3}$, respectively. We identify *H* to its dual, so that we have the continuous and dense inclusions

$$\begin{aligned} V_3\hookrightarrow V_2\hookrightarrow V_1\hookrightarrow H \hookrightarrow V_1^*. \end{aligned}$$

For all $y\in V_1^*$, we use the notation $y_{\mathcal {O}}:=\frac{1}{|{\mathcal {O}}|}\left\langle y,1\right\rangle$ for the spatial mean of *y*, and define the subspaces of zero-mean elements as

$$\begin{aligned} V_{1,0}^*:=\{y\in V_1^*:\;y_{\mathcal {O}}=0\}\,, \qquad H_0:=H\cap V_{1,0}^*\,, \qquad V_{1,0}:=V_1\cap H_0. \end{aligned}$$

Let us recall that the variational formulation of the Laplace operator with Neumann conditions

$$\begin{aligned} {\mathcal {L}}: V_1\rightarrow V_1^*\,, \qquad \left\langle {\mathcal {L}} y,\zeta \right\rangle :=\int _{\mathcal {O}}\nabla y\cdot \nabla \zeta \,,\quad y,\zeta \in V_1\,, \end{aligned}$$

is a well-defined linear operator, and its restriction to $V_{1,0}$ is an isomorphism onto the space $V_{1,0}^*$. Its inverse ${\mathcal {N}}: V_{1,0}^*\rightarrow V_{1,0}$ is the resolvent operator associated with the abstract elliptic problem on ${\mathcal {O}}$ with homogenous Neumann conditions, meaning that for all $y\in V_{1,0}^*$ the element $z:={\mathcal {N}}y\in V_{1,0}$ is the unique solution with null mean to

$$\begin{aligned} {\left\{ \begin{array}{ll} -\Delta z = y\quad &{}\text {in } {\mathcal {O}}\,,\\ \partial _\mathbf{n}z=0\quad &{}\text {in } \partial {\mathcal {O}}. \end{array}\right. } \end{aligned}$$

As a consequence of the Poincaré–Wirtinger inequality, it is immediate to check that

$$\begin{aligned} \zeta \mapsto \left\| \nabla {\mathcal {N}}(\zeta -\zeta _{\mathcal {O}})\right\| _H^2 + |\zeta _{\mathcal {O}}|^2\,,\qquad \zeta \in V_1^*\,, \end{aligned}$$

yields an equivalent norm on $V_1^*$. In particular, it follows the compactness inequality

2.1 $$\begin{aligned} \forall \,\varepsilon>0\,,\quad \exists \,c_\varepsilon >0:\quad \left\| y\right\| _H^2\le \varepsilon \left\| \nabla y\right\| _{H}^2 + c_\varepsilon \left\| \nabla {\mathcal {N}}y\right\| _H^2 \quad \forall \,y\in V_{1,0}. \end{aligned}$$

We introduce the space

$$\begin{aligned} U:=\left\{ \mathbf{u}\in L^3({\mathcal {O}}):\quad {\text {div}}{{\mathbf {u}}}=0\,,\quad {{\mathbf {u}}}\cdot \mathbf{n}=0 \text { a.e. on } \partial {\mathcal {O}}\right\} \,, \end{aligned}$$

where the divergence is intended in the sense of distributions on ${\mathcal {O}}$. The space of velocity controls $\mathbf{u}$ that we focus on will be

$$\begin{aligned} {\mathcal {U}}:=L^\infty _{\mathscr {P}}(\Omega ; L^p(0,T; U))\,, \qquad p\in (2,+\infty ). \end{aligned}$$

Let us note that this includes as a special case the choice of deterministic controls, which has also received a strong mathematical interest on its own: see, for instance, Stannat and Wessels (2019). Indeed, we can set

$$\begin{aligned} {\mathcal {U}}^{det}:=L^p(0,T; U)\subset {\mathcal {U}}. \end{aligned}$$

The following assumptions on the problem will be in force throughout the paper.

**A1:** $\Psi :{\mathbb {R}}\rightarrow {\mathbb {R}}$ is of class $C^2$, $\Psi '(0)=0$, and there exist $C_\Psi >0$ and $\gamma \in [1,2]$ such that
  $$\begin{aligned} \Psi ''(r)\ge -C_\Psi \qquad&\forall \,r\in {\mathbb {R}}\,,\\ |\Psi '(r)| + |\Psi ''(r)|^\gamma \le C_\Psi (1+ \Psi (r)) \qquad&\forall \,r\in {\mathbb {R}}. \end{aligned}$$
  Let us point out that the classical polynomial double-well potential $\Psi _{pol}$ satisfies these assumptions with $\gamma =2$. Nonetheless, by allowing also the smaller values $\gamma \in [1,2]$ we are able to include possibly more singular potential, such as the first-order exponentials. We set $\beta :r\mapsto \Psi '(r) + C_\Psi r$, $r\in {\mathbb {R}}$: then $\beta :{\mathbb {R}}\rightarrow {\mathbb {R}}$ is a $C^2$ nondecreasing function; hence, it can be identified with a maximal monotone (single-valued) graph in ${\mathbb {R}}\times {\mathbb {R}}$. Let us also denote by ${\widehat{\beta }}:{\mathbb {R}}\rightarrow [0,+\infty )$ the convex lower semicontinuous function with ${\widehat{\beta }}(0)=0$.
**A2:** $$\varphi _0 \in V_1$$ and $$\Psi (\varphi _0)\in L^1({\mathcal {O}})$$.
**A3:** $B:V_1\rightarrow {\mathscr {L}}^2(K,V_1)$ and there exists a constant $C_B>0$ such that
  $$\begin{aligned} \left\| B(y_1)-B(y_2)\right\| _{{\mathscr {L}}^2(K,H)} \le C_B\left\| y_1-y_2\right\| _{H}&\quad \forall \,y_1,y_2\in H\,,\\ \left\| B(y)\right\| _{{\mathscr {L}}^2(K,V_1)} \le C_B\left( 1+\left\| y\right\| _{V_1} \right)&\quad \forall \,y\in V_1\,,\\ \sum _{j=0}^\infty \left\| B(y)e_j\right\| _{L^{\frac{2\gamma }{\gamma -1}}({\mathcal {O}})}^2 \le C_B&\quad \forall \,y\in H . \end{aligned}$$
  Moreover, we prescribe that
  $$\begin{aligned} B:V_1\rightarrow {\mathscr {L}}^2(K,V_{1,0}) \qquad \text {in case of multiplicative noise} . \end{aligned}$$
  Let us note that in case of additive noise $B\in {\mathscr {L}}^2(K,V_1)$, these conditions are trivially satisfied for all $\gamma \in (1,2]$ if $d=2$ and for all $\gamma \in [3/2,2]$ if $d=3$: in particular, the classical polynomial case in dimension two and three is always covered. In the genuine multiplicative noise case, i.e. when *B* is not constant in $V_1$, we also suppose that *B* is ${\mathscr {L}}^2(K,V_{1,0})$-valued: this amounts to requiring that the noise is conservative, in the sense that it preserves the mean $\varphi _{\mathcal {O}}$ of the phase-variable. A direct consequence is the conservation of mass, which is a fundamental feature of Cahn–Hilliard-type evolutions. This hypothesis on the noise is very classical and natural in literature: for example, let us stress that a relevant multiplicative choice of *B* can be given as:
  $$\begin{aligned} B(y)e_j:=h_j(y) - (h_j(y))_{\mathcal {O}}\,, \quad y\in V_1\,,\quad j\in {\mathbb {N}}\,, \end{aligned}$$
  where the sequence $(h_j)_j\subset W^{1,\infty }({\mathbb {R}})$ is such that
  $$\begin{aligned} C_B^2:=\sum _{j=0}^\infty \left\| h_j\right\| ^2_{W^{1,\infty }({\mathbb {R}})}<+\infty . \end{aligned}$$
  It is not difficult to show that this example allows for all values of $\gamma \in [1,2]$ in every space-dimension $d=2,3$.

In the context of the optimal velocity control, it will be useful to introduce a polynomial-growth assumption on $\Psi$. This will be necessary only in the study of the optimisation problem, but is not needed for the well-posedness of the state system.

**C1:** it holds that $\gamma =2$ in **A1** and
  $$\begin{aligned} |\Psi ''(r)|\le C_\Psi (1+|r|^2) \qquad \forall \,r\in {\mathbb {R}}. \end{aligned}$$
  Such requirement is very natural in the Cahn–Hilliard context, since it is satisfied by the classical choice of the polynomial double-well potential $\Psi _{pol}$ of degree 4.

The first main result of the paper states existence and uniqueness of strong solutions, and their continuous dependence with respect to the velocity field.

### Theorem 2.1

Assume **A1–A3**. Then, for every ${{\mathbf {u}}}\in {\mathcal {U}}$, there exists a unique pair $(\varphi ,\mu )$ with

$$\begin{aligned}&\varphi \in L^p_{\mathscr {P}}\left( \Omega ; W^{s,p}(0,T; V_1^*)\cap C^0([0,T]; H) \cap L^2(0,T; V_2)\right) \cap L^p_w(\Omega ;L^\infty (0,T; V_1))\,,\\&\mu = -\Delta \varphi + \Psi '(\varphi )\in L^{p/2}_{\mathscr {P}}(\Omega ; L^2(0,T; V_1))\,, \end{aligned}$$

for all $s\in (0,1/2)$, and such that

$$\begin{aligned}&(\varphi (t),\zeta )_H + \int _{Q_t}\nabla \varphi \cdot \nabla \zeta -\int _{Q_t} \varphi {{\mathbf {u}}}\cdot \nabla \zeta \\&\quad = (\varphi _0,\zeta )_H + \left( \int _0^tB(\varphi (s))\,{\mathrm d}W(s), \zeta \right) _H \qquad \forall \,\zeta \in V_1\,, \end{aligned}$$

for every $t\in [0,T]$, ${\mathbb {P}}$-almost surely. Furthermore, there exists a constant $K>0$, only depending on the structure of the problem, such that for all ${{\mathbf {u}}}\in {\mathcal {U}}$, the respective solution $(\varphi ,\mu )$ satisfies

2.2 $$\begin{aligned}&\left\| \varphi \right\| _{L^p(\Omega ; L^\infty (0,T; V_1))\cap L^p_{\mathscr {P}}(\Omega ; L^2(0,T; V_2))} +\left\| \mu \right\| _{L^{p/2}_{\mathscr {P}}(\Omega ; L^2(0,T; V_1))}\nonumber \\&\quad +\left\| \Psi (\varphi )\right\| _{L^{p/2}(\Omega ;L^\infty (0,T; L^1({\mathcal {O}})))}\nonumber \\&\quad +\left\| \Psi '(\varphi )\right\| _{L^{p/2}_{\mathscr {P}}(\Omega ; L^2(0,T; H))} + \left\| \Psi ''(\varphi )\right\| _{L^{\gamma p/2}(\Omega ;L^\infty (0,T; L^\gamma ({\mathcal {O}})))}\nonumber \\&\quad \le K\left[ 1 + \left\| {{\mathbf {u}}}\right\| _{{\mathcal {U}}}^{\frac{2}{p-2}}\right] \,, \end{aligned}$$

and for every $\{{{\mathbf {u}}}_i\}_{i=1,2}\subset {\mathcal {U}}$, the respective solutions $\{(\varphi _i, \mu _i)\}_{i=1,2}$ verify

2.3 $$\begin{aligned}&\left\| \varphi _1-\varphi _2\right\| _{L^p_{\mathscr {P}}(\Omega ; C^0([0,T]; V_1^*)\cap L^2(0,T; V_1))} \nonumber \\&\quad \le K \left[ 1+\left\| {{\mathbf {u}}}_1\right\| _{{\mathcal {U}}}^{\frac{2}{p-2}}\right] \left[ 1+\left\| {{\mathbf {u}}}_2\right\| _{{\mathcal {U}}}^{\frac{2}{p-2}}\right] \left\| {{\mathbf {u}}}_1-{{\mathbf {u}}}_2\right\| _{{\mathcal {U}}} . \end{aligned}$$

Lastly, if also **C1** holds, then

2.4 $$\begin{aligned}&\left\| \varphi _1-\varphi _2\right\| _{L^{p/3}_{\mathscr {P}}(\Omega ; C^0([0,T]; H)\cap L^2(0,T; V_2))} + \left\| \mu _1-\mu _2\right\| _{L^{p/3}_{\mathscr {P}}(\Omega ; L^2(0,T; H))}\nonumber \\&\quad \le K\left[ 1 + \left\| {{\mathbf {u}}}_1\right\| _{{\mathcal {U}}}^{\frac{4}{p-2}} + \left\| {{\mathbf {u}}}_2\right\| _{{\mathcal {U}}}^{\frac{4}{p-2}}\right] \left[ 1+\left\| {{\mathbf {u}}}_1\right\| _{{\mathcal {U}}}^{\frac{2}{p-2}}\right] \left[ 1+\left\| {{\mathbf {u}}}_2\right\| _{{\mathcal {U}}}^{\frac{2}{p-2}}\right] \left\| {{\mathbf {u}}}_1-{{\mathbf {u}}}_2\right\| _{{\mathcal {U}}} . \end{aligned}$$

Once the analysis of well-posedness of the state system has been addressed, we can turn our attention to the optimal velocity control problem. As far as the controls are concerned, we consider classical box-constraints on the velocity controls, by defining the set of admissible controls as:

$$\begin{aligned} {\mathcal {U}}_{ad}:=\left\{ {{\mathbf {u}}}\in {\mathcal {U}}:\;\left\| {{\mathbf {u}}}\right\| _{L^p(0,T; U)} \le L \quad {\mathbb {P}}\text {-a.s.}\right\} \,, \end{aligned}$$

where $L>0$ is a prescribed constant. The prescription of a box-constraint on the admissible controls is classical on the mathematical side. In applications, the constant *L* is typically related to the maximum capacity of the flow-inducing devices that convey the velocity field. It will be useful to introduce an enlarged bounded open set $\widetilde{{\mathcal {U}}}_{ad}$ in ${\mathcal {U}}$ containing ${\mathcal {U}}_{ad}$, as

$$\begin{aligned} \widetilde{{\mathcal {U}}}_{ad}:=\left\{ {{\mathbf {u}}}\in {\mathcal {U}}:\;\left\| {{\mathbf {u}}}\right\| _{{\mathcal {U}}} < L+1\right\} . \end{aligned}$$

Analogously, we introduce the corresponding spaces of admissible deterministic controls as:

$$\begin{aligned} {\mathcal {U}}_{ad}^{det}:={\mathcal {U}}^{det}\cap {\mathcal {U}}_{ad}\,,\qquad {\widetilde{{\mathcal {U}}}}_{ad}^{det}:={\mathcal {U}}^{det}\cap \widetilde{{\mathcal {U}}}_{ad} . \end{aligned}$$

The cost functional that we study is of quadratic tracking-type and reads

2.5 $$\begin{aligned}&J:L^2_{\mathscr {P}}(\Omega ;C^0([0,T]; H))\times L^2_{\mathscr {P}}(\Omega ; L^2(0,T; H^d)) \rightarrow {\mathbb {R}}\,,\nonumber \\&J(\varphi ,{{\mathbf {u}}}):=\frac{\alpha _1}{2}{{\mathbb {E}}}\int _Q|\varphi -\varphi _Q|^2 +\frac{\alpha _2}{2}{{\mathbb {E}}}\int _{\mathcal {O}}|\varphi (T) - \varphi _T|^2 +\frac{\alpha _3}{2}{{\mathbb {E}}}\int _Q|{{\mathbf {u}}}|^2\,,\nonumber \\&\quad \;\;(\varphi ,{{\mathbf {u}}})\in L^2_{\mathscr {P}}(\Omega ;C^0([0,T]; H))\times L^2_{\mathscr {P}}(\Omega ; L^2(0,T; H^d)) \,, \end{aligned}$$

where $\alpha _1,\alpha _2,\alpha _3$ are non-negative constants with $\alpha _1+\alpha _2+\alpha _3>0$ and the targets are fixed with

$$\begin{aligned} \varphi _Q \in L^2_{\mathscr {P}}(\Omega ; L^2(0,T; H))\,, \qquad \alpha _2\varphi _T\in L^2(\Omega ,{\mathscr {F}}_T; H) . \end{aligned}$$

The optimal velocity control consists in the following:

(CP) minimise the cost functional *J* with the constraints that ${{\mathbf {u}}}$ belongs to ${\mathcal {U}}_{ad}$ and $\varphi$ is the unique corresponding solution component to the state system (1.1)–(1.4).

By virtue of the well-posedness Theorem 2.1, it is well defined the *control-to-state map*

$$\begin{aligned} S:{\widetilde{{\mathcal {U}}}}_{ad}\rightarrow & {} \left[ L^p_{\mathscr {P}}\left( \Omega ; C^0([0,T]; H) \cap L^2(0,T; V_2)\right) \cap L^p_w(\Omega ;L^\infty (0,T; V_1))\right] \\&\quad \times L^{p/2}_{\mathscr {P}}(\Omega ; L^2(0,T; V_1)) \end{aligned}$$

as

$$\begin{aligned} S({{\mathbf {u}}})=(S_1({{\mathbf {u}}}), S_2({{\mathbf {u}}})):=(\varphi ,\mu )\,, \qquad {{\mathbf {u}}}\in {\widetilde{{\mathcal {U}}}}_{ad} . \end{aligned}$$

This implies that the optimal control problem can be reduced to the only variable ${{{\mathbf {u}}}}$, by introducing the so-called *reduced* cost functional as:

$$\begin{aligned} {\widetilde{J}}:{\widetilde{{\mathcal {U}}}}_{ad}\rightarrow {\mathbb {R}}\,, \qquad {\widetilde{J}}({{\mathbf {u}}}):=J(S_1({{\mathbf {u}}}), {{\mathbf {u}}})\,, \quad {{\mathbf {u}}}\in {\widetilde{{\mathcal {U}}}}_{ad} . \end{aligned}$$

### Remark 2.2

Clearly, the well-posedness result in Theorem 2.1 continues to hold on any new stochastic basis $(\Omega ', {\mathscr {F}}', {\mathbb {P}}', W')$, provided to analogously define the new spaces of controls ${\mathcal {U}}'$, ${\mathcal {U}}'_{ad}$, and ${\widetilde{{\mathcal {U}}}}_{ad}'$. Hence, if also $(\varphi _Q',\varphi _T')$ are some new targets on $(\Omega ', {\mathscr {F}}', {\mathbb {P}}')$ with the same law of $(\varphi _Q, \varphi _T)$, one can define the corresponding cost functional $J'$, the corresponding control-to-state map $S'$, and the new reduced cost functional ${\widetilde{J}}'$ on the new probability space, by simply replacing $\Omega$ with $\Omega '$.

With this notations, we can state the exact definition of optimal control as follows. As anticipated, we also give some relaxed notions of optimality, one based on the concept of optimality-in-law and the other obtained minimising only on the deterministic controls.

### Definition 2.3

An optimal control for **(CP)** is an element ${{\mathbf {u}}}\in {\mathcal {U}}_{ad}$ such that

$$\begin{aligned} {\widetilde{J}}({{\mathbf {u}}})= \inf _{{{\mathbf {v}}}\in {\mathcal {U}}_{ad}}{\widetilde{J}}({{\mathbf {v}}}). \end{aligned}$$

A relaxed optimal control for **(CP)** is a family $\left( \Omega ', {\mathscr {F}}', ({\mathscr {F}}'_t)_{t\in [0,T]}, {\mathbb {P}}', W',\varphi _Q', \varphi _T',{{\mathbf {u}}}'\right)$ where $(\Omega ', {\mathscr {F}}', {\mathbb {P}}')$ is a probability space, $({\mathscr {F}}'_t)_{t\in [0,T]}$ is a filtration satisfying the usual conditions, $W'$ is a *K*-cylindrical Wiener process on it, $\alpha _1\varphi _Q'\in L^2_{\mathscr {P}}(\Omega '; L^2(0,T; H))$ and $\alpha _2\varphi _T'\in L^2(\Omega ',{\mathscr {F}}_T'; H)$ have the same laws of $\alpha _1\varphi _Q$ and $\alpha _2\varphi _T$, respectively, and ${{\mathbf {u}}}'\in {\mathcal {U}}_{ad}'$ satisfies

$$\begin{aligned} {\widetilde{J}}'({{\mathbf {u}}}')= \inf _{{{\mathbf {v}}}\in {\mathcal {U}}_{ad}}{\widetilde{J}}({{\mathbf {v}}}). \end{aligned}$$

A deterministic optimal control for **(CP)** is an element ${{\mathbf {u}}}\in {\mathcal {U}}_{ad}^{det}$ such that

$$\begin{aligned} {\widetilde{J}}({{\mathbf {u}}})= \inf _{{{\mathbf {v}}}\in {\mathcal {U}}_{ad}^{det}}{\widetilde{J}}({{\mathbf {v}}}). \end{aligned}$$

Our first result in the analysis of the optimisation problem **(CP)** concerns existence optimal controls. It is worthwhile noting that due to the non-uniqueness of optimal controls, in the genuinely stochastic case one can only show existence of relaxed optimal controls: this is typical in highly nonlinear stochastic optimal control problems, see, for example, (Barbu et al. 2018; Scarpa 2019b). By contrast, we show that deterministic optimal controls always exist.

### Theorem 2.4

Assume **A1–A3**. Then, there exist a relaxed optimal control ${{\mathbf {u}}}$ and a deterministic optimal control ${{\mathbf {u}}}^{det}$ for problem **(CP)**.

Once existence of minimisers for **(CP)** is proved, we can now turn to the main focus of the work, i.e. the investigation of necessary conditions for optimality. The first main step in this direction is the study of the differentiability of the control-to-state map *S*, along with the characterisation of its derivative through the analysis of the linearised state system. This will allow to obtain a first version of the first-order conditions for optimality by means of a suitable variational inequality involving the derivative of the reduced cost functional. In this direction, we introduce the assumptions

**C2:** the map $B:V_1\rightarrow {\mathscr {L}}^2(K,H)$ is of class $C^1$. Let us point out that this implies together with **A3** that $\left\| DB(y)\zeta \right\| _{{\mathscr {L}}^2(K,H)}\le C_B\left\| \zeta \right\| _H$ for all $y,\zeta \in V_1$. Moreover, let us stress this requirement is very natural, and it is satisfied, for instance, in the relevant example described in **A3**, provided to replace $W^{1,\infty }({\mathbb {R}})$ with $W^{1,\infty }({\mathbb {R}})\cap C^1({\mathbb {R}})$.
**C3:** $\Psi$ is of class $C^3$, $DB\in C^{0,1}(V_1; {\mathscr {L}}(V_1,{\mathscr {L}}^2(K,H)))$, and it holds that
  $$\begin{aligned} |\Psi '''(r)|\le C_\Psi (1+|r|)\qquad \forall \,r\in {\mathbb {R}}. \end{aligned}$$
  This is a refinement of assumptions **C1–C2** and ensures, as we will see, better differentiability properties for *S*. Still, **C3** is satisfied by the polynomial potential $\Psi _{pol}$ and the relevant noise coefficient described in **A3**, provided to replace $W^{1,\infty }({\mathbb {R}})$ with $W^{2,\infty }({\mathbb {R}})$.

The linearised system can be formally obtained by differentiating the state system (1.1)–(1.4) with respect to the control ${{\mathbf {u}}}$ in a given direction ${{\mathbf {h}}}\in {\mathcal {U}}$, and reads

2.6 $$\begin{aligned} {\mathrm d}\theta _{{\mathbf {h}}}- \Delta \nu _{{\mathbf {h}}}\,{\mathrm d}t + {{\mathbf {h}}}\cdot \nabla \varphi \,{\mathrm d}t + {{\mathbf {u}}}\cdot \nabla \theta _{{\mathbf {h}}}\,{\mathrm d}t = DB(\varphi )\theta _{{\mathbf {h}}}\,{\mathrm d}W \qquad&\text {in } (0,T)\times {\mathcal {O}}\,, \end{aligned}$$

2.7 $$\begin{aligned} \nu _{{\mathbf {h}}}=-\Delta \theta _{{\mathbf {h}}}+ \Psi ''(\varphi )\theta _{{\mathbf {h}}}\qquad&\text {in } (0,T)\times {\mathcal {O}}\,, \end{aligned}$$

2.8 $$\begin{aligned} \mathbf{n}\cdot \nabla \theta _{{\mathbf {h}}}= \mathbf{n}\cdot \nabla \nu _{{\mathbf {h}}}= 0 \qquad&\text {in } (0,T)\times \partial {\mathcal {O}}\,, \end{aligned}$$

2.9 $$\begin{aligned} \theta _{{\mathbf {h}}}(0)=0 \qquad&\text {in } {\mathcal {O}}. \end{aligned}$$

The next result ensures exactly that the linearised system (2.6)–(2.9) is well posed in a suitable variational sense, and that the unique solution to (2.6)–(2.9) coincides with the derivative of the control-to-state map *S* in the point ${{\mathbf {u}}}$ along the direction ${{\mathbf {h}}}$.

### Theorem 2.5

Assume **A1–A3**, **C1–C2**, and $p>3$. Then, for all ${{\mathbf {u}}}\in {\widetilde{{\mathcal {U}}}}_{ad}$ and ${{\mathbf {h}}}\in {\mathcal {U}}$, setting $\varphi :=S_1({{\mathbf {u}}})$, there exists a unique pair $(\theta _{{\mathbf {h}}},\nu _{{\mathbf {h}}})$ with

$$\begin{aligned}&\theta _{{\mathbf {h}}}\in L^{p}_{{\mathscr {P}}}\left( \Omega ; C^0([0,T]; V_1^*)\cap L^2(0,T; V_1)\right) \cap L^{p/3}_{\mathscr {P}}\left( \Omega ; C^0([0,T]; H)\cap L^2(0,T; V_2)\right) \,,\\&\nu _{{\mathbf {h}}}= -\Delta \theta _{{\mathbf {h}}}+ \Psi ''(\varphi )\theta _{{\mathbf {h}}}\in L^{p/3}_{{\mathscr {P}}}(\Omega ; L^2(0,T; H))\,, \end{aligned}$$

such that, for every $t\in [0,T]$, ${\mathbb {P}}$-almost surely,

$$\begin{aligned}&(\theta _{{\mathbf {h}}}(t),\zeta )_H - \int _{Q_t}\nu _{{\mathbf {h}}}\Delta \zeta -\int _{Q_t}(\varphi {{\mathbf {h}}}+ \theta _{{\mathbf {h}}}{{\mathbf {u}}})\cdot \nabla \zeta \\&\quad = \left( \int _0^tDB(\varphi (s))\theta _{{\mathbf {h}}}(s)\,{\mathrm d}W(s), \zeta \right) _H \quad \forall \,\zeta \in V_2 . \end{aligned}$$

Furthermore, the control-to-state map $S_1$ is Gâteaux-differentiable in the following sense: for all ${{\mathbf {u}}}\in {\widetilde{{\mathcal {U}}}}_{ad}$ and ${{\mathbf {h}}}\in {\mathcal {U}}$, as $\delta \searrow 0$, it holds that

$$\begin{aligned} \frac{S_1({{\mathbf {u}}}+\delta {{\mathbf {h}}}) - S_1({{\mathbf {u}}})}{\delta }\rightarrow \theta _{{\mathbf {h}}}\qquad&\text {in } L^\ell _{\mathscr {P}}(\Omega ; L^2(0,T; V_1))\quad \forall \,\ell \in [1,p)\,,\\ \frac{S_1({{\mathbf {u}}}+\delta {{\mathbf {h}}}) - S_1({{\mathbf {u}}})}{\delta }{\mathop {\rightharpoonup }\limits ^{*}}\theta _{{\mathbf {h}}}\qquad&\text {in } L^{p}_w\left( \Omega ; L^\infty (0,T; V_1^*)\right) \cap L^{p}_{\mathscr {P}}\left( \Omega ; L^2(0,T; V_1)\right) \,,\\ \frac{S_1({{\mathbf {u}}}+\delta {{\mathbf {h}}}) - S_1({{\mathbf {u}}})}{\delta }{\mathop {\rightharpoonup }\limits ^{*}}\theta _{{\mathbf {h}}}\qquad&\text {in } L^{p/3}_w\left( \Omega ; L^\infty (0,T; H)\right) \cap L^{p/3}_{\mathscr {P}}\left( \Omega ; L^2(0,T; V_2)\right) \,,\\ \frac{S_1({{\mathbf {u}}}+\delta {{\mathbf {h}}})(t) - S_1({{\mathbf {u}}})(t)}{\delta }\rightharpoonup \theta _{{\mathbf {h}}}(t) \qquad&\text {in } L^{p/3}(\Omega , {\mathscr {F}}_t; H) \quad \forall \,t\in [0,T] . \end{aligned}$$

Moreover, if $p\ge 7$ and **C3** holds, then $S_1$ is also Fréchet-differentiable as a map

$$\begin{aligned} S_1:{\widetilde{{\mathcal {U}}}}_{ad}\rightarrow L^{p/7}_{\mathscr {P}}(\Omega ; C^0([0,T]; V_1^*)\cap L^2(0,T; V_1)) . \end{aligned}$$

The second step in the analysis of necessary conditions for optimality consists in studying the so-called adjoint system and by proving a suitable duality relation with respect to the linearised system. The adjoint system can be formally obtained as the dual system of (2.6)–(2.9), and reads

2.10 $$\begin{aligned} -{\mathrm d}P -\Delta {\tilde{P}} \,{\mathrm d}t + \Psi ''(\varphi ){\tilde{P}}\,{\mathrm d}t - {{\mathbf {u}}}\cdot \nabla P\,{\mathrm d}t \qquad \qquad&\nonumber \\ =\alpha _1(\varphi -\varphi _Q)\,{\mathrm d}t+ DB(\varphi )^*Z\,{\mathrm d}t - Z\,{\mathrm d}W \qquad&\text {in } (0,T)\times {\mathcal {O}}\,, \end{aligned}$$

2.11 $$\begin{aligned} {\tilde{P}}=-\Delta P \qquad&\text {in } (0,T)\times {\mathcal {O}}\,, \end{aligned}$$

2.12 $$\begin{aligned} \mathbf{n}\cdot \nabla P = \mathbf{n}\cdot \nabla {\tilde{P}} = 0 \qquad&\text {in } (0,T)\times \partial {\mathcal {O}}\,,\end{aligned}$$

2.13 $$\begin{aligned} P(T)=\alpha _2(\varphi (T)-\varphi _T) \qquad&\text {in } {\mathcal {O}}. \end{aligned}$$

Let us point out that the adjoint system is backward in time: due to the stochastic framework of the problem, this necessarily requires the introduction of the additional variable *Z* in view of the classical martingale representation theorems. The situation here is then much more complex than the deterministic one: the variable of the adjoint system is indeed the couple (*P*, *Z*), with ${\tilde{P}}$ being an auxiliary variable. Due to the difficulty of analysis of the adjoint system, we will need to require more regularity on the targets, namely

C4 $p\ge 6$ and it holds that
  $$\begin{aligned} \alpha _1\varphi _Q \in L^{\frac{2p}{p-4}}_{\mathscr {P}}(\Omega ; L^2(0,T; H)), \qquad \alpha _2\varphi _T\in L^{\frac{2p}{p-4}}(\Omega ,{\mathscr {F}}_T; V_1). \end{aligned}$$

The next result ensures that the adjoint system (2.10)–(2.13) is well posed in a suitable variational sense, and state a duality relation between (2.6)–(2.9) and (2.10)–(2.13).

### Theorem 2.6

Assume **A1–A3**, **C1–C2**, and **C4**. Then, for all ${{\mathbf {u}}}\in {\widetilde{{\mathcal {U}}}}_{ad}$, setting $\varphi :=S_1({{\mathbf {u}}})$, there exists a triplet $(P, {\tilde{P}},Z)$, with

$$\begin{aligned}&P\in L^2_{\mathscr {P}}(\Omega ; C^0([0,T]; V_1)\cap L^2(0,T; V_3))\,,\\&{\tilde{P}}={\mathcal {L}} P \in L^2_{\mathscr {P}}(\Omega ; C^0([0,T]; V_1^*)\cap L^2(0,T; V_1))\,,\\&Z \in L^2_{\mathscr {P}}(\Omega ; L^2(0,T; {\mathscr {L}}^2(K,V_1)))\,, \end{aligned}$$

such that, for every $t\in [0,T]$, ${\mathbb {P}}$-almost surely,

$$\begin{aligned}&\left( P(t), \zeta \right) _H +\int _{Q_t^T}\nabla {\tilde{P}}\cdot \nabla \zeta +\int _{Q_t^T}\Psi ''(\varphi ){\tilde{P}}\zeta +\int _{Q_t^T}P{{\mathbf {u}}}\cdot \nabla \zeta \\&\quad =\left( \alpha _2(\varphi (T)-\varphi _T), \zeta \right) _H +\int _{Q_t^T}DB(\varphi )^*Z\zeta -\left( \int _t^TZ(s)\,{\mathrm d}W(s), \zeta \right) _H \qquad \forall \,\zeta \in V_1. \end{aligned}$$

Furthermore, the solution components $\nabla P$, ${\tilde{P}}$, and $\nabla Z$ are unique in the spaces $L^2_{\mathscr {P}}(\Omega ; C^0([0,T]; H^d))$, $L^2_{\mathscr {P}}(\Omega ; C^0([0,T]; V_1^*))$, and $L^2_{\mathscr {P}}(\Omega ; L^2(0,T; {\mathscr {L}}^2(K,H^d)))$, respectively.

At this point, we are finally ready to state the necessary conditions for optimality: more specifically, we present here two different versions. The first one is deduced directly by the characterisation of the derivative of $S_1$ in Theorem 2.5, and consists of a variational inequality depending also on the linearised variables. The second one is a refinement of this, as it employs the adjoint problem and only depends on the intrinsic adjoint variables $(P,{\tilde{P}}, Z)$, not on the linearised ones.

### Theorem 2.7

Assume **A1–A3**, **C1–C2**, and $p\ge 6$. If ${{\mathbf {u}}}\in {\mathcal {U}}_{ad}$ is an optimal control for **(CP)** and $\varphi :=S_1({{\mathbf {u}}})$ is its respective optimal state, then

2.14 $$\begin{aligned}&\alpha _1{{\mathbb {E}}}\int _Q(\varphi - \varphi _Q) \theta _{{{\mathbf {v}}}-{{\mathbf {u}}}} + \alpha _2{{\mathbb {E}}}\int _{\mathcal {O}}(\varphi (T)-\varphi _T)\theta _{{{\mathbf {v}}}-{{\mathbf {u}}}}(T)\nonumber \\&\quad +\alpha _3{{\mathbb {E}}}\int _Q{{\mathbf {u}}}\cdot ({{\mathbf {v}}}-{{\mathbf {u}}}) \ge 0 \qquad \forall \,{{\mathbf {v}}}\in {\mathcal {U}}_{ad}\,, \end{aligned}$$

where $\theta _{{{\mathbf {v}}}-{{\mathbf {u}}}}$ is the unique first solution component of the linearised system (2.6)–(2.9) with the choice ${{\mathbf {h}}}:={{\mathbf {v}}}-{{\mathbf {u}}}$, in the sense of Theorem 2.5.

### Theorem 2.8

Assume **A1–A3**, **C1–C2**, and **C4**. If ${{\mathbf {u}}}\in {\mathcal {U}}_{ad}$ is an optimal control for **(CP)** and $\varphi :=S_1({{\mathbf {u}}})$ is its respective optimal state, then

2.15 $$\begin{aligned} {{\mathbb {E}}}\int _Q(\varphi \nabla P + \alpha _3{{\mathbf {u}}})\cdot ({{\mathbf {v}}}-{{\mathbf {u}}}) \ge 0 \qquad \forall \,{{\mathbf {v}}}\in {\mathcal {U}}_{ad}\,, \end{aligned}$$

where $\nabla P$ is the uniquely determined solution component of the adjoint system (2.10)–(2.13) in the sense of Theorem 2.6. In particular, if $\alpha _3>0$, then ${{\mathbf {u}}}$ is the orthogonal projection of $-\frac{1}{\alpha _3}\varphi \nabla P$ on the closed convex set ${\mathcal {U}}_{ad}$ in the Hilbert space $L^2_{\mathscr {P}}(\Omega ;L^2(0,T; H^d))$.

### Remark 2.9

Let us comment on the necessary condition for optimality. When handling the optimisation problem in practice, the main role of condition (2.15) is to restrict the class of possible candidates to be optimal controls. Roughly speaking, the optimisation analysis begins with the identification of some natural candidates ${{\mathbf {u}}}$ to the role of optimal controls. Secondly, for such controls ${{\mathbf {u}}}$ the forward and the backward systems are solved, so that the respective variables $\varphi =\varphi ({{\mathbf {u}}})$ and $\nabla P=\nabla P({{\mathbf {u}}})$ are identified. Finally, if condition (2.15) is not met, then the candidate ${{\mathbf {u}}}$ is cut off from the analysis, otherwise it is confirmed. Nonetheless, let stress again that condition (2.15) is only a necessary requirement, and can only help to restrict the class of potential optimal controls. In order to further refine the analysis, sufficient conditions for optimality should be investigated. The mathematical idea behind this is very natural: if the reduced cost functional ${\widetilde{J}}$ can be shown to be twice (Fréchet or Gâteaux) differentiable, then any control ${{\mathbf {u}}}$ satisfying the first-order stationary condition (2.15) and the positive definiteness condition $D^2{\widetilde{J}}({{\mathbf {u}}})>0$ is an optimal control. Such second-order analysis is extremely challenging, and to the best of the author’s knowledge, it has been performed so far only in relation to some selected optimal control problems in the deterministic setting (Colli et al. 2015b; Colli and Sprekels 2015). In the stochastic case, the second-order analysis is open and is currently being investigated in a work in preparation.

## Well-posedness of the State System

This section is devoted to the proof of Theorem 2.1 about well-posedness of the state system.

### Uniqueness

Let $\{{{\mathbf {u}}}_i\}_{i=1,2}\subset {\mathcal {U}}$ and let us denote by $\{(\varphi _i, \mu _i)\}_{i=1,2}$ any respective solutions to (1.1)–(1.4) in the sense of Theorem 2.1. Let us set for brevity of notation $\varphi :=\varphi _1-\varphi _2$, $\mu :=\mu _1-\mu _2$, ${{\mathbf {u}}}:={{\mathbf {u}}}_1-{{\mathbf {u}}}_2$: then we have

$$\begin{aligned} {\mathrm d}\varphi - \Delta \mu \,{\mathrm d}t + {{\mathbf {u}}}\cdot \nabla \varphi _1\,{\mathrm d}t + {{\mathbf {u}}}_2\cdot \nabla \varphi \,{\mathrm d}t = (B(\varphi _1)-B(\varphi _2))\,{\mathrm d}W\,, \qquad \varphi (0)=0\,, \end{aligned}$$

where the equality is intended in the usual variational sense of Theorem 2.1.

Taking $\frac{1}{|{\mathcal {O}}|}\in V_1$ as test function yields directly by assumption **A3** that $\varphi _{\mathcal {O}}=0$, so that actually $\varphi \in L^p_{\mathscr {P}}(\Omega ; C^0([0,T]; V_{1,0}^*))$ and $B(\varphi _1)-B(\varphi _2)\in L^p_{\mathscr {P}}(\Omega ; L^2(0,T; {\mathscr {L}}^2(K,V_{1,0}^*)))$. Hence, Itô’s formula for the function $\frac{1}{2}\left\| \nabla {\mathcal {N}}\varphi \right\| _H^2$ yields

$$\begin{aligned}&\frac{1}{2}\left\| \nabla {\mathcal {N}}\varphi (t)\right\| _{H}^2 +\int _{Q_t}|\nabla \varphi |^2 + \int _{Q_t}(\Psi '(\varphi _1)-\Psi '(\varphi _2))\varphi \\&\qquad +\int _{Q_t}({{\mathbf {u}}}\cdot \nabla \varphi _1+ {{\mathbf {u}}}_2\cdot \nabla \varphi ) {\mathcal {N}}\varphi \\&\quad =\frac{1}{2}\int _0^t\left\| \nabla {\mathcal {N}} (B(\varphi _1)-B(\varphi _2))(s)\right\| _{{\mathscr {L}}^2(K,H)}^2\,{\mathrm d}s\\&\qquad +\int _0^t\left( {\mathcal {N}}\varphi (s), (B(\varphi _1)-B(\varphi _2))(s)\,{\mathrm d}W(s)\right) _H. \end{aligned}$$

Now, the mean value theorem and assumption **A1** give

$$\begin{aligned} \int _{Q_t}(\Psi '(\varphi _1)-\Psi '(\varphi _2))\varphi \ge - C_\Psi \int _{Q_t}|\varphi |^2\,, \end{aligned}$$

while the inclusion $V_1\hookrightarrow L^6({\mathcal {O}})$, the Hölder and the Poincaré–Wirtinger inequalities yield

$$\begin{aligned}&\int _{Q_t}({{\mathbf {u}}}\cdot \nabla \varphi _1+ {{\mathbf {u}}}_2\cdot \nabla \varphi ){\mathcal {N}}\varphi \\&\quad \le c\int _0^t\left( \left\| \nabla \varphi _1(s)\right\| _H\left\| {{\mathbf {u}}}(s)\right\| _U+ \left\| {{\mathbf {u}}}_2(s)\right\| _U\left\| \nabla \varphi (s)\right\| _H\right) \left\| {\mathcal {N}}\varphi (s)\right\| _{V_1}\,{\mathrm d}s\\&\quad \le \left\| \varphi _1\right\| _{L^\infty (0,T; V_1)}^2\left\| {{\mathbf {u}}}\right\| _{L^2(0,T; U)}^2 +\frac{1}{2}\int _{Q_t}|\nabla \varphi |^2 +c\int _0^t\left( 1+ \left\| {{\mathbf {u}}}_2(s)\right\| _U^2\right) \left\| \nabla {\mathcal {N}}\varphi (s)\right\| ^2_{H}\,{\mathrm d}s. \end{aligned}$$

Furthermore, assumption **A3** ensures that

$$\begin{aligned} \int _0^t\left\| \nabla {\mathcal {N}} (B(\varphi _1)-B(\varphi _2))(s)\right\| _{{\mathscr {L}}^2(K,H)}^2\,{\mathrm d}s \le c\int _{Q_t}|\varphi |^2. \end{aligned}$$

Using the compactness inequality (2.1) and rearranging the terms, we are left with

3.1 $$\begin{aligned} \left\| \nabla {\mathcal {N}}\varphi (t)\right\| _{H}^2 +\int _{Q_t}|\nabla \varphi |^2&\le c\left\| \varphi _1\right\| _{L^\infty (0,T; V_1)}^2\left\| {{\mathbf {u}}}\right\| _{L^2(0,T; U)}^2\nonumber \\&\quad + c\int _0^t\left( 1+ \left\| {{\mathbf {u}}}_2(s)\right\| _U^2\right) \left\| \nabla {\mathcal {N}}\varphi (s)\right\| ^2_{H}\,{\mathrm d}s\nonumber \\&\quad +c\int _0^t\left( {\mathcal {N}}\varphi (s), (B(\varphi _1)-B(\varphi _2))(s)\,{\mathrm d}W(s)\right) _H. \end{aligned}$$

On the right-hand side, we have, by the Hölder inequality in time,

$$\begin{aligned} \int _0^t\left( 1+ \left\| {{\mathbf {u}}}_2(s)\right\| _U^2\right) \left\| \nabla {\mathcal {N}}\varphi (s)\right\| ^2_{H}\,{\mathrm d}s \le c t^{1-\frac{2}{p}}\left( 1+\left\| {{\mathbf {u}}}\right\| _{L^p(0,T; U)}^2\right) \left\| \nabla {\mathcal {N}}\varphi \right\| ^2_{L^\infty (0,t;H)}\,, \end{aligned}$$

and, thanks to the Burkholder–Davis–Gundy and the Young inequalities, assumption **A3**, and again the compactness inequality (2.1),

$$\begin{aligned}&{{\mathbb {E}}}\sup _{r\in [0,t]}\left| \int _0^r\left( {\mathcal {N}}\varphi (s), (B(\varphi _1)-B(\varphi _2))(s)\,{\mathrm d}W(s)\right) _H\right| ^{p/2} \\&\quad \le \frac{1}{8}{{\mathbb {E}}}\left\| \nabla {\mathcal {N}}\varphi \right\| _{L^\infty (0,t; H)}^p + c {{\mathbb {E}}}\left\| \varphi \right\| ^p_{L^2(0,t; H)}\\&\quad \le \frac{1}{8}{{\mathbb {E}}}\left\| \nabla {\mathcal {N}}\varphi \right\| _{L^\infty (0,t; H)}^p+ \frac{1}{2}{{\mathbb {E}}}\left\| \nabla \varphi \right\| ^p_{L^2(0,t; H)} + c {{\mathbb {E}}}\left\| \nabla {\mathcal {N}}\varphi \right\| ^p_{L^2(0,t; H)}. \end{aligned}$$

Consequently, taking power *p*/2 at both sides of (3.1) and rearranging the terms yield

$$\begin{aligned}&{{\mathbb {E}}}\left\| \nabla {\mathcal {N}}\varphi \right\| _{L^\infty (0,t;H)}^p +{{\mathbb {E}}}\left\| \nabla \varphi \right\| _{L^2(0,t; H)}^p\\&\quad \le c\left\| {{\mathbf {u}}}\right\| _{{\mathcal {U}}}^p{{\mathbb {E}}}\left\| \varphi _1\right\| _{L^\infty (0,T; V_1)}^p + c t^{\frac{p}{2}-1}(1+\left\| {{\mathbf {u}}}_2\right\| _{\mathcal {U}}^p){{\mathbb {E}}}\left\| \nabla {\mathcal {N}} \varphi \right\| _{L^\infty (0,t;H)}^p. \end{aligned}$$

Hence, setting

$$\begin{aligned} T_0:=\left( \frac{1}{2}c^{-1}(1+\left\| {{\mathbf {u}}}_2\right\| _{\mathcal {U}}^p)^{-1}\right) ^{\frac{2}{p-2}}\wedge T\,, \end{aligned}$$

we get

$$\begin{aligned}&{{\mathbb {E}}}\left\| \nabla {\mathcal {N}}\varphi \right\| _{L^\infty (0,T_0;H)}^p +{{\mathbb {E}}}\left\| \nabla \varphi \right\| _{L^2(0,T_0; H)}^p\\&\quad \le c\left\| {{\mathbf {u}}}\right\| _{{\mathcal {U}}}^p{{\mathbb {E}}}\left\| \varphi _1\right\| _{L^\infty (0,T; V_1)}^p + \frac{1}{2}{{\mathbb {E}}}\left\| \nabla {\mathcal {N}}\varphi \right\| _{L^\infty (0,T_0;H)}^p. \end{aligned}$$

Since $T_0$ is independent of the initial time, we can iterate the procedure and close the estimate on each subinterval $[kT_0, (k+1)T_0]$ for all $k\in {\mathbb {N}}$ until $(k+1)T_0>T$: summing up, noting that the number of such subintervals is less than $\frac{T}{T_0}+1$, and renominating *c* independently of ${{\mathbf {u}}}_2$, we get then

$$\begin{aligned}&\left\| \varphi _1-\varphi _2\right\| _{L^p_{\mathscr {P}}(\Omega ; C^0([0,T]; V_1^*)\cap L^2(0,T; V_1))}^p \\&\quad \le c\left\| \varphi _1\right\| _{L^p(\Omega ;L^\infty (0,T; V_1))}^p \left( 1 + \left\| {{\mathbf {u}}}_2\right\| _{\mathcal {U}}^{\frac{2p}{p-2}}\right) \left\| {{\mathbf {u}}}_1-{{\mathbf {u}}}_2\right\| ^p_{{\mathcal {U}}}\,, \end{aligned}$$

from which uniqueness of solutions follows.

### Approximation

We turn now to existence of solutions. First of all, for every $\lambda$ let $\beta _\lambda :{\mathbb {R}}\rightarrow {\mathbb {R}}$ be the Yosida approximation of $\beta$ and ${\widehat{\beta }}_\lambda :{\mathbb {R}}\rightarrow [0,+\infty )$ be the Moreau–Yosida regularisation of ${\widehat{\beta }}$, which are defined, respectively, as:

$$\begin{aligned} \beta _\lambda (r):=\frac{r-(I+\lambda \beta )^{-1}(r)}{\lambda }\,, \qquad {\widehat{\beta }}_\lambda (r):=\int _0^r\beta _\lambda (s)\,{\mathrm d}s\,, \qquad r\in {\mathbb {R}}. \end{aligned}$$

Let us recall that $\beta _\lambda$ is $\frac{1}{\lambda }$-Lipschitz continuous, ${\widehat{\beta }}_\lambda$ is convex and quadratic at $\infty$, and as $\lambda \searrow 0$ it holds that $\beta _\lambda (r)\rightarrow \beta (r)$ and ${\widehat{\beta }}_\lambda (r)\nearrow {\widehat{\beta }}(r)$ for all $r\in {\mathbb {R}}$. For further details about the properties of $\beta _\lambda$ and ${\widehat{\beta }}_\lambda$, we refer to the monograph (Barbu 2010, Ch. 2). We define the approximated double-well potential as:

$$\begin{aligned} \Psi _\lambda :{\mathbb {R}}\rightarrow {\mathbb {R}}\,, \qquad \Psi _\lambda (r):=\Psi (0) + {\widehat{\beta }}_\lambda (r) - \frac{C_\Psi }{2}r^2\,, \quad r\in {\mathbb {R}}\,, \end{aligned}$$

so that in particular we have $\Psi _\lambda '(r)=\beta _\lambda (r) - C_\Psi r$ for $r\in {\mathbb {R}}$. Secondly, we define

$$\begin{aligned} {{\mathbf {u}}}_\lambda := \rho _\lambda *{{\mathbf {u}}}\,, \end{aligned}$$

where $(\rho _\lambda )_\lambda \subset C^\infty _c({\mathbb {R}})$ is a classical non-anticipative sequence of mollifiers in time. In particular, let us point out that it holds

$$\begin{aligned} {{\mathbf {u}}}_\lambda \in L^\infty _{\mathscr {P}}(\Omega \times (0,T); U)\,, \qquad {{\mathbf {u}}}_\lambda \rightarrow {{\mathbf {u}}}\quad \text {in }L^q_{\mathscr {P}}(\Omega ; L^p(0,T; U))\quad \forall \,q\ge 1. \end{aligned}$$

The approximated system is obtained by replacing $\Psi '$ with $\Psi '_\lambda$ and ${{\mathbf {u}}}$ with ${{\mathbf {u}}}_\lambda$ in (1.1)–(1.4):

3.2 $$\begin{aligned} {\mathrm d}\varphi _\lambda - \Delta \mu _\lambda \,{\mathrm d}t + {{\mathbf {u}}}_\lambda \cdot \nabla \varphi _\lambda \,{\mathrm d}t = B(\varphi _\lambda )\,{\mathrm d}W \qquad&\text {in } (0,T)\times {\mathcal {O}}\,, \end{aligned}$$

3.3 $$\begin{aligned} \mu _\lambda = -\Delta \varphi _\lambda + \Psi _\lambda '(\varphi _\lambda ) \qquad&\text {in } (0,T)\times {\mathcal {O}}\,,\end{aligned}$$

3.4 $$\begin{aligned} \mathbf{n}\cdot \nabla \varphi _\lambda = \mathbf{n}\cdot \nabla \mu _\lambda = 0 \qquad&\text {in } (0,T)\times \partial {\mathcal {O}}\,,\end{aligned}$$

3.5 $$\begin{aligned} \varphi _\lambda (0)=\varphi _0 \qquad&\text {in } {\mathcal {O}}. \end{aligned}$$

We formulate (3.2)–(3.5) in an abstract way as

3.6 $$\begin{aligned} {\mathrm d}\varphi _\lambda + ({\mathcal {A}}_\lambda + {\mathcal {C}}_\lambda )(\varphi _\lambda )\,{\mathrm d}t = B(\varphi _\lambda )\,{\mathrm d}W\,, \qquad \varphi _\lambda (0)=\varphi _0\,, \end{aligned}$$

where the variational operators

$$\begin{aligned} {\mathcal {A}}_\lambda :V_2\rightarrow V_2^*\,,\qquad {\mathcal {C}}_\lambda :\Omega \times [0,T]\times V_2\rightarrow V_2^*\,, \end{aligned}$$

are defined as:

$$\begin{aligned} \left\langle {\mathcal {A}}_\lambda (y),\zeta \right\rangle :=\int _{\mathcal {O}}(-\Delta \zeta )(-\Delta y + \Psi '_\lambda (y))\,, \quad y,\zeta \in V_2\,, \end{aligned}$$

and

$$\begin{aligned} \left\langle {\mathcal {C}}_\lambda (\omega ,t,y),\zeta \right\rangle := - \int _{\mathcal {O}}y {{\mathbf {u}}}_\lambda (\omega ,t) \cdot \nabla \zeta \,, \qquad y,\zeta \in V_2\,,\quad t\in [0,T]. \end{aligned}$$

Since $\Psi '_\lambda$ is Lipschitz-continuous, it is not difficult to show (see, for example, Scarpa 2018, Lem. 3.1) that ${\mathcal {A}}_\lambda$ is weakly monotone, weakly coercive, and linearly bounded, in the sense that there are two constants $c_\lambda , c_\lambda '>0$ such that

$$\begin{aligned} \left\langle {\mathcal {A}}_\lambda (y_1)-{\mathcal {A}}_2(y_2),y_1-y_2\right\rangle \ge c_\lambda \left\| y_1-y_2\right\| _{V_2}^2 - c_\lambda '\left\| y_1-y_2\right\| _H^2 \qquad \forall \,y_1,y_2\in V_2 \end{aligned}$$

and

$$\begin{aligned} \left\| {\mathcal {A}}_\lambda (y)\right\| _{V_2^*} \le c_\lambda '(1+\left\| y\right\| _{V_2}) \qquad \forall \,y\in V_2. \end{aligned}$$

As far as the convection operator ${\mathcal {C}}_\lambda$ is concerned, since ${\text {div}}{{\mathbf {u}}}_\lambda =0$, thanks to the divergence theorem we have

$$\begin{aligned} \left\langle {\mathcal {C}}_\lambda (y_1)-{\mathcal {C}}_\lambda (y_2),y_1-y_2\right\rangle&= - \int _{\mathcal {O}}(y_1-y_2) {{\mathbf {u}}}_\lambda \cdot \nabla (y_1-y_2)=0\,, \end{aligned}$$

and, thanks to the Hölder inequality and the inclusion $V_1\hookrightarrow L^6({\mathcal {O}})$,

$$\begin{aligned} \left\| C_\lambda (y)\right\| _{V_2^*}= & {} \sup _{\left\| \zeta \right\| _{V_2}\le 1}\left\{ - \int _{\mathcal {O}}y {{\mathbf {u}}}_\lambda \cdot \nabla \zeta \right\} \le \left\| y\right\| _{H}\left\| {{\mathbf {u}}}_\lambda \right\| _{U}\\\le & {} \left\| {{\mathbf {u}}}_\lambda \right\| _{L^\infty _{\mathscr {P}}(\Omega \times (0,T); U)}\left\| y\right\| _{V_2} \quad \forall \,y\in V_2. \end{aligned}$$

Hence, the operator ${\mathcal {A}}_\lambda +{\mathcal {C}}_\lambda :\Omega \times [0,T]\times V_2\rightarrow V_2^*$ is weakly monotone, weakly coercive, and linearly bounded. Besides, due to the Lipschitz-continuity of $\Psi _\lambda '$ and the regularity of ${{\mathbf {u}}}_\lambda$, it is immediate to check that it is also hemicontinuous. Moreover, assumption **A3** ensures that $B: H\rightarrow {\mathscr {L}}^2(K,H)$ is Lipschitz-continuous. It follows then by the classical variational approach to SPDEs by Pardoux (1975) and Krylov and Rozovskiĭ (1979) that the evolution equation (3.6) admits a unique variational solution

$$\begin{aligned} \varphi _\lambda \in L^2_{\mathscr {P}}(\Omega ; C^0([0,T]; H)\cap L^2(0,T; V_2)). \end{aligned}$$

Let us set $\mu _\lambda :=-\Delta \varphi _\lambda + \Psi _\lambda '(\varphi _\lambda )$ as the approximated chemical potential.

### Uniform Estimates

Itô’s formula for the square of the *H*-norm yields

$$\begin{aligned}&\frac{1}{2}\left\| \varphi _\lambda (t)\right\| _H^2 +\int _{Q_t}|\Delta \varphi _\lambda |^2 + \int _{Q_t}\Psi _\lambda '(\varphi _\lambda )(-\Delta \varphi _\lambda ) -\int _{Q_t}\varphi _\lambda {{\mathbf {u}}}_\lambda \cdot \nabla \varphi _\lambda \\&\quad =\frac{1}{2}\left\| \varphi _0\right\| _H^2 +\frac{1}{2}\int _0^t\left\| B(\varphi _\lambda (s))\right\| _{{\mathscr {L}}^2(K,H)}^2\,{\mathrm d}s +\int _0^t\left( \varphi _\lambda (s), B(\varphi _\lambda (s))\,{\mathrm d}W(s)\right) _H. \end{aligned}$$

Now, on the left-hand side, we have, thanks to the monotonicity of $\beta _\lambda$,

$$\begin{aligned} \int _{Q_t}\Psi _\lambda '(\varphi _\lambda )(-\Delta \varphi _\lambda )= & {} \int _{Q_t}\beta _\lambda '(\varphi _\lambda )|\nabla \varphi _\lambda |^2 -C_\Psi \int _{Q_t}\varphi _\lambda (-\Delta \varphi _\lambda )\\\ge & {} -\frac{1}{4}\int _{Q_t}|\Delta \varphi _\lambda ^2| - C_\Psi ^2\int _{Q_t}|\varphi _\lambda |^2 . \end{aligned}$$

Also, by the Hölder inequality and the inclusion $V_1\hookrightarrow L^6({\mathcal {O}})$, it holds

$$\begin{aligned} -\int _{Q_t}\varphi _\lambda {{\mathbf {u}}}_\lambda \cdot \nabla \varphi _\lambda&\ge -\int _0^t\left\| \varphi _\lambda (s)\right\| _H\left\| {{\mathbf {u}}}_\lambda (s)\right\| _{U} \left\| \varphi (s)\right\| _{V_2}\,{\mathrm d}s. \end{aligned}$$

Thanks to the elliptic regularity theory for the Neumann problem (see, for example, Brezis 2011, §9.6) there is $c>0$ independent of $\lambda$ such that $\left\| \zeta \right\| _{V_2}\le c(\left\| \zeta \right\| _H+\left\| \Delta \zeta \right\| _H)$ for every $\zeta \in V_2$: consequently, renominating *c* and using the Young inequality we get

$$\begin{aligned} -\int _{Q_t}\varphi _\lambda {{\mathbf {u}}}_\lambda \cdot \nabla \varphi _\lambda \ge -\frac{1}{4}\int _{Q_t}|\Delta \varphi _\lambda |^2 -c^2\int _0^t\left\| \varphi _\lambda (s)\right\| _H^2(1+\left\| {{\mathbf {u}}}_\lambda (s)\right\| _{U}^2)\,{\mathrm d}s. \end{aligned}$$

Furthermore, noting that $\frac{2\gamma }{\gamma -1}\ge 4$ since $\gamma \in [1,2]$, assumption **A3** yields

$$\begin{aligned} \frac{1}{2}\int _0^t\left\| B(\varphi _\lambda (s))\right\| _{{\mathscr {L}}^2(K,H)}^2\,{\mathrm d}s \le c. \end{aligned}$$

Putting this information together and using assumption on the right-hand side we get, possibly updating the value of *c*,

$$\begin{aligned} \frac{1}{2}\left\| \varphi _\lambda (t)\right\| _H^2 +\frac{1}{2}\int _{Q_t}|\Delta \varphi _\lambda |^2&\le \frac{1}{2}\left\| \varphi _0\right\| _H^2 +c\int _0^t\left\| \varphi _\lambda (s)\right\| _H^2(1+\left\| {{\mathbf {u}}}_\lambda (s)\right\| _{U}^2)\,{\mathrm d}s\\&\quad +\int _0^t\left( \varphi _\lambda (s), B(\varphi _\lambda (s))\,{\mathrm d}W(s)\right) _H \qquad \forall \,t\in [0,T]\,,\quad {\mathbb {P}}\text {-a.s.} \end{aligned}$$

Taking now power *p*/2 at both sides, the stochastic integral on the right-hand side can be treated again thanks to **A3**, using classical computations based on the Burkholder–Davis–Gundy inequality (see, for example, Marinelli and Scarpa 2018, Lem. 4.3). Consequently, the same iterative argument used in Sect. 3.1 ensures that

3.7 $$\begin{aligned} \left\| \varphi _\lambda \right\| _{L^p_{\mathscr {P}}(\Omega ; C^0([0,T]; H)\cap L^2(0,T; V_2))}^p \le c\left( 1+\left\| {{\mathbf {u}}}\right\| _{{\mathcal {U}}}^{\frac{2p}{p-2}}\right) . \end{aligned}$$

In order to deduce further estimates on $\varphi _\lambda$ and $\mu _\lambda$, we rely on the free-energy estimate. Namely, we consider the approximated energy

$$\begin{aligned} \zeta \mapsto E_\lambda (\zeta ):= \frac{1}{2}\int _{\mathcal {O}}|\nabla \zeta |^2 + \int _{\mathcal {O}}\Psi _\lambda (\zeta )\,, \qquad \zeta \in V_1. \end{aligned}$$

Clearly, $E_\lambda$ is well defined and of class $C^1$ in $V_1$, with derivative

$$\begin{aligned} DE_\lambda :V_1\rightarrow V_1^*\,, \qquad DE_\lambda (\zeta )={\mathcal {L}}\zeta + \Psi '_\lambda (\zeta )\,, \quad \zeta \in V_1\,, \end{aligned}$$

so that in particular we have $DE_\lambda (\varphi _\lambda )= \mu _\lambda$. Moreover, the Lipschitz-continuity of $\Psi '_\lambda$ ensures that $DE_\lambda :V_1\rightarrow V_1^*$ is actually Fréchet-differentiable with

$$\begin{aligned} D^2E_\lambda (\zeta )[z_1,z_2]=\int _{\mathcal {O}}\nabla z_1\cdot \nabla z_2 +\int _{\mathcal {O}}\Psi _\lambda ''(\zeta )z_1z_2\,, \quad \zeta ,z_2,z_2\in V_1. \end{aligned}$$

Now, we would like to write Itô’s formula for $E_\lambda (\varphi _\lambda )$: in order to do this, we need to show first that $\varphi _\lambda$ and $\mu _\lambda$ enjoy more regularity. This can be shown by performing a further approximation on the problem (for example, the classical Faedo–Galerkin approximation of the abstract evolution equation (3.6)). Indeed, by the classical variational theory on stochastic evolution equations (Liu and Röckner 2015), there is a sequence $(H_n)_n$ of finite-dimensional subspaces of *H*, included in $V_2$ and with $\cup _nH_n$ dense in *H*, such that, setting $P_n:V_2^*\rightarrow H_n$ as the orthogonal projection onto $H_n$, the unique solution $(\varphi _\lambda ^n, \mu _\lambda ^n)$ of the finite-dimensional system

$$\begin{aligned} {\mathrm d}\varphi _\lambda ^n - \Delta \mu ^n_\lambda \,{\mathrm d}t + P_n({{\mathbf {u}}}_\lambda \cdot \nabla \varphi _\lambda ^n)\,{\mathrm d}t = P_nB(\varphi _\lambda ^n)\,{\mathrm d}W \qquad&\text {in } (0,T)\times {\mathcal {O}}\,,\\ \mu _\lambda ^n = -\Delta \varphi _\lambda ^n + P_n\Psi _\lambda '(\varphi _\lambda ^n) \qquad&\text {in } (0,T)\times {\mathcal {O}}\,,\\ \mathbf{n}\cdot \nabla \varphi _\lambda ^n = \mathbf{n}\cdot \nabla \mu _\lambda ^n= 0 \qquad&\text {in } (0,T)\times \partial {\mathcal {O}}\,,\\ \varphi ^n_\lambda (0)=\varphi _0^n \qquad&\text {in } {\mathcal {O}}\,, \end{aligned}$$

satisfy, as $$n\rightarrow \infty $$,

$$\begin{aligned} \varphi _\lambda ^n\rightharpoonup \varphi _\lambda \quad \text {in } L^p_{\mathscr {P}}(\Omega ; L^2(0,T; V_2))\,, \qquad \mu _\lambda ^n\rightharpoonup \mu _\lambda \quad \text {in } L^p_{\mathscr {P}}(\Omega ; L^2(0,T; H)). \end{aligned}$$

At this point, the finite-dimensional Itô formula for ${E_\lambda }_{|H_n}$ yields

$$\begin{aligned}&\frac{1}{2}\int _{\mathcal {O}}|\nabla \varphi _\lambda ^n(t)|^2 +\int _{\mathcal {O}}\Psi _\lambda (\varphi _\lambda ^n(t)) +\int _{Q_t}|\nabla \mu _\lambda ^n|^2 = \frac{1}{2}\int _{\mathcal {O}}|\nabla \varphi _0^n|^2 +\int _{\mathcal {O}}\Psi _\lambda (\varphi _0^n)\\&\quad +\int _{Q_t}\varphi _\lambda ^n {{\mathbf {u}}}_\lambda \cdot \nabla \mu _\lambda ^n\\&\quad +\frac{1}{2}\int _0^t \left\| \nabla P_nB(\varphi _\lambda ^n(s))\right\| ^2_{{\mathscr {L}}^2(K,H)}\,{\mathrm d}s +\sum _{j=0}^\infty \int _{Q_t} \Psi _\lambda ''(\varphi _\lambda ^n)|P_nB(\varphi _\lambda ^n)e_j|^2\\&\quad +\int _0^t\left( \mu _\lambda ^n(s), B(\varphi _\lambda ^n(s))\,{\mathrm d}W(s)\right) _H \end{aligned}$$

for every $t\in [0,T]$, ${\mathbb {P}}$-almost surely. We show now uniform estimates on the terms on the right-hand side, independent of both $\lambda$ and *n*. These will show a posteriori that $\varphi _\lambda$ and $\mu _\lambda$ are actually more regular. For this reason and for brevity of notation, we omit from now on the dependence on *n* and refer to (Scarpa 2018, 2020) for more detail.

To this end, noting that the definition of $\mu _\lambda$ and assumption **A1** imply

$$\begin{aligned} |(\mu _\lambda )_{\mathcal {O}}| =|(\Psi _\lambda '(\varphi _\lambda ))_{\mathcal {O}}| \le \left\| \Psi _\lambda '(\varphi _\lambda )\right\| _{L^1({\mathcal {O}})} \le c\left( 1 + \int _{\mathcal {O}}\Psi _\lambda (\varphi _\lambda )\right) \,, \end{aligned}$$

on the left-hand side, we get

$$\begin{aligned} \int _{\mathcal {O}}\Psi _\lambda (\varphi _\lambda (t)) \ge \frac{1}{c}|(\mu _\lambda (t))_{\mathcal {O}}| - c. \end{aligned}$$

On the right-hand side, thanks to the Hölder and Young inequalities, the inclusion $V_1\hookrightarrow L^6({\mathcal {O}})$, and the estimate (3.7), proceeding as in Sect. 3.1, we have

$$\begin{aligned} \int _{\mathcal {O}}\Psi _\lambda (\varphi _0) +\int _{Q_t}\varphi _\lambda {{\mathbf {u}}}_\lambda \cdot \nabla \mu _\lambda&\le \int _{\mathcal {O}}\Psi (\varphi _0) + \frac{1}{2}\int _{Q_t}|\nabla \mu _\lambda |^2\\&\quad +\frac{1}{2}\int _0^t\left\| \varphi _\lambda (s)\right\| ^2_{V_1}\left\| {{\mathbf {u}}}_\lambda (s)\right\| _U^2\,{\mathrm d}s\\&\le c+\frac{1}{2}\int _{Q_t}|\nabla \mu _\lambda |^2 +ct^{1-\frac{2}{p}}\left\| {{\mathbf {u}}}\right\| _{\mathcal {U}}^2\left\| \nabla \varphi \right\| _{L^\infty (0,t; H)}^2 \end{aligned}$$

Moreover, assumptions **A3** and **A1** yield, together with the Hölder inequality and (3.7),

$$\begin{aligned}&\frac{1}{2}\int _0^t \left\| \nabla B(\varphi _\lambda (s))\right\| ^2_{{\mathscr {L}}^2(K,H)}\,{\mathrm d}s +\sum _{j=0}^\infty \int _{Q_t} \Psi _\lambda ''(\varphi _\lambda )|B(\varphi _\lambda )e_j|^2\\&\quad \le c\left( 1+\int _0^t\left\| \varphi _\lambda (s)\right\| _{V_1}^2\,{\mathrm d}s\right) +\sum _{j=0}^\infty \int _0^t\left\| \Psi _\lambda ''(\varphi _\lambda (s))\right\| _{L^\gamma ({\mathcal {O}})} \left\| B(\varphi _\lambda (s))e_j\right\| _{L^{\frac{2\gamma }{\gamma -1}}({\mathcal {O}})}^2\,{\mathrm d}s\\&\quad \le c\left( 1 + \int _0^t\left\| \nabla \varphi _\lambda (s)\right\| _{H}^2\,{\mathrm d}s +\int _{Q_t}\Psi _\lambda (\varphi _\lambda )\right) \\&\quad \le c + ct\left\| \nabla \varphi _\lambda \right\| _{L^\infty (0,t; H)}^2+ ct\left\| \Psi _\lambda (\varphi _\lambda )\right\| _{L^\infty (0,t; L^1({\mathcal {O}}))}. \end{aligned}$$

Finally, the Burkholder–Davis–Gundy and the Poincaré–Wirtinger inequalities give, together with assumption **A3**,

$$\begin{aligned}&{{\mathbb {E}}}\sup _{r\in [0,t]}\left| \int _0^r\left( \mu _\lambda (s), B(\varphi _\lambda (s))\,{\mathrm d}W(s)\right) _H\right| ^{p/2}\\&\quad \le c{{\mathbb {E}}}\left( \int _0^t\left\| \mu _\lambda (s)\right\| _H^2 \left\| B(\varphi _\lambda (s))_H\right\| _{{\mathscr {L}}^2(K,H)}^2\,{\mathrm d}s\right) ^{p/4}\\&\quad \le c{{\mathbb {E}}}\left\| \mu _\lambda \right\| _{L^2(0,t; H)}^{p/2} \le \delta {{\mathbb {E}}}\left\| \nabla \mu _\lambda \right\| ^p_{L^2(0,t; H)} + c_\delta \left( 1 + {{\mathbb {E}}}\left\| (\mu _\lambda )_{\mathcal {O}}\right\| ^{p/2}_{L^2(0,t)}\right) \,, \end{aligned}$$

for every $\delta >0$, where we have updated the value of *c* and $c_\delta$ step-by-step, independently of $\lambda$. Putting all this information together, choosing $\delta$ sufficiently small, rearranging the terms, and updating again the value of *c*, we infer that

$$\begin{aligned}&{{\mathbb {E}}}\left\| \nabla \varphi _\lambda \right\| _{L^\infty (0,t; H)}^p +{{\mathbb {E}}}\left\| \Psi _\lambda (\varphi _\lambda )\right\| _{L^\infty (0,t;L^1({\mathcal {O}}))}^{p/2} +{{\mathbb {E}}}\left\| (\mu _\lambda )_{\mathcal {O}}\right\| _{L^\infty (0,t)}^{p/2} +{{\mathbb {E}}}\left\| \nabla \mu _\lambda \right\| ^{p}_{L^2(0,t; H)} \\&\quad \le c\left[ 1 + \left( t^{\frac{p}{2}-1}\left\| {{\mathbf {u}}}\right\| _{\mathcal {U}}^p+t^{\frac{p}{2}}\right) {{\mathbb {E}}}\left\| \nabla \varphi \right\| _{L^\infty (0,t; H)}^p\right. \\&\qquad \left. + t^{\frac{p}{2}}{{\mathbb {E}}}\left\| \Psi _\lambda (\varphi _\lambda )\right\| _{L^\infty (0,t; L^1({\mathcal {O}}))}^{p/2} +t^{p/4}{{\mathbb {E}}}\left\| (\mu _\lambda )_{\mathcal {O}}\right\| _{L^\infty (0,t)}^{p/2}\right] \qquad \forall \,t\in [0,T]. \end{aligned}$$

Consequently, we can close the estimate on a certain subinterval $[0,T_0]$, where $T_0$ is chosen sufficiently small in order to incorporate the terms on the right-hand side into the corresponding ones on the left. Also, a patching argument as in Sect. 3.1 allows then to extend the estimate to the whole interval [0, *T*], and we obtain

3.8 $$\begin{aligned}&\left\| \varphi _\lambda \right\| _{L^p(\Omega ; L^\infty (0,T; V_1))} +\left\| \mu _\lambda \right\| _{L^{p/2}_{\mathscr {P}}(\Omega ; L^2(0,T; V_1))} +\left\| \nabla \mu _\lambda \right\| _{L^{p}_{\mathscr {P}}(\Omega ; L^2(0,T; H))}\nonumber \\&\quad +\left\| \Psi _\lambda (\varphi _\lambda )\right\| _{L^{p/2}(\Omega ; L^\infty (0,T; L^1(\Omega )))} \le c\left( 1+\left\| {{\mathbf {u}}}\right\| _{{\mathcal {U}}}^{\frac{2p}{p-2}}\right) \,, \end{aligned}$$

which by comparison in $\mu _\lambda =-\Delta \varphi _\lambda + \Psi _\lambda '(\varphi _\lambda )$ and estimate (3.7) gives also

3.9 $$\begin{aligned} \left\| \Psi '_\lambda (\varphi _\lambda )\right\| _{L^{p/2}_{\mathscr {P}}(\Omega ; L^2(0,T; H))} \le c\left( 1+\left\| {{\mathbf {u}}}\right\| _{{\mathcal {U}}}^{\frac{2p}{p-2}}\right) . \end{aligned}$$

Finally, note that by assumption **A3** and the estimate (3.8), we have

$$\begin{aligned} \left\| B(\varphi _\lambda )\right\| _{L^\infty (\Omega \times (0,T); {\mathscr {L}}^2(K,H)) \cap L^p(\Omega ; L^\infty (0,T; {\mathscr {L}}^2(K,V_1)))}\le c\,, \end{aligned}$$

so that the classical result by Flandoli and Gatarek (1995, Lem. 2.1) ensures in particular that

3.10 $$\begin{aligned} \left\| I_\lambda := \int _0^\cdot B(\varphi _\lambda (s))\,{\mathrm d}W(s)\right\| _{L^p_{\mathscr {P}}(\Omega ; W^{s,p}(0,T; V_1))} \le c_{s} \qquad \forall \,s\in (0,1/2). \end{aligned}$$

Consequently, by comparison in (3.2), it is not difficult to check that

$$\begin{aligned} \left\| \varphi _\lambda \right\| _{L^p_{\mathscr {P}}(\Omega ; W^{1,2}(0,T; V_1^*) +W^{s,p}(0,T; V_1))} \le c_s \qquad \forall \,s\in (0,1/2). \end{aligned}$$

Now, recalling that $p>2$, for all arbitrary $s\in (0,1/2)$ we have that $s-\frac{1}{p}\le \frac{1}{2}$, so that the usual Sobolev embeddings ensure that

$$\begin{aligned} W^{1,2}(0,T; V_1^*)\hookrightarrow W^{s,p}(0,T; V_1^*) \quad \forall \,s\in (0,1/2)\,, \end{aligned}$$

and we deduce that

3.11 $$\begin{aligned} \left\| \varphi _\lambda \right\| _{L^p_{\mathscr {P}}(\Omega ; W^{s,p}(0,T; V_1^*))} \le c_s \qquad \forall \,s\in (0,1/2). \end{aligned}$$

### Passage to the Limit

From the estimates (3.7)–(3.9), there exists a pair $(\varphi ,\mu )$, with

$$\begin{aligned} \varphi \in L^p_w(\Omega ; L^\infty (0,T; V_1))\cap L^p_{\mathscr {P}}(\Omega ; L^2(0,T; V_2))\,, \qquad \mu \in L^{p/2}_{\mathscr {P}}(\Omega ; L^2(0,T; V_1)) \end{aligned}$$

such that, as $\lambda \searrow 0$, on a non-relabelled subsequence we have

$$\begin{aligned} \varphi _\lambda {\mathop {\rightharpoonup }\limits ^{*}}\varphi \qquad&\text {in } L^p_w(\Omega ; L^\infty (0,T; V_1)) \cap L^p_{\mathscr {P}}(\Omega ; L^2(0,T; V_2))\,,\\ \mu _\lambda \rightharpoonup \mu \qquad&\text {in } L^{p/2}_{\mathscr {P}}(\Omega ; L^2(0,T; V_1)). \end{aligned}$$

Now, since $p>2$, we can fix ${\bar{s}}\in (\frac{1}{p}, \frac{1}{2})$, so that ${\bar{s}} p>1$: with this choice, by the classical Aubin–Lions–Simon compactness results (Simon 1987, Cor. 5) we have

$$\begin{aligned}&L^\infty (0,T; V_1)\cap L^2(0,T; V_2)\cap W^{{\bar{s}},p}(0,T; V_1^*)\\&\quad \hookrightarrow C^0([0,T]; H)\cap L^2(0,T; V_1) \qquad \text {compactly}. \end{aligned}$$

Hence, setting ${\mathcal {B}}_n$ as the closed ball of radius *n* in $L^\infty (0,T; V_1)\cap L^2(0,T; V_2)\cap W^{{\bar{s}},p}(0,T; V_1^*)$, we have that ${\mathcal {B}}_n$ is compact in $C^0([0,T]; H)\cap L^2(0,T; V_1)$, for every $n\in {\mathbb {N}}$. Consequently, denoting by $\nu _\lambda$ the law of $\varphi _\lambda$ on $C^0([0,T]; H)\cap L^2(0,T; V_1)$ for brevity, the Markov inequality and the uniform estimates (3.7), (3.8), and (3.11) yield

$$\begin{aligned} \nu _\lambda ({\mathcal {B}}_n^c)&= {\mathbb {P}}\{\left\| \varphi _\lambda \right\| _{L^\infty (0,T; V_1) \cap L^2(0,T; V_2)\cap W^{{\bar{s}},p}(0,T; V_1^*)}>n\}\\&\le \frac{1}{n}{{\mathbb {E}}}\left\| \varphi _\lambda \right\| _{L^\infty (0,T; V_1) \cap L^2(0,T; V_2)\cap W^{{\bar{s}},p}(0,T; V_1^*)}\le \frac{c}{n}\,, \end{aligned}$$

from which

$$\begin{aligned} \lim _{n\rightarrow \infty }\sup _{\lambda >0}\nu _\lambda ({\mathcal {B}}_n^c)=0. \end{aligned}$$

By the Prokhorov theorem, this implies that

$$\begin{aligned} \text {the laws of } (\varphi _\lambda )_\lambda \text { are tight on } C^0([0,T]; H)\cap L^2(0,T; V_1). \end{aligned}$$

Similarly, estimate (3.10) ensures by the same argument that

$$\begin{aligned} \text {the laws of } (I_\lambda )_\lambda \text { are tight on } C^0([0,T]; H). \end{aligned}$$

Let us show now that, possibly on a further subsequence, we have also the strong convergence

3.12 $$\begin{aligned} \varphi _\lambda \rightarrow \varphi \qquad \text {in } C^0([0,T]; H)\cap L^2(0,T; V_1) \quad {\mathbb {P}}\text {-a.s.} \end{aligned}$$

To this end, we use the following lemma due to Gyöngy and Krylov (1996, Lem. 1.1), which characterises the convergence in probability in a Polish space.

#### Lemma 3.1

Let ${\mathcal {X}}$ be a Polish space and $(Z_n)_n$ be a sequence of ${\mathcal {X}}$-valued random variables. Then, $(Z_n)_n$ converges in probability if and only if for any pair of subsequences $(Z_{n_k})_k$ and $(Z_{n_j})_j$, there exists a joint sub-subsequence $(Z_{n_{k_\ell }}, Z_{n_{j_\ell }})_\ell$ converging in law to a probability measure $\nu$ on ${\mathcal {X}}\times {\mathcal {X}}$ such that $\nu (\{(z_1,z_2)\in {\mathcal {X}}\times {\mathcal {X}}: z_1=z_2\})=1$.

We apply this lemma to ${\mathcal {X}}=C^0([0,T]; H)\cap L^2(0,T; V_1)$ and $(\varphi _{\lambda })_\lambda$. Given two arbitrary subsequences $(\varphi _{\lambda _k})_k$ and $(\varphi _{\lambda _j})_j$, since the laws of the pairs $(\varphi _{\lambda _k}, \varphi _{\lambda _j})_{k,j}$ are tight on $(C^0([0,T]; H)\cap L^2(0,T; V_1))^2$, there is a joint subsequence $(\varphi _{\lambda _{k_i}}, \varphi _{\lambda _{j_i}})_i$ converging weakly to a probability measure $\nu$ on $(C^0([0,T]; H)\cap L^2(0,T; V_1))^2$. By the Skorokhod representation theorem (Ikeda and Watanabe 1989, Thm. 2.7) and (van der Vaart and Wellner 1996, Thm. 1.10.4, Add. 1.10.5), there exist a new probability space $(\Omega ', {\mathscr {F}}', {\mathbb {P}}')$ and measurable maps $\phi _i:(\Omega ', {\mathscr {F}}')\rightarrow (\Omega ,{\mathscr {F}})$, such that ${\mathbb {P}}'\circ \phi _i^{-1}={\mathbb {P}}$ for every $i\in {\mathbb {N}}$ and

$$\begin{aligned} (\varphi _{\lambda _{k_i}}', \varphi _{\lambda _{j_i}}') :=(\varphi _{\lambda _{k_i}}, \varphi _{\lambda _{j_i}})\circ \phi _i \rightarrow (\varphi '_1, \varphi '_2) \qquad&\text {in } (C^0([0,T]; H)\cap L^2(0,T; V_1))^2\,,\quad {\mathbb {P}}'\text {-a.s.}\,, \end{aligned}$$

for some measurable random variables

$$\begin{aligned} (\varphi '_1, \varphi '_2):(\Omega ', {\mathscr {F}}')\rightarrow (C^0([0,T]; H)\cap L^2(0,T; V_1))^2. \end{aligned}$$

Similarly, we have

$$\begin{aligned} ({{\mathbf {u}}}_{\lambda _{k_i}}',{{\mathbf {u}}}_{\lambda _{j_i}}')&:=({{\mathbf {u}}}_{\lambda _{k_i}}, {{\mathbf {u}}}_{\lambda _{j_i}})\circ \phi _i \rightarrow ({{\mathbf {u}}}_1', {{\mathbf {u}}}_2')&\text {in } L^p(0,T; U)^2\,, \quad {\mathbb {P}}'\text {-a.s.}\,,\\ (I_{\lambda _{k_i}}', I_{\lambda _{j_i}}')&:= (I_{\lambda _{k_i}}, I_{\lambda _{j_i}})\circ \phi _i\rightarrow (I_1', I_2')&\text {in } C^0([0,T]; H)^2\,, \quad {\mathbb {P}}'\text {-a.s.}\,,\\ W_i'&:=W\circ \phi _i \rightarrow W'&\text {in } C^0([0,T]; K)\,, \quad {\mathbb {P}}'\text {-a.s.} \end{aligned}$$

for some measurable random variables

$$\begin{aligned} ({{\mathbf {u}}}_1', {{\mathbf {u}}}_2'):(\Omega ', {\mathscr {F}}')\rightarrow L^p(0,T; U) \end{aligned}$$

and

$$\begin{aligned} (I_1', I_2'):(\Omega ', {\mathscr {F}}')\rightarrow C^0([0,T]; H)^2\,, \qquad W':(\Omega ', {\mathscr {F}}')\rightarrow C^0([0,T]; U). \end{aligned}$$

Now, since ${{\mathbf {u}}}_\lambda \rightarrow {{\mathbf {u}}}$ in $L^p(0,T; U)$ ${\mathbb {P}}$-almost surely on the whole sequence $\lambda$, for every arbitrary $f\in C^0({\mathbb {R}})\cap L^\infty ({\mathbb {R}})$ we have

$$\begin{aligned} {{{\mathbb {E}}}}'\left[ f\left( \left\| {{\mathbf {u}}}_1'-{{\mathbf {u}}}_2'\right\| _{L^p(0,T; U)}\right) \right]&= \lim _{i\rightarrow \infty }{{{\mathbb {E}}}}'\left[ f\left( \left\| {{\mathbf {u}}}_{\lambda _{k_i}}'-{{\mathbf {u}}}_{\lambda _{j_i}}'\right\| _{L^p(0,T; U)}\right) \right] \\&= \lim _{i\rightarrow \infty }{{\mathbb {E}}}\left[ f\left( \left\| {{\mathbf {u}}}_{\lambda _{k_i}}-{{\mathbf {u}}}_{\lambda _{j_i}}\right\| _{L^p(0,T; U)}\right) \right] =0\,, \end{aligned}$$

from which ${{\mathbf {u}}}_1'={{\mathbf {u}}}_2'$ ${\mathbb {P}}'$-almost surely due to the arbitrariness of *f*. Let us set then ${{\mathbf {u}}}':={{\mathbf {u}}}_1'={{\mathbf {u}}}_2'$ and $(\mu _{\lambda _{k_i}}',\mu _{\lambda _{j_i}}') :=(\mu _{\lambda _{k_i}}, \mu _{\lambda _{j_i}})\circ \phi _i$: since the maps $\phi _i$ preserve the laws, from the uniform estimates (3.7)–(3.9) we deduce also that

$$\begin{aligned} (\varphi _{\lambda _{k_i}}',\varphi _{\lambda _{j_i}}') \rightarrow (\varphi _1' , \varphi _2') \qquad&\text {in } L^\ell _{\mathscr {P}}(\Omega '; C^0([0,T]; H)\cap L^2(0,T; V_1))^2 \quad \forall \,\ell \in [1,p)\,,\\ (\varphi _{\lambda _{k_i}}',\varphi _{\lambda _{j_i}}') {\mathop {\rightharpoonup }\limits ^{*}}(\varphi _1' , \varphi _2') \qquad&\text {in } L^p_w(\Omega '; L^\infty (0,T; V_1))^2\cap L^p_{\mathscr {P}}(\Omega '; L^2(0,T; V_2))^2\,,\\ (\mu _{\lambda _{k_i}}', \mu _{\lambda _{j_i}}' ) \rightharpoonup (\mu _1', \mu _2') \qquad&\text {in } L^{p/2}_{\mathscr {P}}(\Omega '; L^2(0,T; V_1))^2\,,\\ ({{\mathbf {u}}}_{\lambda _{k_i}}', {{\mathbf {u}}}_{\lambda _{j_i}}' ) {\mathop {\rightharpoonup }\limits ^{*}}({{\mathbf {u}}}', {{\mathbf {u}}}') \qquad&\text {in } L^{\infty }_{\mathscr {P}}(\Omega '; L^p(0,T; U))^2\,, \end{aligned}$$

for some measurable random variables

$$\begin{aligned} (\mu '_1, \mu '_2):(\Omega ', {\mathscr {F}}')\rightarrow L^2(0,T; V_1)^2. \end{aligned}$$

Now, if we introduce the filtration $({\mathscr {F}}_{i,t}')_{t\in [0,T]}$ as:

$$\begin{aligned} {\mathscr {F}}_{i,t}':=\sigma \{\varphi _{\lambda _{k_i}}'(s), \varphi _{\lambda _{j_i}}'(s), \mu _{\lambda _{k_i}}'(s),\mu _{\lambda _{j_i}}'(s), {{\mathbf {u}}}_{\lambda _{k_i}}'(s), {{\mathbf {u}}}_{\lambda _{j_i}}'(s), W_i'(s), I_{\lambda _{k_i}}'(s), I_{\lambda _{j_i}}'(s): s\le t\}\,, \end{aligned}$$

using classical representation theorems for martingales (see Flandoli and Gatarek 1995 and Da Prato and Zabczyk 2014, § 8.4) we have that $W_i'$ is a cylindrical Wiener process on $(\Omega ', {\mathscr {F}}', ({\mathscr {F}}_t')_{t\in [0,T]}, {\mathbb {P}}')$ and

$$\begin{aligned} I_{\lambda _{k_i}}'=\int _0^\cdot B(\varphi _{\lambda _{k_i}}'(s))\,{\mathrm d}W_i'(s)\,, \qquad I_{\lambda _{j_i}}'=\int _0^\cdot B(\varphi _{\lambda _{j_i}}'(s))\,{\mathrm d}W_i'(s)\,, \end{aligned}$$

so that on the new probability space $(\Omega ', {\mathscr {F}}', {\mathbb {P}}')$ we have

$$\begin{aligned} {\mathrm d}\varphi _{\lambda _{k_i}}' -\Delta \mu _{\lambda _{k_i}}' \,{\mathrm d}t +{{\mathbf {u}}}'_{\lambda _{k_i}}\cdot \nabla \varphi _{\lambda _{k_i}}' \,{\mathrm d}t = B(\varphi _{\lambda _{k_i}}') \,{\mathrm d}W_i'\,, \qquad&\varphi _{\lambda _{k_i}}'(0)=\varphi _0\,,\\ {\mathrm d}\varphi _{\lambda _{j_i}}' -\Delta \mu _{\lambda _{j_i}}' \,{\mathrm d}t +{{\mathbf {u}}}'_{\lambda _{j_i}}\cdot \nabla \varphi _{\lambda _{j_i}}'\,{\mathrm d}t = B(\varphi _{\lambda _{j_i}}' )\,{\mathrm d}W_i'\,, \qquad&\varphi _{\lambda _{j_i}}'(0)=\varphi _0\,, \end{aligned}$$

where the equations are intended in the usual variational sense (3.6). Now, the strong convergences of $(\varphi _{\lambda _{k_i}}', \varphi _{\lambda _{j_i}}')_i$ imply, together with the Lipschitz-continuity of *B*, that

$$\begin{aligned} (B(\varphi _{\lambda _{k_i}}'), B(\varphi _{\lambda _{j_i}}')) \rightarrow (B(\varphi _1'), B(\varphi _2')) \qquad \text {in } L^\ell _{\mathscr {P}}(\Omega '; C^0([0,T]; {\mathscr {L}}^2(K,H)))^2 \quad \forall \,\ell \in [1,p). \end{aligned}$$

Introducing then the limiting filtration $({\mathscr {F}}_{t}')_{t\in [0,T]}$ as

$$\begin{aligned} {\mathscr {F}}_{t}':=\sigma \{\varphi _1'(s), \varphi _2'(s), \mu _1'(s), \mu _2'(s), {{\mathbf {u}}}'(s), W'(s), I_1'(s), I_2'(s): s\le t\}\,, \qquad t\in [0,T]\,, \end{aligned}$$

a classical argument based again on the martingale representation theorem (see Flandoli and Gatarek 1995 and Da Prato and Zabczyk 2014, § 8.4) yields the identification

$$\begin{aligned} I_1'=\int _0^\cdot B(\varphi _1'(s))\,{\mathrm d}W'(s)\,, \qquad I_2'=\int _0^\cdot B(\varphi _2'(s))\,{\mathrm d}W'(s). \end{aligned}$$

Moreover, the strong convergences of $(\varphi _{\lambda _{k_i}}', \varphi _{\lambda _{j_i}}')_i$ together with the uniform estimate (3.9) on the nonlinearities also give

$$\begin{aligned} (\Psi '_{\lambda _{k_i}}(\varphi _{\lambda _{k_i}}'), \Psi '_{\lambda _{j_i}}(\varphi _{\lambda _{j_i}}'))\rightharpoonup (\Psi '(\varphi _1'), \Psi '(\varphi _2')) \qquad \text {in } L^{p/2}_{\mathscr {P}}(\Omega '; L^2(0,T; H))^2. \end{aligned}$$

Putting all this information together, we deduce that $(\varphi _1', \varphi _2')$ solves the limit problem (1.1)–(1.4) in the sense of Theorem 2.1 on the new probability space $(\Omega ', {\mathscr {F}}', {\mathbb {P}}')$, namely

$$\begin{aligned} {\mathrm d}\varphi _1' -\Delta \mu _1' \,{\mathrm d}t +{{\mathbf {u}}}'\cdot \nabla \varphi _1' \,{\mathrm d}t = B(\varphi _1') \,{\mathrm d}W'\,, \qquad&\varphi _1'(0)=\varphi _0\,,\\ {\mathrm d}\varphi _2' -\Delta \mu _2' \,{\mathrm d}t +{{\mathbf {u}}}'\cdot \nabla \varphi _2'\,{\mathrm d}t = B(\varphi _2' )\,{\mathrm d}W'\,, \qquad&\varphi _2'(0)=\varphi _0. \end{aligned}$$

Since we have already proved uniqueness of solutions in Sect. 3.1, we deduce that

$$\begin{aligned} \nu (\{(z_1,z_2)\in {\mathcal {X}}^2: z_1=z_2\}) ={\mathbb {P}}'\left\{ \left\| \varphi _1'-\varphi _2'\right\| _{C^0([0,T]; H)\cap L^2(0,T; V_1)}=0\right\} =1. \end{aligned}$$

so that Lemma 3.1 ensures the strong convergence (3.12) also on the original probability space $(\Omega , {\mathscr {F}}, {\mathbb {P}})$. Proceeding now in exactly the same way on $(\Omega , {\mathscr {F}}, {\mathbb {P}})$ instead, it is a standard matter to show that $(\varphi , \mu )$ is the unique solution to the state system (1.1)–(1.4). Clearly, the global estimate (2.2) follows directly by the computations in Sect. 3.3 and assumption **A3**,

### Continuous Dependence

Here we conclude the proof of Theorem 2.1 by showing the continuous dependence estimates (2.3)–(2.4).

First of all, (2.3) is a consequence of the already proved (2.2) and Sect. 3.1. Now, let us focus on proving (2.4). To this end, we use the same notation of Sect. 3.1 and use Itô’s formula for the square of the *H*-norm instead, getting

$$\begin{aligned}&\frac{1}{2}\left\| \varphi (t)\right\| _H^2 + \int _{Q_t}|\Delta \varphi |^2 - \int _{Q_t}(\Psi '(\varphi _1)-\Psi '(\varphi _2))\Delta \varphi +\int _{Q_t}\left( {{\mathbf {u}}}\cdot \nabla \varphi _1 + {{\mathbf {u}}}_2\cdot \nabla \varphi \right) \varphi \\&\quad =\frac{1}{2}\int _0^t\left\| B(\varphi _1(s))-B(\varphi _2(s))\right\| ^2_{{\mathscr {L}}^2(K,H)}\,{\mathrm d}s +\int _0^t\left( \varphi (s),(B(\varphi _1(s))\right. \\&\qquad \left. -B(\varphi _2(s)))\,{\mathrm d}W(s)\right) _H. \end{aligned}$$

The third term on the left-hand side can be handled thanks to assumption **A1**, the Hölder and Young inequalities, and the embedding $V_1\hookrightarrow L^6({\mathcal {O}})$, as

$$\begin{aligned}&\int _{Q_t}(\Psi '(\varphi _1)-\Psi '(\varphi _2))\Delta \varphi \\&\quad \le \frac{1}{2}\int _{Q_t}|\Delta \varphi |^2+ c\int _{Q_t}\left( 1+|\varphi _1|^4 + |\varphi _2|^4\right) |\varphi |^2\\&\quad \le \frac{1}{2}\int _{Q_t}|\Delta \varphi |^2+ \int _0^t\left( 1+\left\| \varphi _1(s)\right\| ^4_{L^6(D)} + \left\| \varphi _2(s)\right\| ^4_{L^6(D)}\right) \left\| \varphi (s)\right\| ^2_{L^6(D)}\,{\mathrm d}s\\&\quad \le \frac{1}{2}\int _{Q_t}|\Delta \varphi |^2+ \left( 1+\left\| \varphi _1\right\| ^4_{L^\infty (0,T; V_1)} + \left\| \varphi _2\right\| ^4_{L^\infty (0,T; V_1)}\right) \left\| \varphi \right\| _{L^2(0,T; V_1)}^2. \end{aligned}$$

The convection terms on the right-hand side can be treated similarly using the divergence theorem, the Hölder and Young inequalities, and the inclusion $L^6(\Omega )\hookrightarrow V_1$ as

$$\begin{aligned}&\int _{Q_t}\left( {{\mathbf {u}}}\cdot \nabla \varphi _1 + {{\mathbf {u}}}_2\cdot \nabla \varphi \right) \varphi \\&\quad = \int _{Q_t}{{\mathbf {u}}}\cdot \nabla \varphi _1\varphi \le \left\| \varphi \right\| _{L^2(0,T; V_1)}^2+ c\left\| \varphi _1\right\| _{L^\infty (0,T; V_1)}^2\left\| {{\mathbf {u}}}\right\| _{L^2(0,T; U)}^2. \end{aligned}$$

Hence, we rearrange the terms and take power *p*/6 at both sides, obtaining, thanks to the Hölder and Young inequalities,

$$\begin{aligned}&{{\mathbb {E}}}\left\| \varphi \right\| _{L^\infty (0,T;H)}^{p/3} + {{\mathbb {E}}}\left\| \Delta \varphi \right\| _{L^2(0,T; H)}^{p/3}\\&\quad \le c\left[ 1 + \left\| \varphi _1\right\| _{L^p(\Omega ; L^\infty (0,T; V_1))}^{\frac{2}{3}p} +\left\| \varphi _2\right\| _{L^p(\Omega ; L^\infty (0,T; V_1))}^{\frac{2}{3}p}\right] \left\| \varphi \right\| _{L^p_{\mathscr {P}}(\Omega ;L^2(0,T; V_1))}^{p/3}\\&\qquad +c{{\mathbb {E}}}\left\| \varphi \right\| _{L^2(0,T; V_1)}^{p/3}\\&\qquad +c\left\| \varphi _1\right\| _{L^p(\Omega ; L^\infty (0,T; V_1))}^{p/3}\left\| {{\mathbf {u}}}\right\| _{{\mathcal {U}}}^{p/3} \\&\qquad +c{{\mathbb {E}}}\sup _{t\in [0,T]}\left| \int _0^t\left( \varphi (s),(B(\varphi _1(s))-B(\varphi _2(s)))\,{\mathrm d}W(s)\right) _H\right| ^{p/6} \end{aligned}$$

where the Burkholder–Davis–Gundy inequality and the Lipschitz-continuity of *B* yield

$$\begin{aligned}&{{\mathbb {E}}}\sup _{t\in [0,T]}\left| \int _0^t\left( \varphi (s),(B(\varphi _1(s))-B(\varphi _2(s)))\,{\mathrm d}W(s)\right) _H\right| ^{p/6}\\&\quad \le \sigma {{\mathbb {E}}}\left\| \varphi \right\| _{L^\infty (0,T; H)}^{p/3} +c_\sigma {{\mathbb {E}}}\left\| \varphi \right\| _{L^2(0,T; H)}^{p/3} \end{aligned}$$

for all $\sigma >0$. Hence, choosing $\sigma$ sufficiently small and rearranging the terms, the continuous dependence (2.4) follows from the already proved estimates (2.2)–(2.3). This concludes the proof of Theorem 2.1.

## Existence of Optimal Controls

In this section, we prove Theorem 2.4 showing that the optimisation problem **(CP)** always admits a relaxed optimal control ${{\mathbf {u}}}\in {\mathcal {U}}_{ad}$ and a deterministic optimal control ${{\mathbf {u}}}^{det}\in {\mathcal {U}}_{ad}^{det}$. The main idea is to use the direct method from calculus of variations, combined with a stochastic compactness argument.

Let $({{\mathbf {u}}}_n)_n\subset {\mathcal {U}}_{ad}$ be a minimising sequence for the functional ${\widetilde{J}}$, in the sense that

$$\begin{aligned} {\tilde{J}}({{\mathbf {u}}}_n)\searrow \inf _{{{\mathbf {v}}}\in {\mathcal {U}}_{ad}}{\widetilde{J}}({{\mathbf {v}}})\,, \end{aligned}$$

and define $(\varphi _n,\mu _n)_n$ as the unique respective solutions to the state system (1.1)–(1.4), in the sense of Theorem 2.1. Thanks to the definition of ${\mathcal {U}}_{ad}$ and the estimate (2.2), we deduce that there exist ${{\mathbf {u}}}\in {\mathcal {U}}_{ad}$ and a triplet $(\varphi ,\mu ,\xi )$ with

$$\begin{aligned}&\varphi \in L^p_{\mathscr {P}}(\Omega ; C^0([0,T]; H)\cap L^2(0,T; V_2))\cap L^p_w(\Omega ; L^\infty (0,T; V_1))\,,\\&\mu \in L^{p/2}_{\mathscr {P}}(\Omega ; L^2(0,T; V_1))\,, \qquad \xi \in L^{p/2}_{\mathscr {P}}(\Omega ; L^2(0,T; H))\,, \end{aligned}$$

such that, as $n\rightarrow \infty$, possibly on a subsequence,

$$\begin{aligned} \varphi _n{\mathop {\rightharpoonup }\limits ^{*}}\varphi \qquad&\text {in } L^p_w(\Omega ; L^\infty (0,T; V_1))\cap L^p_{\mathscr {P}}(\Omega ; L^2(0,T; V_2))\,,\\ \mu _n\rightharpoonup \mu \qquad&\text {in } L^{p/2}_{\mathscr {P}}(\Omega ; L^2(0,T; V_1))\,,\\ \Psi '(\varphi _n)\rightharpoonup \xi \qquad&\text {in } L^{p/2}_{\mathscr {P}}(\Omega ; L^2(0,T; H))\,,\\ {{\mathbf {u}}}_n{\mathop {\rightharpoonup }\limits ^{*}}{{\mathbf {u}}}\qquad&\text {in } L^{\infty }_{\mathscr {P}}(\Omega ; L^p(0,T; U)). \end{aligned}$$

Assumption **A3** and the uniform estimates on $(\varphi _n)_n$ ensure also that

$$\begin{aligned} \left\| B(\varphi _n)\right\| _{L^\infty (\Omega \times (0,T); {\mathscr {L}}^2(K,H)) \cap L^p(\Omega ; L^\infty (0,T; {\mathscr {L}}^2(K,V_1)))} \le c\,, \end{aligned}$$

so that in particular

$$\begin{aligned} \left\| I_n:=\int _0^\cdot B(\varphi _n(s))\,{\mathrm d}W(s)\right\| _{L^p_{\mathscr {P}}(\Omega ; W^{s,p}(0,T; V_1))} \le c_s \quad \forall \,s\in (0,1/2). \end{aligned}$$

By comparison in the equation (1.1), we infer then

$$\begin{aligned} \left\| \varphi _n\right\| _{L^p_{\mathscr {P}}(\Omega ; W^{s,p}(0,T; V_1^*))} \le c_s \quad \forall \,s\in (0,1/2)\,, \end{aligned}$$

which ensures that the laws of $(\varphi _n)_n$ are tight on the space $C^0([0,T]; H)\cap L^2(0,T; V_1)$. We argue now on the same line of Sect. 3.4. As a consequence of the Skorokhod theorem, there is a probability space $(\Omega ', {\mathscr {F}}', {\mathbb {P}}')$ and measurable maps $\phi _i:(\Omega ', {\mathscr {F}}')\rightarrow (\Omega , {\mathscr {F}})$ with ${\mathbb {P}}'\circ \phi _i^{-1}={\mathbb {P}}$ for all $i\in {\mathbb {N}}$, such that

$$\begin{aligned} \varphi _{n_i}':=\varphi _{n_i}\circ \phi _i \rightarrow \varphi ' \qquad&\text {in } L^\ell _{\mathscr {P}}(\Omega '; C^0([0,T]; H)\cap L^2(0,T; V_1)) \quad \forall \,\ell \in [1,p)\,,\\ \varphi _{n_i}' {\mathop {\rightharpoonup }\limits ^{*}}\varphi ' \qquad&\text {in } L^p_w(\Omega '; L^\infty (0,T; V_1))\cap L^p_{\mathscr {P}}(\Omega '; L^2(0,T; V_2))\,,\\ \mu _{n_i}' :=\mu _{n_i}\circ \phi _i\rightharpoonup \mu ' \qquad&\text {in } L^{p/2}_{\mathscr {P}}(\Omega '; L^2(0,T; V_1))\,,\\ {{\mathbf {u}}}_{n_i}':={{\mathbf {u}}}_{n_i}\circ \phi _i{\mathop {\rightharpoonup }\limits ^{*}}{{\mathbf {u}}}' \qquad&\text {in } L^{\infty }_{\mathscr {P}}(\Omega '; L^p(0,T; U))\,,\\ \varphi '_{Q,i}:=\varphi _Q\circ \phi _i'\rightharpoonup \varphi _Q' \qquad&\text {in } L^2_{\mathscr {P}}(\Omega '; L^2(0,T; H))\,,\\ \varphi _{T,i}':=\varphi _T\circ \phi _i \rightharpoonup \varphi _T' \qquad&\text {in } L^2(\Omega ',{\mathscr {F}}_T'; H). \end{aligned}$$

Furthermore, on the new probability space we have

$$\begin{aligned} {\mathrm d}\varphi _{n_i}' -\Delta \mu _{n_i}' \,{\mathrm d}t +{{\mathbf {u}}}_{n_i}'\cdot \nabla \varphi _{n_i}' \,{\mathrm d}t = B(\varphi _{n_i}') \,{\mathrm d}W_i'\,, \qquad&\varphi _{n_i}'(0)=\varphi _0\,, \end{aligned}$$

where the stochastic integral is intended with respect to a suitably defined filtration $({\mathscr {F}}_{i,t})_{t\in [0,T]}$. Proceeding as in Sect. 3.4, we infer that

$$\begin{aligned} \Psi '(\varphi _{n_i}')\rightharpoonup \Psi '(\varphi ') \qquad \text {in } L^{p/2}_{\mathscr {P}}(\Omega '; L^2(0,T; H))\,, \end{aligned}$$

so that by assumption **A3** and the martingale representation theorem we can pass to the limit as $i\rightarrow \infty$ on the new probability space and get

$$\begin{aligned} {\mathrm d}\varphi ' -\Delta \mu ' \,{\mathrm d}t +{{\mathbf {u}}}'\cdot \nabla \varphi ' \,{\mathrm d}t = B(\varphi ') \,{\mathrm d}W'\,, \qquad&\varphi '(0)=\varphi _0. \end{aligned}$$

This shows that ${{\mathbf {u}}}'\in {\mathcal {U}}_{ad}'$ and that $(\varphi ',\mu ')=S'({{\mathbf {u}}}')$. To conclude that ${{\mathbf {u}}}'$ is a relaxed optimal control for the optimisation problem **(CP)**, we note that by the weak lower semicontinuity of the cost functional *J* we have

$$\begin{aligned} {\widetilde{J}}'({{\mathbf {u}}}')&= \frac{\alpha _1}{2}{{{\mathbb {E}}}}'\int _Q|\varphi '-\varphi '_Q|^2 + \frac{\alpha _2}{2}{{{\mathbb {E}}}}'\int _{\mathcal {O}}|\varphi '(T)-\varphi '_T|^2 + \frac{\alpha _3}{2}{{\mathbb {E}}}\int _Q|{{\mathbf {u}}}'|^2\\&\le \liminf _{i\rightarrow \infty } \left( \frac{\alpha _1}{2}{{{\mathbb {E}}}}'\int _Q|\varphi _{n_i}'-\varphi _{Q,i}'|^2 + \frac{\alpha _2}{2}{{{\mathbb {E}}}}'\int _{\mathcal {O}}|\varphi _{n_i}'(T)-\varphi _{T,i}'|^2 + \frac{\alpha _3}{2}{{{\mathbb {E}}}}'\int _Q|{{\mathbf {u}}}_{n_i}'|^2\right) \\&=\liminf _{i\rightarrow \infty } \left( \frac{\alpha _1}{2}{{\mathbb {E}}}\int _Q|\varphi _{n_i}-\varphi _Q|^2 + \frac{\alpha _2}{2}{{\mathbb {E}}}\int _{\mathcal {O}}|\varphi _{n_i}(T)-\varphi _T|^2 + \frac{\alpha _3}{2}{{\mathbb {E}}}\int _Q|{{\mathbf {u}}}_{n_i}|^2\right) \\&=\liminf _{n\rightarrow \infty }{\widetilde{J}}({{\mathbf {u}}}_n)= \inf _{{{\mathbf {v}}}\in {\mathcal {U}}_{ad}}{\tilde{J}}({{\mathbf {v}}})\,, \end{aligned}$$

so that ${{\mathbf {u}}}'\in {\mathcal {U}}'_{ad}$ is a relaxed optimal control in the sense of Definition 2.3.

In order to show existence of a deterministic optimal control, the argument is similar. We start taking a minimising sequence $({{\mathbf {u}}}_n)_n\subset {\mathcal {U}}_{ad}^{det}$ such that

$$\begin{aligned} {\tilde{J}}({{\mathbf {u}}}_n)\searrow \inf _{{{\mathbf {v}}}\in {\mathcal {U}}^{det}_{ad}}{\widetilde{J}}({{\mathbf {v}}}). \end{aligned}$$

Arguing exactly as above, thanks to the fact that $({{\mathbf {u}}}_n)_n$ are deterministic, in this case we have that ${{\mathbf {u}}}_{n_i}'={{\mathbf {u}}}_{n_i}$ for every $i\in {\mathbb {N}}$. Consequently, in this case we can $(\varphi _n)_n$ inherits some strong compactness properties on the original probability space, using a similar argument to the one of Sect. 3.4, by employing Lemma 3.12. Namely, we infer the strong convergence

$$\begin{aligned} \varphi _n\rightarrow \varphi \qquad \text {in } C^0([0,T];H)\cap L^2(0,T; V_1)\,, \quad {\mathbb {P}}\text {-a.s.} \end{aligned}$$

on the original probability space $(\Omega ,{\mathscr {F}},{\mathbb {P}})$. It follows then that $\xi =\Psi '(\varphi )$ almost everywhere, and letting $n\rightarrow \infty$ yields

$$\begin{aligned} {\mathrm d}\varphi -\Delta \mu \,{\mathrm d}t +{{\mathbf {u}}}\cdot \nabla \varphi \,{\mathrm d}t = B(\varphi ) \,{\mathrm d}W\,, \qquad \varphi (0)=\varphi _0\,, \end{aligned}$$

so that $(\varphi ,\mu )=S({{\mathbf {u}}})$. At this point, the conclusion follows as above by lower semicontinuity of the cost functional.

## Linearised System and Differentiability of the Control-to-State Map

The aim of this section is to prove that the linearised state system (2.6)–(2.7) is well posed and to characterise its solution as the derivative on the control-to-state map. Namely, we prove here Theorem 2.5.

### Existence

Let ${{\mathbf {u}}}\in {\widetilde{{\mathcal {U}}}}_{ad}$ and ${{\mathbf {h}}}\in {\mathcal {U}}$ be arbitrary and fixed. Using the notation of Sect. 3.2, we consider the approximated linearised problem

5.1 $$\begin{aligned} {\mathrm d}\theta _{{{\mathbf {h}}},\lambda } - \Delta \nu _{{{\mathbf {h}}},\lambda }\,{\mathrm d}t +{{\mathbf {h}}}\cdot \nabla \varphi \,{\mathrm d}t + {{\mathbf {u}}}_\lambda \cdot \nabla \theta _{{{\mathbf {h}}},\lambda }\,{\mathrm d}t = DB(\varphi )\theta _{{{\mathbf {h}}},\lambda }\,{\mathrm d}W \qquad&\text {in } (0,T)\times {\mathcal {O}}\,, \end{aligned}$$

5.2 $$\begin{aligned} \nu _{{{\mathbf {h}}},\lambda }=-\Delta \theta _{{{\mathbf {h}}},\lambda } + \Psi _\lambda ''(\varphi )\theta _{{{\mathbf {h}}},\lambda } \qquad&\text {in } (0,T)\times {\mathcal {O}}\,, \end{aligned}$$

5.3 $$\begin{aligned} \mathbf{n}\cdot \nabla \theta _{{{\mathbf {h}}},\lambda } = \mathbf{n}\cdot \nabla \nu _{{{\mathbf {h}}},\lambda } = 0 \qquad&\text {in } (0,T)\times \partial {\mathcal {O}}\,, \end{aligned}$$

5.4 $$\begin{aligned} \theta _{{{\mathbf {h}}},\lambda }(0)=0 \qquad&\text {in } {\mathcal {O}}. \end{aligned}$$

Noting that $\Psi ''_\lambda (\varphi )\in L^\infty (\Omega \times Q)$, the classical variational approach ensures existence and uniqueness of the approximated solution

$$\begin{aligned}&\theta _{{{\mathbf {h}}},\lambda } \in L^2_{{\mathscr {P}}}\left( \Omega ; C^0([0,T]; H)\cap L^2(0,T; V_2)\right) \,,\\&\nu _{{{\mathbf {h}}},\lambda }=-\Delta \theta _{{{\mathbf {h}}},\lambda } +\Psi ''_\lambda (\varphi )\theta _{{{\mathbf {h}}},\lambda }\in L^2_{{\mathscr {P}}}(\Omega ; L^2(0,T; H))\,, \end{aligned}$$

in the sense that, for every $\zeta \in V_2$, for every $t\in [0,T]$, ${\mathbb {P}}$-almost surely,

5.5 $$\begin{aligned}&\left( \theta _{{{\mathbf {h}}},\lambda }(t),\zeta \right) _H - \int _{Q_t}\nu _{{{\mathbf {h}}},\lambda }\Delta \zeta -\int _{Q_t}(\varphi {{\mathbf {h}}}+ \theta _{{{\mathbf {h}}},\lambda }{{\mathbf {u}}})\cdot \nabla \zeta = \left( \int _0^tDB(\varphi )\theta _{{{\mathbf {h}}},\lambda }\,{\mathrm d}W(s), \zeta \right) _H. \end{aligned}$$

Noting that $(\theta _{{{\mathbf {h}}},\lambda })_{\mathcal {O}}=0$, we can write Itô’s formula for $\frac{1}{2}\left\| \nabla {\mathcal {N}}\theta _{{{\mathbf {h}}},\lambda }\right\| _H^2$, getting

$$\begin{aligned}&\frac{1}{2}\left\| \nabla {\mathcal {N}}\theta _{{{\mathbf {h}}},\lambda }(t)\right\| _H^2 +\int _{Q_t}|\nabla \theta _{{{\mathbf {h}}},\lambda }|^2\\&\quad =-\int _{Q_t}\Psi _\lambda ''(\varphi )|\theta _{{{\mathbf {h}}},\lambda }|^2 +\int _{Q_t}\left( \varphi {{\mathbf {h}}}+\theta _{{{\mathbf {h}}},\lambda }{{\mathbf {u}}}_\lambda \right) \cdot \nabla {\mathcal {N}}\theta _{{{\mathbf {h}}},\lambda }\\&\qquad +\frac{1}{2}\int _0^t\left\| \nabla {\mathcal {N}}DB(\varphi (s))\theta _{{\mathbf {h}}}(s)\right\| _{{\mathscr {L}}^2(K,H)}^2\,{\mathrm d}s\\&\qquad +\int _0^t\left( {\mathcal {N}}\theta _{{{\mathbf {h}}},\lambda }(s), DB(\varphi (s))\theta _{{{\mathbf {h}}},\lambda }(s)\,{\mathrm d}W(s)\right) _H. \end{aligned}$$

Now, assumption **A1**, the Hölder–Young inequalities and the compactness inequality (2.1), and the embedding $V_1\hookrightarrow L^6({\mathcal {O}})$ give, for all $\varepsilon >0$,

$$\begin{aligned}&-\int _{Q_t}\Psi _\lambda ''(\varphi )|\theta _{{{\mathbf {h}}},\lambda }|^2 +\int _{Q_t}\left( \varphi {{\mathbf {h}}}+\theta _{{{\mathbf {h}}},\lambda }{{\mathbf {u}}}_\lambda \right) \cdot \nabla {\mathcal {N}}\theta _{{{\mathbf {h}}},\lambda }\\&\quad \le \varepsilon \int _{Q_t}|\nabla \theta _{{{\mathbf {h}}},\lambda }|^2+ \left\| \varphi \right\| _{L^\infty (0,T; V_1)}^2\\&\qquad + c_\varepsilon \int _0^t\left( 1+\left\| {{\mathbf {h}}}(s)\right\| _U^2+\left\| {{\mathbf {u}}}_\lambda (s)\right\| _U^2\right) \left\| \nabla {\mathcal {N}}\theta _{{{\mathbf {h}}},\lambda }(s)\right\| _H^2\,{\mathrm d}s. \end{aligned}$$

Similarly, by **C2** and again the compactness inequality (2.1), we have

$$\begin{aligned} \frac{1}{2}\int _0^t\left\| \nabla {\mathcal {N}}DB(\varphi (s))\theta _{{\mathbf {h}}}(s)\right\| _{{\mathscr {L}}^2(K,H)}^2\,{\mathrm d}s\le \varepsilon \int _{Q_t}|\nabla \theta _{{{\mathbf {h}}},\lambda }|^2 + c_\varepsilon \int _0^t \left\| \nabla {\mathcal {N}}\theta _{{{\mathbf {h}}},\lambda }(s)\right\| _H^2\,{\mathrm d}s. \end{aligned}$$

As for the stochastic integral, the Burkholder–Davis–Gundy and Young inequalities give (see, for example, Marinelli and Scarpa 2020, Lem. 4.1), together with (2.1) and **C2**

$$\begin{aligned}&{{\mathbb {E}}}\sup _{r\in [0,t]}\left| \int _0^r\left( {\mathcal {N}}\theta _{{{\mathbf {h}}},\lambda }(s), DB(\varphi (s))\theta _{{{\mathbf {h}}},\lambda }(s)\,{\mathrm d}W(s)\right) _H\right| ^{p/2}\\&\quad \le \varepsilon {{\mathbb {E}}}\left\| {\mathcal {N}}\theta _{{{\mathbf {h}}},\lambda }\right\| _{L^\infty (0,t; H)}^p +c_\varepsilon {{\mathbb {E}}}\left\| \theta _{{{\mathbf {h}}},\lambda }\right\| _{L^2(0,t; H)}^p\\&\quad \le \varepsilon {{\mathbb {E}}}\left\| \nabla {\mathcal {N}}\theta _{{{\mathbf {h}}},\lambda }\right\| _{L^\infty (0,t; H)}^p+ \varepsilon {{\mathbb {E}}}\left\| \nabla \theta _{{{\mathbf {h}}},\lambda }\right\| _{L^\infty (0,t; H)}^p +c_\varepsilon {{\mathbb {E}}}\left\| \nabla {\mathcal {N}}\theta _{{{\mathbf {h}}},\lambda }\right\| _{L^2(0,t; H)}^p. \end{aligned}$$

Consequently, using the same iterative-patching argument of Sect. 3.1, raising to power *p*/2, taking supremum in time and expectations, we infer that

5.6 $$\begin{aligned} \left\| \theta _{{{\mathbf {h}}},\lambda }\right\| _{L^p_{\mathscr {P}}(\Omega ; C^0([0,T]; V_1^*)\cap L^2(0,T; V_1))} \le c. \end{aligned}$$

Now, Itô’s formula for $\frac{1}{2}\left\| \theta _{{{\mathbf {h}}},\lambda }\right\| _H^2$ yields

$$\begin{aligned}&\frac{1}{2}\left\| \theta _{{{\mathbf {h}}},\lambda }\right\| _H^2 +\int _{Q_t}|\Delta \theta _{{{\mathbf {h}}},\lambda }|^2 =\int _{Q_t}\left( \varphi {{\mathbf {h}}}+\theta _{{{\mathbf {h}}},\lambda }{{\mathbf {u}}}_\lambda \right) \cdot \nabla \theta _{{{\mathbf {h}}},\lambda } +\int _{Q_t}\Psi ''_\lambda (\varphi )\theta _{{{\mathbf {h}}},\lambda }\Delta \theta _{{{\mathbf {h}}},\lambda }\\&\quad +\frac{1}{2}\int _0^t \left\| DB(\varphi (s))\theta _{{{\mathbf {h}}},\lambda }(s)\right\| _{{\mathscr {L}}^2(K,H)}^2\,{\mathrm d}s +\int _0^t\left( \theta _{{{\mathbf {h}}},\lambda }(s), DB(\varphi (s))\theta _{{{\mathbf {h}}},\lambda }(s)\,{\mathrm d}W(s)\right) _H\,, \end{aligned}$$

where by the divergence theorem we have

$$\begin{aligned} \int _{Q_t}\left( \varphi {{\mathbf {h}}}+\theta _{{{\mathbf {h}}},\lambda }{{\mathbf {u}}}_\lambda \right) \cdot \nabla \theta _{{{\mathbf {h}}},\lambda } = \int _{Q_t}\varphi {{\mathbf {h}}}\cdot \nabla \theta _{{{\mathbf {h}}},\lambda }. \end{aligned}$$

Hence, it is not difficult to see that, using again the Hölder, Young and Burkholder–Davis–Gundy inequalities, assumption **C2**, and the estimate (5.6), all the terms on the right-hand side can be handled, except the one containing $\Psi ''$. For this one, we proceed using **C1**, the embedding $V_1\hookrightarrow L^6({\mathcal {O}})$, as

$$\begin{aligned} \int _{Q_t}\Psi ''_\lambda (\varphi )\theta _{{{\mathbf {h}}},\lambda }\Delta \theta _{{{\mathbf {h}}},\lambda }&\le \varepsilon \int _{Q_t}|\Delta \theta _{{{\mathbf {h}}},\lambda }|^2 +c_\varepsilon \int _0^1\left( 1 + \left\| \varphi (s)\right\| _{V_1}^4\right) \left\| \theta _{{{\mathbf {h}}},\lambda }(s)\right\| _{V_1}^2\,{\mathrm d}s\\&\le \varepsilon \int _{Q_t}|\Delta \theta _{{{\mathbf {h}}},\lambda }|^2 +c_\varepsilon \left( 1 + \left\| \varphi \right\| _{L^\infty (0,T; V_1)}^4\right) \left\| \theta _{{{\mathbf {h}}},\lambda }\right\| _{L^2(0,T; V_1)}^2\,, \end{aligned}$$

where, thanks to (5.6) and the Hölder inequality,

$$\begin{aligned}&\left\| \left\| \varphi \right\| _{L^\infty (0,T; V_1)}^4 \left\| \theta _{{{\mathbf {h}}},\lambda }\right\| _{L^2(0,T; V_1)}^2\right\| _{L^{p/6}(\Omega )}\\&\quad \le \left\| \varphi \right\| _{L^p(\Omega ; L^\infty (0,T; V_1))}^4 \left\| \theta _{{{\mathbf {h}}},\lambda }\right\| _{L^p_{\mathscr {P}}(\Omega ; L^2(0,T; V_1))}^2\le c. \end{aligned}$$

Consequently, we deduce that

5.7 $$\begin{aligned} \left\| \theta _{{{\mathbf {h}}},\lambda }\right\| _{L^{p/3}_{\mathscr {P}}(\Omega ; C^0([0,T]; H)\cap L^2(0,T; V_2))} \le c\,, \end{aligned}$$

from which, by comparison in (5.2),

5.8 $$\begin{aligned} \left\| \nu _{{{\mathbf {h}}},\lambda }\right\| _{L^{p/3}_{\mathscr {P}}(\Omega ; L^2(0,T; H))} \le c\,, \end{aligned}$$

We infer the existence of $(\theta _{{\mathbf {h}}}, \nu _{{\mathbf {h}}})$ with

$$\begin{aligned}&\theta _{{\mathbf {h}}}\in L^{p}_{{\mathscr {P}}}\left( \Omega ; C^0([0,T]; V_1^*)\cap L^2(0,T; V_1)\right) \cap L^{p/3}_w(\Omega ; L^\infty (0,T; H))\\&\quad \cap L^{p/3}_{\mathscr {P}}(\Omega ; L^2(0,T; V_2))\,,\\&\nu _{{\mathbf {h}}}\in L^{p/3}_{{\mathscr {P}}}(\Omega ; L^2(0,T; H))\,, \end{aligned}$$

such that, as $\lambda \searrow 0$ (possibly on a subsequence),

5.9 $$\begin{aligned} \theta _{{{\mathbf {h}}},\lambda }{\mathop {\rightharpoonup }\limits ^{*}}\theta _{{\mathbf {h}}}\qquad&\text {in } L^{p}_{w}\left( \Omega ; L^\infty (0,T; V_1^*)\right) \cap L^{p/3}_{w}\left( \Omega ; L^\infty (0,T; H)\right) \,, \end{aligned}$$

5.10 $$\begin{aligned} \theta _{{{\mathbf {h}}},\lambda }\rightharpoonup \theta _{{\mathbf {h}}}\qquad&\text {in } L^{p}_{\mathscr {P}}\left( \Omega ; L^2(0,T; V_1)\right) \cap L^{p/3}_{\mathscr {P}}\left( \Omega ; L^2(0,T; V_2)\right) \,, \end{aligned}$$

5.11 $$\begin{aligned} \nu _{{{\mathbf {h}}},\lambda }\rightharpoonup \nu _{{\mathbf {h}}}\qquad&\text {in } L^{p/3}_{\mathscr {P}}\left( \Omega ; L^2(0,T; H)\right) . \end{aligned}$$

Since the systems (5.1)–(5.4) and (2.6)–(2.9) are linear, the passage to the limit is straightforward. Indeed, by assumption **C2** and the dominated convergence theorem, it follows that

$$\begin{aligned} DB(\varphi )\theta _{{{\mathbf {h}}},\lambda }\rightharpoonup DB(\varphi )\theta _{{\mathbf {h}}}\qquad \text {in } L^{p}_{\mathscr {P}}\left( \Omega ; L^2(0,T; {\mathscr {L}}^2(K,H))\right) . \end{aligned}$$

Moreover, thanks to **C1** and the regularity of $\varphi$, we have $\Psi ''(\varphi ) \in L^3(\Omega ; L^\infty (0,T; L^3({\mathcal {O}})))$, so in particular

$$\begin{aligned} \Psi ''_\lambda (\varphi ) \rightarrow \Psi ''(\varphi ) \qquad \text {in } L^3(\Omega \times Q)\,, \end{aligned}$$

and also, thanks to (5.10),

$$\begin{aligned} \Psi ''_\lambda (\varphi )\theta _{{{\mathbf {h}}},\lambda } \rightharpoonup \Psi ''(\varphi )\theta _{{\mathbf {h}}}\qquad \text {in } L^{6/5}_{\mathscr {P}}(\Omega ; L^{6/5}(0,T; L^{6/5}({\mathcal {O}}))). \end{aligned}$$

We deduce that letting $\lambda \searrow 0$ in (5.5) we get that $(\theta _{{\mathbf {h}}}, \nu _{{\mathbf {h}}})$ is a solution to (2.6)–(2.9) in the sense of Theorem 2.5. The strong continuity in *H* of $\theta _{{\mathbf {h}}}$ follows a posteriori with a classical method by Itô’s formula on the limit equation (2.6).

### Uniqueness

We show here that the linearised system (2.6)–(2.9) admits at most one solution. By linearity, it enough to check that if $(\theta ,\nu )$ is a solution to (2.6)–(2.9) in the sense of Theorem 2.5 with ${{\mathbf {h}}}=0$, then $\theta =\nu =0$. To this end, we note that (2.6) yields $\theta _{\mathcal {O}}=0$, so that Itô’s formula gives

$$\begin{aligned}&\frac{1}{2}\left\| \nabla {\mathcal {N}}\theta (t)\right\| _H^2 + \int _{Q_t}|\nabla \theta |^2+\int _{Q_t}\Psi ''(\varphi )|\theta |^2\\&\quad =\int _{Q_t}\theta {{\mathbf {u}}}\cdot \nabla {\mathcal {N}}\theta +\int _0^t\left( {\mathcal {N}}\theta (s), DB(\varphi (s))\theta (s)\right) _H\\&\qquad + \frac{1}{2}\int _0^t\left\| \nabla {\mathcal {N}}DB(\varphi (s))\theta (s)\right\| _{{\mathscr {L}}^2(K,H)}^2\,{\mathrm d}s. \end{aligned}$$

Now, we can argue on the same line of Sect. 5.1 by using assumption **A1** on $\Psi ''$, **C2** on *DB*, together with Burkholder–Davis–Gundy and Young inequalities to get

$$\begin{aligned} \left\| \theta \right\| _{L^p_{\mathscr {P}}(\Omega ; C^0([0,T]; V_1^*)\cap L^2(0,T; V_1))}\le 0\,, \end{aligned}$$

from which $\theta =0$, and also $\nu =0$ by comparison in (2.7). This show that the linearised system (2.6)–(2.9) admits at most one solution.

### Gâteaux-Differentiability

We prove here that $S_1$ is Gâteaux-differentiable. Let ${{\mathbf {u}}}\in {\widetilde{{\mathcal {U}}}}_{ad}$ and ${{\mathbf {h}}}\in {\mathcal {U}}$ be arbitrary and fixed: since ${\widetilde{{\mathcal {U}}}}_{ad}$ is open in ${\mathcal {U}}$, there exists $\delta _0>0$ such that ${{\mathbf {u}}}+\delta {{\mathbf {h}}}\in {\widetilde{{\mathcal {U}}}}_{ad}$ for all $\delta \in [-\delta _0, \delta _0]$. For every such $\delta$, setting $(\varphi _\delta , \mu _\delta ):=S({{\mathbf {u}}}+\delta {{\mathbf {h}}})$ and $(\varphi , \mu ):=S({{\mathbf {u}}})$, the difference of the respective equations (for $\delta \ne 0$) gives

$$\begin{aligned}&{\mathrm d}\left( \frac{\varphi _\delta -\varphi }{\delta }\right) - \Delta \left( \frac{\mu _\delta -\mu }{\delta }\right) \,{\mathrm d}t +{{\mathbf {u}}}\cdot \nabla \left( \frac{\varphi _\delta -\varphi }{\delta }\right) \,{\mathrm d}t +{{\mathbf {h}}}\cdot \nabla \varphi _\delta \,{\mathrm d}t= \frac{B(\varphi _\delta )-B(\varphi )}{\delta }\,{\mathrm d}W\,,\\&\quad \frac{\mu _\delta -\mu }{\delta } = -\Delta \left( \frac{\varphi _\delta -\varphi }{\delta } \right) +\frac{\Psi '(\varphi _\delta )-\Psi '(\varphi )}{\delta }\,,\\&\quad \left( \frac{\varphi _\delta -\varphi }{\delta }\right) (0)=0\,, \end{aligned}$$

whose natural variational formulation reads

5.12 $$\begin{aligned}&\left( \frac{\varphi _\delta -\varphi }{\delta }(t),\zeta \right) _H - \int _{Q_t}\frac{\mu _\delta -\mu }{\delta }\Delta \zeta -\int _{Q_t}(\varphi _\delta {{\mathbf {h}}}+ \frac{\varphi _\delta -\varphi }{\delta }{{\mathbf {u}}})\cdot \nabla \zeta \nonumber \\&\quad = \left( \int _0^t\frac{B(\varphi _\delta (s))-B(\varphi (s))}{\delta }\,{\mathrm d}W(s), \zeta \right) _H \qquad \forall \,\zeta \in V_2\,,\qquad \forall \,t\in [0,T]\,,\quad {\mathbb {P}}\text {-a.s.} \end{aligned}$$

Now, by the continuous dependence estimate (2.4), we deduce that there exists a constant $c>0$ independent of $\delta$ such that

$$\begin{aligned}&\left\| \frac{\varphi _\delta -\varphi }{\delta }\right\| _{L^p_{\mathscr {P}}(\Omega ; C^0([0,T]; V_1^*) \cap L^2(0,T; V_1))} \le c\,,\\&\left\| \frac{\varphi _\delta -\varphi }{\delta }\right\| _{L^{p/3}_{\mathscr {P}}(\Omega ; C^0([0,T]; H) \cap L^2(0,T, V_2))}+ \left\| \frac{\mu _\delta -\mu }{\delta }\right\| _{L^{p/3}_{\mathscr {P}}(\Omega ; L^2(0,T; H))}\le c\,, \end{aligned}$$

so that there exist $(\theta _{{\mathbf {h}}}, \nu _{{\mathbf {h}}})$ with

$$\begin{aligned}&\theta _{{\mathbf {h}}}\in L^{p}_w(\Omega ; L^\infty (0,T; V_1^*))\cap L^{p}_{\mathscr {P}}(\Omega ; L^2(0,T; V_1))\cap L^{p/3}_w(\Omega ; L^\infty (0,T; H))\\&\quad \cap L^{p/3}_{\mathscr {P}}(\Omega ; L^2(0,T; V_2))\,,\\&\nu _{{\mathbf {h}}}\in L^{p/3}_{\mathscr {P}}(\Omega ; L^2(0,T; H))\,, \end{aligned}$$

such that, as $\delta \rightarrow 0$ possibly on a subsequence,

5.13 $$\begin{aligned} \frac{\varphi _\delta -\varphi }{\delta }{\mathop {\rightharpoonup }\limits ^{*}}\theta _{{\mathbf {h}}}\qquad&\text {in } L^{p}_w(\Omega ; L^\infty (0,T; V_1^*))\cap L^{p/3}_w(\Omega ; L^\infty (0,T; H))\,, \end{aligned}$$

5.14 $$\begin{aligned} \frac{\varphi _\delta -\varphi }{\delta }\rightharpoonup \theta _{{\mathbf {h}}}\qquad&\text {in } L^{p}_{\mathscr {P}}(\Omega ; L^2(0,T; V_1))\cap L^{p/3}_{\mathscr {P}}(\Omega ; L^2(0,T; V_2))\,,\end{aligned}$$

5.15 $$\begin{aligned} \frac{\mu _\delta -\mu }{\delta }\rightharpoonup \nu _{{\mathbf {h}}}\qquad&\text {in } L^{p/3}_{\mathscr {P}}(\Omega ; L^2(0,T; H)). \end{aligned}$$

It follows in particular that

5.16 $$\begin{aligned}&\varphi _\delta \rightarrow \varphi \quad \text {in } L^p_{\mathscr {P}}(\Omega ; C^0([0,T]; V_1^*)\cap L^2(0,T; V_1)) \nonumber \\&\quad \qquad \qquad \cap L^{p/3}_{\mathscr {P}}(\Omega ; C^0([0,T]; H)\cap L^2(0,T, V_2)). \end{aligned}$$

Furthermore, since ${{\mathbf {u}}}\in {\mathcal {U}}$, by the inclusion $V_1\hookrightarrow L^6({\mathcal {O}})$, the Hölder inequality, and the convergence (5.14), it holds that

5.17 $$\begin{aligned} \left( \frac{\varphi _\delta -\varphi }{\delta }\right) {{\mathbf {u}}}\rightharpoonup \theta _{{\mathbf {h}}}{{\mathbf {u}}}\qquad \text {in } L^p_{\mathscr {P}}(\Omega ; L^{\frac{2p}{p+2}}(0,T;H^d)). \end{aligned}$$

As far as the nonlinear term is concerned, thanks to the mean-value theorem we have

$$\begin{aligned}&\frac{\Psi '(\varphi _\delta ) - \Psi '(\varphi )}{\delta }- \Psi ''(\varphi )\theta _{{\mathbf {h}}}\\&\quad =\frac{\Psi '(\varphi _\delta ) - \Psi '(\varphi ) - \Psi ''(\varphi )(\varphi _\delta -\varphi )}{\delta }+\Psi ''(\varphi )\left( \frac{\varphi _\delta - \varphi }{\delta } - \theta _{{\mathbf {h}}}\right) \\&\quad =\frac{\varphi _\delta - \varphi }{\delta } \int _0^1\left( \Psi ''(\varphi + s(\varphi _\delta -\varphi )) - \Psi ''(\varphi )\right) \,{\mathrm d}s +\Psi ''(\varphi )\left( \frac{\varphi _\delta - \varphi }{\delta } - \theta _{{\mathbf {h}}}\right) . \end{aligned}$$

Now, by the strong convergence (5.16) and the continuity of $\Psi ''$, we have

$$\begin{aligned} \Psi ''(\varphi + s(\varphi _\delta -\varphi )) - \Psi ''(\varphi ) \rightarrow 0 \quad \forall \,s\in [0,1]\,,\quad \text {a.e.~in } \Omega \times (0,T)\times {\mathcal {O}}\,, \end{aligned}$$

where, recalling that by **C1** $\Psi ''$ has quadratic growth, thanks to the embedding $V_1\hookrightarrow L^6({\mathcal {O}})$ the left-hand side is uniformly bounded in the space $L^{p/2}(\Omega ; L^\infty (0,T; L^3({\mathcal {O}})))$, so that

$$\begin{aligned} \int _0^1\left( \Psi ''(\varphi + s(\varphi _\delta -\varphi )) - \Psi ''(\varphi )\right) \,{\mathrm d}s \rightarrow 0 \qquad \text {in } L^{\ell '}_{\mathscr {P}}(\Omega ; L^{\ell ''}(0,T; L^3({\mathcal {O}}))) \end{aligned}$$

for every $\ell '\in [1,p/2)$ and $\ell ''\in [1,+\infty )$. Taking (5.14) into account, we infer in particular that

$$\begin{aligned} \frac{\varphi _\delta - \varphi }{\delta } \int _0^1\left( \Psi ''(\varphi + s(\varphi _\delta -\varphi )) - \Psi ''(\varphi )\right) \,{\mathrm d}s \rightharpoonup 0 \qquad \text {in } L^{\ell '}_{\mathscr {P}}(\Omega ; L^{\ell ''}(0,T; H))\nonumber \\ \end{aligned}$$

for every $\ell '\in [1,p/3)$ and $\ell ''\in [1,2)$. Similarly, thanks to **C1** and the regularity of $\varphi$, we have $\Psi ''(\varphi )\in L^{p/2}(\Omega ; L^\infty (0,T; L^3({\mathcal {O}})))$, and the same argument as above yields

$$\begin{aligned} \Psi ''(\varphi )\left( \frac{\varphi _\delta - \varphi }{\delta } - \theta _{{\mathbf {h}}}\right) \rightharpoonup 0 \qquad \text {in } L^{\ell '}_{\mathscr {P}}(\Omega ; L^{\ell ''}(0,T; H)) \end{aligned}$$

for every $\ell '\in [1,p/3)$ and $\ell ''\in [1,2)$. It follows that

5.18 $$\begin{aligned} \frac{\Psi '(\varphi _\delta ) - \Psi '(\varphi )}{\delta }\rightharpoonup \Psi ''(\varphi )\theta _{{\mathbf {h}}}\quad \text {in } L^{\ell '}_{\mathscr {P}}(\Omega ; L^{\ell ''}(0,T; H)) \qquad \forall \,\ell '\in [1,p/3)\,,\quad \forall \,\ell ''\in [1,2).\nonumber \\ \end{aligned}$$

Lastly, let us handle the stochastic integral. By the Lipschitz-continuity of *B* in **A3**, we have

$$\begin{aligned}&\frac{B(\varphi _\delta )-B(\varphi )}{\delta } - DB(\varphi )\theta _{{\mathbf {h}}}\\&\quad =\frac{B(\varphi _\delta )-B(\varphi ) - DB(\varphi )(\varphi _\delta -\varphi )}{\delta } +DB(\varphi )\left( \frac{\varphi _\delta -\varphi }{\delta } - \theta _{{\mathbf {h}}}\right) \\&\quad =\int _0^1\left( DB(\varphi +s(\varphi _\delta -\varphi ))-DB(\varphi )\right) \frac{\varphi _\delta -\varphi }{\delta }\,{\mathrm d}s +DB(\varphi )\left( \frac{\varphi _\delta -\varphi }{\delta } - \theta _{{\mathbf {h}}}\right) . \end{aligned}$$

Now, the strong convergence (5.16), the continuity and boundedness of *DB* in **C2** imply together with the dominated convergence theorem that

$$\begin{aligned} \int _0^1\left( DB(\varphi +s(\varphi _\delta -\varphi ))-DB(\varphi )\right) \,{\mathrm d}s \rightarrow 0 \qquad \text {in } L^\ell (\Omega ; L^\ell (0,T; {\mathscr {L}}(V_1,{\mathscr {L}}^2(K,H)))) \end{aligned}$$

for every $\ell \in [1,+\infty )$. Since $\frac{\varphi _\delta -\varphi }{\delta }$ is bounded in $L^{p/3}(\Omega ; L^4(0,T; V_1))$ by interpolation of (5.13)–(5.14), it follows that

$$\begin{aligned} \int _0^1\left( DB(\varphi +s(\varphi _\delta -\varphi ))-DB(\varphi )\right) \frac{\varphi _\delta -\varphi }{\delta }\,{\mathrm d}s \rightharpoonup 0 \qquad \text {in } L^\ell (\Omega ; L^2(0,T; {\mathscr {L}}^2(K,H))) \end{aligned}$$

for every $\ell \in [1,p/3)$. Similarly, by the boundedness of *DB* in **C2** and the convergence (5.14), we have also

$$\begin{aligned} DB(\varphi )\left( \frac{\varphi _\delta -\varphi }{\delta } - \theta _{{\mathbf {h}}}\right) \rightharpoonup 0 \qquad \text {in } L^p_{\mathscr {P}}(\Omega ; L^2(0,T; {\mathscr {L}}^2(K,H))). \end{aligned}$$

Hence, we obtain that

5.19 $$\begin{aligned} \frac{B(\varphi _\delta )-B(\varphi )}{\delta } \rightharpoonup DB(\varphi )\theta _{{\mathbf {h}}}\qquad \text {in } L^\ell (\Omega ; L^2(0,T; {\mathscr {L}}^2(K,H)))\quad \forall \,\ell \in [1,p/3).\nonumber \\ \end{aligned}$$

Finally, letting $\delta \rightarrow 0$ in (5.12) using convergences (5.13)–(5.19), we deduce that actually $(\theta _{{\mathbf {h}}}, \nu _{{\mathbf {h}}})$ is the unique solution of the linearised system (2.6)–(2.9) in the sense of Theorem 2.5.

It remains to show now the strong convergence of $\frac{\varphi _\delta -\varphi }{\delta }$. To this end, note that by the Lipschitz-continuity of *B* in **A3** and (5.14), we have

$$\begin{aligned} \left\| \frac{B(\varphi _\delta )-B(\varphi )}{\delta }\right\| _{L^{p/3}(\Omega ; L^\infty (0,T; {\mathscr {L}}^2(K,H)))}\le c\,, \end{aligned}$$

from which, thanks to the classical result (Flandoli and Gatarek 1995, Lem. 2.1) we get

$$\begin{aligned} \left\| \int _0^\cdot \frac{B(\varphi _\delta (s))- B(\varphi (s))}{\delta }\,{\mathrm d}W(s)\right\| _{L^{p/3}_{\mathscr {P}}(\Omega ; W^{r,p/3}(0,T; H))} \le c_r \qquad \forall \,r\in (0,1/2). \end{aligned}$$

By comparison in the equation (5.12) and the estimates proved above, we infer then that

$$\begin{aligned} \left\| \frac{\varphi _\delta -\varphi }{\delta }\right\| _{L^{p/3}(\Omega ; W^{r,p/3}(0,T; V_2^*))} \le c_r \qquad \forall \,r\in (0,1/2). \end{aligned}$$

Now, recalling that by (Simon 1987, Cor. 5), we have

$$\begin{aligned} L^2(0,T; V_2)\cap W^{r,p/3}(0,T; V_2^*) \hookrightarrow L^2(0,T; V_1) \qquad \text {compactly}\,, \end{aligned}$$

so that the laws of $(\frac{\varphi _\delta -\varphi }{\delta })_\delta$ are tight on $L^2(0,T; V_1)$. By using again Lemma 3.12 together with the uniqueness of the limit problem at $\delta =0$, proceeding as in Sect. 3.4, we also get the strong convergence

$$\begin{aligned} \frac{\varphi _\delta -\varphi }{\delta }\rightarrow \theta _{{\mathbf {h}}}\qquad \text {in } L^2(0,T; V_1)\,, \quad {\mathbb {P}}\text {-a.s.} \end{aligned}$$

which in turn yields, together with (5.14), the strong convergence of Theorem 2.5. This proves that $S_1$ is Gâteaux-differentiable, and its derivative is a solution to the linearised system, in the sense of Theorem 2.5.

### Fréchet-Differentiability

We are only left to show the Fréchet-differentiability of $S_1$. To this end, since ${\widetilde{{\mathcal {U}}}}_{ad}$ is open in ${\mathcal {U}}$, there is a ${\mathcal {U}}$-ball $B^{\mathcal {U}}_r({{\mathbf {u}}})$ of radius $r=r_{{\mathbf {u}}}>0$ centred at ${{\mathbf {u}}}$ such that $B^{\mathcal {U}}_r({{\mathbf {u}}})\subset {\widetilde{{\mathcal {U}}}}_{ad}$. For all ${{\mathbf {h}}}\in B^{\mathcal {U}}_r(\mathbf{0})$, we set $(\varphi _{{\mathbf {h}}},\mu _{{\mathbf {h}}}):=S({{\mathbf {u}}}+{{\mathbf {h}}})$, $y_{{\mathbf {h}}}:=\varphi _{{\mathbf {h}}}-\varphi -\theta _{{\mathbf {h}}}$, and $z_{{\mathbf {h}}}:=\mu _{{\mathbf {h}}}-\mu -\nu _{{\mathbf {h}}}$, so that

$$\begin{aligned}&{\mathrm d}y_{{\mathbf {h}}}- \Delta z_{{\mathbf {h}}}\,{\mathrm d}t +{{\mathbf {u}}}\cdot \nabla y_{{\mathbf {h}}}\,{\mathrm d}t +{{\mathbf {h}}}\cdot \nabla (\varphi _{{\mathbf {h}}}-\varphi )\,{\mathrm d}t = (B(\varphi _{{\mathbf {h}}})-B(\varphi )-DB(\varphi )\theta _{{\mathbf {h}}})\,{\mathrm d}W\,,\\&z_{{\mathbf {h}}}=-\Delta y_{{\mathbf {h}}}+ F'(\varphi _{{\mathbf {h}}}) - F'(\varphi ) - F''(\varphi )\theta _{{\mathbf {h}}}. \end{aligned}$$

Noting that $(y_{{\mathbf {h}}})_{\mathcal {O}}=0$, Itô’s formula yields

$$\begin{aligned}&\frac{1}{2}\left\| \nabla {\mathcal {N}}y_{{\mathbf {h}}}(t)\right\| _H^2 + \int _{Q_t}|\nabla y_{{\mathbf {h}}}|^2 +\int _{Q_t}(F'(\varphi _{{\mathbf {h}}}) - F'(\varphi ) \\&\qquad - F''(\varphi )\theta _{{\mathbf {h}}})y_{{\mathbf {h}}}-\int _{Q_t}(\varphi _{{\mathbf {h}}}-\varphi ){{\mathbf {h}}}\cdot \nabla {\mathcal {N}}y_{{\mathbf {h}}}\\&\quad =\int _{Q_t}y_{{\mathbf {h}}}{{\mathbf {u}}}\cdot \nabla {\mathcal {N}}y_{{\mathbf {h}}}+\int _0^t\left( {\mathcal {N}}y_{{\mathbf {h}}}(s), (B(\varphi _{{\mathbf {h}}}(s))-B(\varphi (s))-DB(\varphi (s))\theta _{{\mathbf {h}}}(s))\,{\mathrm d}W(s)\right) _H\\&\qquad +\frac{1}{2}\int _0^t\left\| \nabla {\mathcal {N}}(B(\varphi _{{\mathbf {h}}}(s))- B(\varphi (s))-DB(\varphi (s))\theta _{{\mathbf {h}}}(s))\right\| _{{\mathscr {L}}^2(K,H)}^2\,{\mathrm d}s\\&\qquad \quad \forall \,t\in [0,T]\,,\quad {\mathbb {P}}\text {-a.s.} \end{aligned}$$

Now, the Young and Hölder inequalities give, together with the embedding $V_1\hookrightarrow L^6({\mathcal {O}})$,

$$\begin{aligned} \int _{Q_t}y_{{\mathbf {h}}}{{\mathbf {u}}}\cdot \nabla {\mathcal {N}}y_{{\mathbf {h}}}\le \varepsilon \int _{Q_t}|\nabla y_{{\mathbf {h}}}|^2 +c_\varepsilon \int _0^t\left( 1+\left\| {{\mathbf {u}}}(s)\right\| _U^2\right) \left\| \nabla {\mathcal {N}}y_{{\mathbf {h}}}(s)\right\| _H^2\,{\mathrm d}s \qquad \forall \,\varepsilon >0 \end{aligned}$$

and similarly

$$\begin{aligned} \int _{Q_t}(\varphi _{{\mathbf {h}}}-\varphi ){{\mathbf {h}}}\cdot \nabla {\mathcal {N}}y_{{\mathbf {h}}}\le \int _{Q_t}|\nabla {\mathcal {N}}y_{{\mathbf {h}}}|^2 + c\left\| \varphi _{{\mathbf {h}}}-\varphi \right\| _{L^4(0,T; V_1)}^2 \left\| {{\mathbf {h}}}\right\| _{L^4(0,T; U)}^2. \end{aligned}$$

Moreover, note that by the mean value theorem and assumption **A1** we have

$$\begin{aligned}&\int _{Q_t}(F'(\varphi _{{\mathbf {h}}}) - F'(\varphi ) - F''(\varphi )\theta _{{\mathbf {h}}})y_{{\mathbf {h}}}\\&\quad =\int _{Q_t}\int _0^1F''(\varphi +\sigma (\varphi _{{\mathbf {h}}}-\varphi )) |y_{{\mathbf {h}}}|^2\,{\mathrm d}\sigma \\&\qquad + \int _{Q_t}\int _0^1\left( F''(\varphi +\sigma (\varphi _{{\mathbf {h}}}-\varphi ))-F''(\varphi )\right) \theta _{{\mathbf {h}}}y_{{\mathbf {h}}}\,{\mathrm d}\sigma \\&\quad \ge -C_\Psi \int _{Q_t}|y_{{\mathbf {h}}}|^2 +\int _{Q_t}\int _0^1\int _0^1 F'''(\varphi +\sigma \tau (\varphi _{{\mathbf {h}}}-\varphi ))\sigma (\varphi _{{\mathbf {h}}}-\varphi ) \theta _{{\mathbf {h}}}y_{{\mathbf {h}}}\,{\mathrm d}\tau \,{\mathrm d}\sigma \,, \end{aligned}$$

where, by the Hölder inequality, the compactness inequality (2.1), the embedding $V_1\hookrightarrow L^6({\mathcal {O}})$, and assumption **C1**,

$$\begin{aligned}&\int _{Q_t}\int _0^1\int _0^1 F'''(\varphi +\sigma \tau (\varphi _{{\mathbf {h}}}-\varphi ))\sigma (\varphi _{{\mathbf {h}}}-\varphi ) \theta _{{\mathbf {h}}}y_{{\mathbf {h}}}\,{\mathrm d}\tau \,{\mathrm d}\sigma \\&\quad \le c\int _0^t\left( 1+\left\| \varphi (s)\right\| _{L^6({\mathcal {O}})} +\left\| \varphi _{{\mathbf {h}}}(s)\right\| _{L^6({\mathcal {O}})}\right) \left\| (\varphi _{{\mathbf {h}}}-\varphi )(s)\right\| _{L^6({\mathcal {O}})} \left\| \theta _{{\mathbf {h}}}(s)\right\| _{L^6({\mathcal {O}})}\left\| y_{{\mathbf {h}}}(s)\right\| _H \,{\mathrm d}s \\&\quad \le \varepsilon \int _{Q_t}|\nabla y_{{\mathbf {h}}}|^2 + c_\varepsilon \int _{Q_t}|\nabla {\mathcal {N}}y_{{\mathbf {h}}}|^2\\&\qquad +c\left( 1+\left\| \varphi \right\| _{L^\infty (0,T;V_1)}^2 +\left\| \varphi _{{\mathbf {h}}}\right\| ^2_{L^\infty (0,T; V_1)}\right) \left\| \varphi -\varphi _{{\mathbf {h}}}\right\| ^2_{L^4(0,T; V_1)} \left\| \theta _{{\mathbf {h}}}\right\| ^2_{L^4(0,T; V_1)}. \end{aligned}$$

Lastly, we have

$$\begin{aligned} B(\varphi _{{\mathbf {h}}})-B(\varphi )-DB(\varphi )\theta _{{\mathbf {h}}}=&\int _0^1\left[ DB(\varphi +\sigma (\varphi _{{\mathbf {h}}}-\varphi ))y_{{\mathbf {h}}}\right. \\&\left. + \left( DB(\varphi +\sigma (\varphi _{{\mathbf {h}}}-\varphi ))-DB(\varphi )\right) \theta _{{\mathbf {h}}}\right] \,{\mathrm d}\sigma \end{aligned}$$

so that by **A3**, **C2–C3**, and the compactness inequality (2.1),

$$\begin{aligned}&\frac{1}{2}\int _0^t\left\| B(\varphi _{{\mathbf {h}}}(s))-B(\varphi (s))-DB(\varphi (s)) \theta _{{\mathbf {h}}}(s)\right\| _{{\mathscr {L}}^2(K,H)}^2\,{\mathrm d}s\\&\quad \le C_B^2\int _{Q_t}|y_{{\mathbf {h}}}|^2+ c\int _0^t\left\| (\varphi _{{\mathbf {h}}}-\varphi )(s)\right\| _{V_1}^2\left\| \theta _{{\mathbf {h}}}(s)\right\| ^2_{V_1}\,{\mathrm d}s\\&\quad \le \varepsilon \int _{Q_t}|\nabla y_{{\mathbf {h}}}|^2 + c_\varepsilon \int _{Q_t}|\nabla {\mathcal {N}}y_{{\mathbf {h}}}|^2 +c\left\| \varphi -\varphi _{{\mathbf {h}}}\right\| ^2_{L^4(0,T; V_1)}\left\| \theta _{{\mathbf {h}}}\right\| ^2_{L^4(0,T; V_1)}. \end{aligned}$$

Consequently, taking all this information into account, we can choose $\varepsilon$ small enough and rearrange the terms to get

$$\begin{aligned}&\frac{1}{2}\left\| \nabla {\mathcal {N}}y_{{\mathbf {h}}}(t)\right\| ^2_H + \int _{Q_t}|\nabla y_{{\mathbf {h}}}|^2\\&\quad \le \int _0^t\left( 1+\left\| {{\mathbf {u}}}(s)\right\| _U^2\right) \left\| \nabla {\mathcal {N}}y_{{\mathbf {h}}}(s)\right\| _H^2\,{\mathrm d}s +c\left\| \varphi _{{\mathbf {h}}}-\varphi \right\| _{L^4(0,T; V_1)}^2\left\| {{\mathbf {h}}}\right\| _{L^4(0,T; U)}^2\\&\qquad + c\left( 1+\left\| \varphi \right\| _{L^\infty (0,T;V_1)}^2 +\left\| \varphi _{{\mathbf {h}}}\right\| ^2_{L^\infty (0,T; V_1)}\right) \left\| \varphi -\varphi _{{\mathbf {h}}}\right\| ^2_{L^4(0,T; V_1)} \left\| \theta _{{\mathbf {h}}}\right\| ^2_{L^4(0,T; V_1)}\\&\qquad +\int _0^t\left( {\mathcal {N}}y_{{\mathbf {h}}}(s), (B(\varphi _{{\mathbf {h}}}(s))-B(\varphi (s))-DB(\varphi (s))\theta _{{\mathbf {h}}}(s))\,{\mathrm d}W(s)\right) _H. \end{aligned}$$

Thanks to the embedding $L^\infty (0,T; H)\cap L^2(0,T; V_2)\hookrightarrow L^4(0,T; V_1)$, by (2.4) and (5.13)–(5.14), we have

$$\begin{aligned} \left\| \varphi _{{\mathbf {h}}}-\varphi \right\| _{L^{p/3}_{\mathscr {P}}(\Omega ; L^4(0,T; V_1))} +\left\| \theta _{{\mathbf {h}}}\right\| _{L^{p/3}_{\mathscr {P}}(\Omega ; L^4(0,T; V_1))}\le c\left\| {{\mathbf {h}}}\right\| _{{\mathcal {U}}}\,, \end{aligned}$$

while (2.2) yields

$$\begin{aligned} \left\| \varphi _{{\mathbf {h}}}\right\| _{L^{p}_{\mathscr {P}}(\Omega ; L^\infty (0,T; V_1))} +\left\| \varphi \right\| _{L^{p}_{\mathscr {P}}(\Omega ; L^\infty (0,T; V_1))}\le c\,, \end{aligned}$$

where the constant *c* is independent of ${{\mathbf {h}}}$. Taking power $\frac{p}{14}$ at both sides, supremum in time and expectations, on the right-hand side we use the Hölder inequality with exponents $\frac{1}{7}+\frac{3}{7}+\frac{3}{7}=1$ to get

$$\begin{aligned}&\left\| \left( 1+\left\| \varphi \right\| _{L^\infty (0,T;V_1)}^{p/7} +\left\| \varphi _{{\mathbf {h}}}\right\| ^{p/7}_{L^\infty (0,T; V_1)}\right) \left\| \varphi -\varphi _{{\mathbf {h}}}\right\| ^{p/7}_{L^4(0,T; V_1)} \left\| \theta _{{\mathbf {h}}}\right\| ^{p/7}_{L^4(0,T; V_1)}\right\| _{L^{1}(\Omega )}\\&\quad \le c\left\| {{\mathbf {h}}}\right\| _{{\mathcal {U}}}^{2p/7} \end{aligned}$$

and similarly

$$\begin{aligned} \left\| \left\| \varphi _{{\mathbf {h}}}-\varphi \right\| _{L^4(0,T; V_1)}^{p/7} \left\| {{\mathbf {h}}}\right\| _{L^4(0,T; U)}^{p/7}\right\| _{L^1(\Omega )}\le c\left\| {{\mathbf {h}}}\right\| _{{\mathcal {U}}}^{2p/7}. \end{aligned}$$

Consequently, arguing again as in Sect. 3.1, using an iterative argument and the Burkholder–Davis–Gundy and Young inequalities (see also Marinelli and Scarpa 2020, Lem. 4.1) gives then

$$\begin{aligned} \left\| y_{{\mathbf {h}}}\right\| _{L^{p/7}(\Omega ; C^0([0,T]; V_1^*)\cap L^2(0,T; V_1))} \le c\left\| {{\mathbf {h}}}\right\| _{{\mathcal {U}}}^2=o\left( \left\| {{\mathbf {h}}}\right\| _{{\mathcal {U}}}\right) \qquad \text {as } \left\| {{\mathbf {h}}}\right\| _{\mathcal {U}}\rightarrow 0. \end{aligned}$$

This proves the Fréchet-differentiability of $S_1$ and concludes the proof of Theorem 2.5.

## Adjoint System

In this section, we study the adjoint problem (2.10)–(2.13), proving that it is well posed in the sense of Theorem 2.6.

As we have anticipated in Introduction, the presence of the extra-random component in the convection term calls for non-trivial mathematical tools when deriving estimates on the solutions. Let us recall here a general backward version of the stochastic Gronwall lemma that will be used in this section: for details we refer to (Hun et al. 2020, Thm. 1) and (Wang and Fan 2018).

### Lemma 6.1

Let $\xi \in L^2(\Omega ,{\mathscr {F}}_T)$ be non-negative, $\alpha \in L^\infty _{\mathscr {P}}(\Omega ; L^1(0,T))$ with $\alpha \ge \alpha _0>0$ almost everywhere in $\Omega \times (0,T)$, and $X\in L^2_{\mathscr {P}}(\Omega ; C^0([0,T]))$ be a non-negative process such that

$$\begin{aligned} X(t) \le {{\mathbb {E}}}\left[ \xi + \int _t^T\alpha (s)X(s)\,{\mathrm d}s\,\Bigg |{\mathscr {F}}_t\right] \qquad \forall \,t\in [0,T]\,,\quad {\mathbb {P}}\text {-a.s.} \end{aligned}$$

Then, for every $t\in [0,T]$ it holds that

$$\begin{aligned} X(t) \le {{\mathbb {E}}}\left[ \xi \exp \left\| \alpha \right\| _{L^1(t,T)}\,\Big |{\mathscr {F}}_t\right] \qquad {\mathbb {P}}\text {-a.s.} \end{aligned}$$

### Approximation

For every $\lambda >0$, using the approximations on $\Psi$ and ${{\mathbf {u}}}$ as in Sect. 3.2, we consider the approximated problem

6.1 $$\begin{aligned} -{\mathrm d}P_\lambda -\Delta {\tilde{P}}_\lambda \,{\mathrm d}t + \Psi _\lambda ''(\varphi ){\tilde{P}}_\lambda \,{\mathrm d}t - {{\mathbf {u}}}_\lambda \cdot \nabla P_\lambda \,{\mathrm d}t \qquad \qquad&\nonumber \\ =\alpha _1(\varphi -\varphi _Q)\,{\mathrm d}t+ DB(\varphi )^*Z_\lambda \,{\mathrm d}t - Z_\lambda \,{\mathrm d}W \qquad&\text {in } (0,T)\times {\mathcal {O}}\,, \end{aligned}$$

6.2 $$\begin{aligned} {\tilde{P}}_\lambda =-\Delta P_\lambda \qquad&\text {in } (0,T)\times {\mathcal {O}}\,, \end{aligned}$$

6.3 $$\begin{aligned} \mathbf{n}\cdot \nabla P_\lambda = \mathbf{n}\cdot \nabla {\tilde{P}}_\lambda = 0 \qquad&\text {in } (0,T)\times \partial {\mathcal {O}}\,, \end{aligned}$$

6.4 $$\begin{aligned} P_\lambda (T)=\alpha _2(\varphi (T)-\varphi _T) \qquad&\text {in } {\mathcal {O}}. \end{aligned}$$

This can be written in abstract form as:

$$\begin{aligned}&-{\mathrm d}P_\lambda + {\mathcal {F}}_\lambda (P_\lambda )\,{\mathrm d}t= \alpha _1(\varphi -\varphi _Q)\,{\mathrm d}t + DB(\varphi )^*Z_\lambda \,{\mathrm d}t - Z_\lambda \,{\mathrm d}W\,, \\&\quad P_\lambda (T)=\alpha _2(\varphi (T)-\varphi _T)\,, \end{aligned}$$

where ${\mathcal {F}}_\lambda :\Omega \times [0,T]\times V_2\rightarrow V_2^*$ is given by

$$\begin{aligned} \left\langle {\mathcal {F}}_\lambda (\omega ,t,y),\zeta \right\rangle := \int _{\mathcal {O}}\left( \Delta y\Delta \zeta -\Psi _\lambda ''(\varphi (\omega ,t))\Delta y\zeta + y{{\mathbf {u}}}_\lambda (\omega ,t)\cdot \nabla \zeta \right) \,,\qquad y,\zeta \in V_2. \end{aligned}$$

By construction it holds that $\Psi ''_\lambda (\varphi )\in L^\infty (\Omega \times Q)$ and ${{\mathbf {u}}}_\lambda \in L^\infty _{\mathscr {P}}(\Omega \times (0,T); U)$, so that using similar arguments to the ones in Sect. 3.2, we have that the operator ${\mathcal {F}}_\lambda$ is progressively measurable, hemicontinuous, weakly monotone, weakly coercive, and linearly bounded. Moreover, the Lipschitz-continuity of *B* in **A3** implies that $DB(\varphi )^*$ is uniformly bounded as well. The classical variational theory for backward SPDEs (Du and Meng 2010, Sec. 3) ensures then that such approximated problem admits a unique variational solution $(P_\lambda , Q_\lambda )$, with

$$\begin{aligned} P_\lambda \in L^2_{{\mathscr {P}}}(\Omega ; C^0([0,T]; H)\cap L^2(0,T; V_2))\,, \qquad Z_\lambda \in L^2_{\mathscr {P}}(\Omega ; L^2(0,T; {\mathscr {L}}^2(U,H))). \end{aligned}$$

Actually, let us note that thanks to the assumption on the target $\varphi _T$ and the regularity of $\varphi$, the final value satisfies $\alpha _2(\varphi (T)-\varphi _T)\in L^2(\Omega , {\mathscr {F}}_T; V_1)$. Consequently, by a standard finite dimensional approximation of the approximated problem with $\lambda >0$ fixed, it follows that the approximated solution actually inherits more regularity, namely

$$\begin{aligned} P_\lambda \in L^2_{{\mathscr {P}}}(\Omega ; C^0([0,T]; V_1)\cap L^2(0,T; V_3))\,, \qquad Z_\lambda \in L^2_{\mathscr {P}}(\Omega ; L^2(0,T; {\mathscr {L}}^2(U,V_1))). \end{aligned}$$

We can then set

$$\begin{aligned} {\tilde{P}}_\lambda :={\mathcal {L}}P_\lambda \in L^2_{{\mathscr {P}}}(\Omega ; C^0([0,T]; V_1^*)\cap L^2(0,T; V_1))\,, \end{aligned}$$

so that $(P_\lambda , {\tilde{P}}_\lambda , Z_\lambda )$ satisfy, for every $t\in [0,T]$, ${\mathbb {P}}$-almost surely, for every $\zeta \in V_1$,

$$\begin{aligned}&\left( P_\lambda (t), \zeta \right) _H +\int _{Q_t^T}\nabla {\tilde{P}}_\lambda \cdot \nabla \zeta +\int _{Q_t^T}\Psi _\lambda ''(\varphi ){\tilde{P}}_\lambda \zeta +\int _{Q_t^T}P_\lambda {{\mathbf {u}}}_\lambda \cdot \nabla \zeta \\&\quad =\left( \alpha _2(\varphi (T)-\varphi _T), \zeta \right) _H +\int _{Q_t^T}\alpha _1(\varphi -\varphi _Q)\zeta \\&\qquad +\int _{Q_t^T}DB(\varphi )^*Z_\lambda \zeta -\left( \int _t^TZ_\lambda (s)\,{\mathrm d}W(s), \zeta \right) _H. \end{aligned}$$

### An Estimate by Duality Method

The first estimate that we prove is based on a duality method between the approximated adjoint system (6.1)–(6.4) and a suitably introduced approximated linearised system. This step is fundamental as it allows to obtain some preliminary estimates on the adjoint variables without working explicitly on the adjoint system, which may be not trivial. Such duality method is extremely powerful, and it will be crucial in showing well-posedness of the adjoint system.

The idea is the following: we consider the $\lambda$-approximated version of the linearised system (2.6)–(2.9), in a more general version where the forcing term is given by an arbitrary term

$$\begin{aligned} g\in L^{\frac{2p}{p+4}}_{\mathscr {P}}(\Omega ; L^2(0,T; H)). \end{aligned}$$

Namely, for ${{\mathbf {h}}}\in {\mathcal {U}}$ we consider

6.5 $$\begin{aligned} {\mathrm d}\theta _{{{\mathbf {h}}},\lambda }^g - \Delta \nu _{{{\mathbf {h}}},\lambda }^g\,{\mathrm d}t +{{\mathbf {h}}}\cdot \nabla \varphi \,{\mathrm d}t + {{\mathbf {u}}}_\lambda \cdot \nabla \theta ^g_{{{\mathbf {h}}},\lambda }\,{\mathrm d}t = DB(\varphi )\theta ^g_{{{\mathbf {h}}},\lambda }\,{\mathrm d}W \qquad&\text {in } (0,T)\times {\mathcal {O}}\,, \end{aligned}$$

6.6 $$\begin{aligned} \nu _{{{\mathbf {h}}},\lambda }^g=-\Delta \theta _{{{\mathbf {h}}},\lambda }^g + \Psi _\lambda ''(\varphi )\theta _{{{\mathbf {h}}},\lambda }^g -g \qquad&\text {in } (0,T)\times {\mathcal {O}}\,, \end{aligned}$$

6.7 $$\begin{aligned} \mathbf{n}\cdot \nabla \theta _{{{\mathbf {h}}},\lambda }^g = \mathbf{n}\cdot \nabla \nu _{{{\mathbf {h}}},\lambda }^g = 0 \qquad&\text {in } (0,T)\times \partial {\mathcal {O}}\,, \end{aligned}$$

6.8 $$\begin{aligned} \theta _{{{\mathbf {h}}},\lambda }^g(0)=0 \qquad&\text {in } {\mathcal {O}}. \end{aligned}$$

Since $\Psi ''_\lambda (\varphi )\in L^\infty (\Omega \times Q)$, the classical variational approach (see again Sects. 3.2 and 5.1) ensures that the system (6.5)–(6.8) admits a unique solution

$$\begin{aligned}&\theta _{{{\mathbf {h}}},\lambda }^g \in L^{\frac{2p}{p+4}}_{{\mathscr {P}}}\left( \Omega ; C^0([0,T]; H)\cap L^2(0,T; V_2)\right) \,,\qquad \nu _{{{\mathbf {h}}},\lambda }^g\in L^{\frac{2p}{p+4}}_{{\mathscr {P}}}(\Omega ; L^2(0,T; H)). \end{aligned}$$

Moreover, we can show that the system (6.5)–(6.8) is in duality with the approximated adjoint system (6.1)–(6.4). To this end, by Itô’s formula we have that

$$\begin{aligned}&{\mathrm d}(\theta _{{{\mathbf {h}}},\lambda }^g,P_\lambda )_H\\&\quad = -{\tilde{P}}_\lambda \nu _{{{\mathbf {h}}},\lambda }^g\,{\mathrm d}t +\varphi {{\mathbf {h}}}\cdot \nabla P_\lambda \,{\mathrm d}t +\theta _{{{\mathbf {h}}},\lambda }^g{{\mathbf {u}}}_\lambda \cdot \nabla P_\lambda \,{\mathrm d}t +(P_\lambda , DB(\varphi )\theta _{{{\mathbf {h}}},\lambda }^g\,{\mathrm d}W)_H\\&\qquad +{\tilde{P}}_\lambda (-\Delta \theta _{{{\mathbf {h}}},\lambda }^g+ \Psi ''_\lambda (\varphi )\theta _{{{\mathbf {h}}},\lambda }^g)\,{\mathrm d}t +P_\lambda {{\mathbf {u}}}_\lambda \cdot \nabla \theta _{{{\mathbf {h}}},\lambda }^g\,{\mathrm d}t -\alpha _1(\varphi -\varphi _Q)\theta _{{{\mathbf {h}}},\lambda }^g\,{\mathrm d}t\\&\qquad -(DB(\varphi )^*Z_\lambda , \theta _{{{\mathbf {h}}},\lambda }^g)_H\,{\mathrm d}t + (\theta _{{{\mathbf {h}}},\lambda }^g, Z_\lambda \,{\mathrm d}W)_H +(DB(\varphi )\theta _{{{\mathbf {h}}},\lambda }^g, Z_\lambda )_{{\mathscr {L}}^2(K,H)}\,{\mathrm d}t\,, \end{aligned}$$

which readily implies by comparison in the two systems that

6.9 $$\begin{aligned} \alpha _1{{\mathbb {E}}}\int _Q\theta _{{{\mathbf {h}}},\lambda }^g(\varphi -\varphi _Q)+ \alpha _2{{\mathbb {E}}}\int _{\mathcal {O}}\theta _{{{\mathbf {h}}},\lambda }^g(T)(\varphi (T)-\varphi _T)= {{\mathbb {E}}}\int _Q\varphi {{\mathbf {h}}}\cdot \nabla P_\lambda + {{\mathbb {E}}}\int _Q{\tilde{P}}_\lambda g.\nonumber \\ \end{aligned}$$

Let us set now for brevity of notation $\theta _\lambda ^g:=\theta ^g_{{{\mathbf {h}}},\lambda }$ and $\nu _\theta ^g:=\nu _{{{\mathbf {h}}},\lambda }^g$ with the choice ${{\mathbf {h}}}=0$. Noting that $(\theta _\lambda ^g)_{\mathcal {O}}=0$, Itô’s formula for $\frac{1}{2}\left\| \nabla {\mathcal {N}}\theta _\lambda ^g\right\| _H^2$ yields

$$\begin{aligned}&\frac{1}{2}\left\| \nabla {\mathcal {N}}\theta _\lambda ^g(t)\right\| _H^2 + \int _{Q_t}|\nabla \theta _\lambda ^g|^2 = \int _{Q_t}\theta _\lambda ^g{{\mathbf {u}}}_\lambda \cdot \nabla {\mathcal {N}}\theta _\lambda ^g -\int _{Q_t}\Psi ''_\lambda (\varphi )|\theta _\lambda ^g|^2 +\int _{Q_t}g\theta _\lambda ^g\\&\quad +\frac{1}{2}\int _0^t\left\| \nabla {\mathcal {N}}DB(\varphi (s))\theta _\lambda ^g(s)\right\| _{{\mathscr {L}}^2(K,H)}^2\,{\mathrm d}s\\&\quad +\int _0^t\left( {\mathcal {N}}\theta _\lambda ^g(s),DB(\varphi (s))\theta _\lambda ^g(s)\,{\mathrm d}W(s)\right) _H. \end{aligned}$$

Using the fact that $\Psi ''_\lambda \ge -C_\Psi$ and the boundedness of $DB(\varphi )$ in ${\mathscr {L}}(V_1, {\mathscr {L}}^2(K,H))$, thanks to the Hölder–Young inequalities and the compactness inequality (2.1) we get, for all $\varepsilon >0$,

$$\begin{aligned}&\left\| \nabla {\mathcal {N}}\theta _\lambda ^g(t)\right\| _H^2 + \int _{Q_t}|\nabla \theta _\lambda ^g|^2 \\&\quad \le \int _Q|g|^2 + \varepsilon \int _{Q_t}|\nabla \theta _\lambda ^g|^2 + c_\varepsilon \int _0^t\left( 1+\left\| {{\mathbf {u}}}(s)\right\| _U^2\right) \left\| \nabla {\mathcal {N}}\theta _\lambda ^g(s)\right\| _H^2\,{\mathrm d}s\\&\qquad +\int _0^t\left( {\mathcal {N}}\theta _\lambda ^g(s),DB(\varphi (s))\theta _\lambda ^g(s)\,{\mathrm d}W(s)\right) _H. \end{aligned}$$

We take now power $\frac{p}{p+4}$ at both sides, supremum in time, and expectations. Thanks to the Burkholder–Davis–Gundy inequality (see Marinelli and Scarpa 2020, Lem. 4.1), assumption **C2**, and (2.1), we get

$$\begin{aligned}&{{\mathbb {E}}}\sup _{r\in [0,t]}\left| \int _0^r\left( {\mathcal {N}}\theta _\lambda ^g(s), DB(\varphi (s))\theta _\lambda ^g(s)\,{\mathrm d}W(s)\right) _H\right| ^{\frac{p}{p+4}}\\&\quad \le \frac{1}{2}{{\mathbb {E}}}\left\| \nabla {\mathcal {N}}\theta _\lambda ^g\right\| _{L^\infty (0,t; H)}^{\frac{2p}{p+4}} +c{{\mathbb {E}}}\left\| \theta _\lambda ^g\right\| _{L^2(0,t; H)}^{\frac{2p}{p+4}}\\&\quad \le \frac{1}{2}{{\mathbb {E}}}\left\| \nabla {\mathcal {N}}\theta _\lambda ^g\right\| _{L^\infty (0,t; H)}^{\frac{2p}{p+4}} +\frac{1}{2}{{\mathbb {E}}}\left\| \nabla \theta _\lambda ^g\right\| _{L^2(0,t; H)}^{\frac{2p}{p+4}} +ct^{\frac{p}{p+4}}{{\mathbb {E}}}\left\| \nabla {\mathcal {N}}\theta _\lambda ^g\right\| _{L^\infty (0,t; H)}^{\frac{2p}{p+4}}. \end{aligned}$$

Moreover, since ${{\mathbf {u}}}\in {\widetilde{{\mathcal {U}}}}_{ad}$, by the Hölder inequality we have

$$\begin{aligned}&{{\mathbb {E}}}\sup _{r\in [0,t]}\left| \int _0^r\left( 1+\left\| {{\mathbf {u}}}(s)\right\| _U^2\right) \left\| \nabla {\mathcal {N}}\theta _\lambda ^g(s)\right\| _H^2\,{\mathrm d}s\right| ^{\frac{p}{p+4}}\\&\quad \le c{{\mathbb {E}}}\left| t^{1-\frac{2}{p}}\left( 1+\left\| {{\mathbf {u}}}\right\| _{L^p(0,T; U)}^2\right) \left\| \nabla {\mathcal {N}}\theta _{\lambda }^g\right\| _{L^\infty (0,t; H)}^2\right| ^{\frac{p}{p+4}} \le ct^{\frac{p-2}{p+4}}{{\mathbb {E}}}\left\| \nabla {\mathcal {N}}\theta _{\lambda }^g\right\| _{L^\infty (0,t; H)}^{\frac{2p}{p+4}}. \end{aligned}$$

Since $\frac{p}{p+4}>0$ and $\frac{p-2}{p+4}>0$, we can close the estimate rearranging all the terms on $[0,T_0]$ for $T_0$ sufficiently small (independent of both $\lambda$ and *g*). Using once more a classical iterative procedure on every subinterval until *T*, we infer that there exists a constant $c>0$, independent of both $\lambda$ and *g*, such that

6.10 $$\begin{aligned} \left\| \theta _\lambda ^g\right\| _{L^{\frac{2p}{p+4}}_{\mathscr {P}}(\Omega ; C^0([0,T]; V_1^*)\cap L^2(0,T; V_1))} \le c\left\| g\right\| _{L^{\frac{2p}{p+4}}_{\mathscr {P}}(\Omega ; L^2(0,T; H))}. \end{aligned}$$

Now, by assumption **C4** and the regularity of $\varphi$ (since $\frac{2p}{p-4}\le p$ for $p\ge 6$), it holds

$$\begin{aligned} \alpha _1(\varphi -\varphi _Q)\in L^{\frac{2p}{p-4}}_{\mathscr {P}}(\Omega ; L^2(0,T; H))\,,\qquad \alpha _2(\varphi (T)-\varphi _T)\in L^{\frac{2p}{p-4}}(\Omega ,{\mathscr {F}}_T; V_1)\,, \end{aligned}$$

so that the duality relation (6.9) (with ${{\mathbf {h}}}=0$) and the estimate (6.10) yield

$$\begin{aligned} {{\mathbb {E}}}\int _Q{\tilde{P}}_\lambda g&\le \left\| \theta _\lambda ^g\right\| _{L^{\frac{2p}{p+4}}_{\mathscr {P}}(\Omega ; L^2(0,T; H))} \left\| \alpha _1(\varphi -\varphi _Q)\right\| _{L^{\frac{2p}{p-4}}_{\mathscr {P}}(\Omega ; L^2(0,T; H))} \\&\quad + \left\| \theta _\lambda ^g\right\| _{L^{\frac{2p}{p+4}}_{\mathscr {P}}(\Omega ; C^0([0,T]; V_1^*))} \left\| \alpha _2(\varphi (T)-\varphi _T)\right\| _{L^{\frac{2p}{p-4}}(\Omega ,{\mathscr {F}}_T;V_1)}\\&\le c\left\| g\right\| _{L^{\frac{2p}{p+4}}_{\mathscr {P}}(\Omega ; L^2(0,T; H))}. \end{aligned}$$

By the arbitrariness of *g* we obtain

6.11 $$\begin{aligned} \Vert {\tilde{P}}_\lambda \Vert _{L^{\frac{2p}{p-4}}_{\mathscr {P}}(\Omega ; L^2(0,T; H))} \le c. \end{aligned}$$

### Further Estimates

We show here that the initial estimate (6.11) allows to obtain uniform estimates on the adjoint variables. To this end, Itô’s formula for $\frac{1}{2}\left\| P_\lambda \right\| _H^2+ \frac{1}{2}\left\| \nabla P_\lambda \right\| _H^2$ yields, recalling that ${\tilde{P}}_\lambda ={\mathcal {L}} P_\lambda$,

6.12 $$\begin{aligned}&\frac{1}{2}\left\| P_\lambda (t)\right\| _{V_1}^2 +\int _t^T\Vert {\tilde{P}}_\lambda (s)\Vert _{V_1}^2\,{\mathrm d}s +\frac{1}{2}\int _t^T\left\| Z_\lambda (s)\right\| ^2_{{\mathscr {L}}^2(K,V_1)}\,{\mathrm d}s\nonumber \\&\quad =\frac{\alpha _2^2}{2}\left\| \varphi (T)-\varphi _T\right\| _{V_1}^2 -\int _{Q_t^T}\Psi ''_\lambda (\varphi )|{\tilde{P}}_\lambda |^2 -\int _{Q_t^T}\Psi ''_\lambda (\varphi ){\tilde{P}}_\lambda P_\lambda +\int _{Q_t^T}(P_\lambda + {\tilde{P}}_\lambda ){{\mathbf {u}}}_\lambda \cdot \nabla P_\lambda \nonumber \\&\qquad +\alpha _1\int _{Q_t^T}(\varphi -\varphi _Q)(P_\lambda + {\tilde{P}}_\lambda ) +\int _{Q_t^T}(DB(\varphi )^*Z_\lambda )(P_\lambda + {\tilde{P}}_\lambda )\nonumber \\&\qquad -\int _t^T\left( P_\lambda (s) + {\tilde{P}}_\lambda (s), Z_\lambda (s)\,{\mathrm d}W(s)\right) _H \qquad \forall \,t\in [0,T]\,,\quad {\mathbb {P}}\text {-a.s.} \end{aligned}$$

On the right-hand side, we have already noticed that $\alpha _2(\varphi (T)-\varphi _T)\in L^2(\Omega ,{\mathscr {F}}_T; V_1)$. Moreover, by **A1**, the compactness inequality (2.1) and the fact that ${\tilde{P}}_\lambda ={\mathcal {L}} P_\lambda$, for the second and third terms we have

$$\begin{aligned} -\int _{Q_t^T}\Psi ''_\lambda (\varphi )|{\tilde{P}}_\lambda |^2 \le C_\Psi \int _{Q_t^T}|{\tilde{P}}_\lambda |^2\le \varepsilon \int _{Q_t^T}|\nabla {\tilde{P}}_\lambda |^2 + c_\varepsilon \int _{Q_t}|\nabla P_\lambda |^2 \end{aligned}$$

and, thanks to the Hölder–Young inequalities, the embedding $V_1\hookrightarrow L^6({\mathcal {O}})$, and **C1**,

$$\begin{aligned} -\int _{Q_t^T}\Psi ''_\lambda (\varphi ){\tilde{P}}_\lambda P_\lambda&\le \int _t^T\left\| P_\lambda (s)\right\| _{V_1}^2\,{\mathrm d}s + c\int _t^T\left\| \Psi ''_\lambda (\varphi (s))\right\| _{L^3({\mathcal {O}})}^2\Vert {\tilde{P}}_\lambda (s) \Vert _H^2\,{\mathrm d}s\\&\le \int _t^T\left\| P_\lambda (s)\right\| _{V_1}^2\,{\mathrm d}s + c\left( 1+\left\| \varphi \right\| _{L^\infty (0,T; V_1)}^4\right) \Vert {\tilde{P}}_\lambda \Vert ^2_{L^2(0,T; H)}. \end{aligned}$$

Also, note that since ${\tilde{P}}_\lambda ={\mathcal {L}} P_\lambda$, in particular it holds that $({\tilde{P}}_\lambda )_{\mathcal {O}}=0$. Hence, using the Young and Hölder inequalities, the embedding $V_1\hookrightarrow L^6({\mathcal {O}})$ yields, for all $\varepsilon >0$,

$$\begin{aligned} \int _{Q_t^T}(P_\lambda +{\tilde{P}}_\lambda ){{\mathbf {u}}}_\lambda \cdot \nabla P_\lambda \le \varepsilon \int _t^T\Vert {\tilde{P}}_\lambda (s)\Vert ^2_{V_1}\,{\mathrm d}s +c_\varepsilon \int _t^T\left( 1+\left\| {{\mathbf {u}}}(s)\right\| _U^2\right) \left\| P_\lambda (s)\right\| ^2_{V_1}\,{\mathrm d}s\,, \end{aligned}$$

and similarly

$$\begin{aligned} \alpha _1\int _{Q_t^T}(\varphi -\varphi _Q)(P_\lambda +{\tilde{P}}_\lambda ) \le \alpha _1^2\int _Q|\varphi -\varphi _Q|^2 + \frac{1}{2}\int _{Q_t^T}|P_\lambda |^2+ \frac{1}{2}\int _{Q_t^T}|{\tilde{P}}_\lambda |^2. \end{aligned}$$

Lastly, thanks to **A3** and **C2**, and again the compactness inequality (2.1), we have that

$$\begin{aligned} \int _{Q_t^T}(DB(\varphi )^*Z_\lambda )(P_\lambda +{\tilde{P}}_\lambda )&= \int _t^T\left( Z_\lambda (s), DB(\varphi (s)) (P_\lambda +{\tilde{P}}_\lambda )(s)\right) _{{\mathscr {L}}^2(K,H)}\,{\mathrm d}s\\&\le \frac{1}{4}\int _t^T\left\| Z_\lambda (s)\right\| ^2_{{\mathscr {L}}^2(K,H)}\,{\mathrm d}s +2C_B^2\int _{Q_t^T}|P_\lambda |^2\\&\quad +2C_B^2\int _{Q_t^T}|{\tilde{P}}_\lambda |^2\\&\le \frac{1}{4}\int _t^T\left\| Z_\lambda (s)\right\| ^2_{{\mathscr {L}}^2(K,H)}\,{\mathrm d}s +\varepsilon \int _{Q_t^T}|\nabla {\tilde{P}}_\lambda |^2\\&\quad +c_\varepsilon \int _t^T\left\| P_\lambda (s)\right\| _{V_1}^2\,{\mathrm d}s. \end{aligned}$$

Choosing $\varepsilon$ small enough, rearranging the terms in (6.12), and conditioning (6.12) with respect to ${\mathscr {F}}_t$ we are left with

$$\begin{aligned}&\left\| P_\lambda (t)\right\| _{V_1}^2 +{{\mathbb {E}}}\left[ \int _t^T\Vert {\tilde{P}}_\lambda (s)\Vert _{V_1}^2\,{\mathrm d}s +\int _t^T\left\| Z_\lambda (s)\right\| ^2_{{\mathscr {L}}^2(K,V_1)}\,{\mathrm d}s\,\Bigg | {\mathscr {F}}_t\right] \\&\quad \le c + c {{\mathbb {E}}}\left[ \left( 1+\left\| \varphi \right\| _{L^\infty (0,T; V_1)}^4\right) \Vert {\tilde{P}}_\lambda \Vert ^2_{L^2(0,T; H)}\right. \\&\qquad \left. +\int _t^T\left( 1+\left\| {{\mathbf {u}}}(s)\right\| _U^2\right) \left\| P_\lambda (s)\right\| ^2_{V_1}\,{\mathrm d}s\, \Bigg | {\mathscr {F}}_t\right] \,, \end{aligned}$$

so that the backward version of the stochastic Gronwall Lemma 6.1 yields

$$\begin{aligned}&\left\| P_\lambda (t)\right\| _{V_1}^2 +{{\mathbb {E}}}\left[ \int _t^T\Vert {\tilde{P}}_\lambda (s)\Vert _{V_1}^2\,{\mathrm d}s +\int _t^T\left\| Z_\lambda (s)\right\| ^2_{{\mathscr {L}}^2(K,V_1)}\,{\mathrm d}s\,\Bigg | {\mathscr {F}}_t\right] \\&\quad \le {{\mathbb {E}}}\left[ \left( c+c\left( 1 + \left\| \varphi \right\| _{L^\infty (0,T; V_1)}^4\right) \Vert {\tilde{P}}_\lambda \Vert ^2_{L^2(0,T; H)}\right) \exp \left( t+\left\| {{\mathbf {u}}}\right\| _{L^2(0,T; U)}^2\right) \,\Bigg | {\mathscr {F}}_t\right] . \end{aligned}$$

Consequently, taking expectations we infer that

$$\begin{aligned}&{{\mathbb {E}}}\left\| P_\lambda (t)\right\| _{V_1}^2 + {{\mathbb {E}}}\Vert {\tilde{P}}_\lambda \Vert ^2_{L^2(t,T; V_1)} +{{\mathbb {E}}}\left\| Z_\lambda \right\| ^2_{L^2(t,T; {\mathscr {L}}^2(K,V_1))}\\&\quad \le c\left( 1+\exp \left\| {{\mathbf {u}}}\right\| _{\mathcal {U}}^2\right) {{\mathbb {E}}}\left[ 1+\left( 1+\left\| \varphi \right\| _{L^\infty (0,T; V_1)}^4\right) \Vert {\tilde{P}}_\lambda \Vert ^2_{L^2(0,T; H)}\right] \,, \end{aligned}$$

where, by the Hölder inequality and the duality-estimate (6.11), we have

$$\begin{aligned}&{{\mathbb {E}}}\left[ \left( 1+\left\| \varphi \right\| _{L^\infty (0,T; V_1)}^4\right) \Vert {\tilde{P}}_\lambda \Vert ^2_{L^2(0,T; H)}\right] \\&\quad \le \left\| 1+\left\| \varphi \right\| _{L^\infty (0,T; V_1)}^4\right\| _{L^{\frac{p}{4}}(\Omega )} \left\| \Vert {\tilde{P}}_\lambda \Vert ^2_{L^2(0,T; H)}\right\| _{L^{\frac{p}{p-4}}(\Omega )}\\&\quad \le c\left( 1+\left\| \varphi \right\| ^4_{L^p(\Omega ; L^\infty (0,T; V_1))}\right) \Vert {\tilde{P}}_\lambda \Vert ^2_{L^\frac{2p}{p-4}(\Omega ; L^2(0,T; H))}\le c\,, \end{aligned}$$

which yields in turn

6.13 $$\begin{aligned} \left\| P_\lambda \right\| _{C^0([0,T]; L^2(\Omega ; V_1))} + \Vert {\tilde{P}}_\lambda \Vert _{L^2_{\mathscr {P}}(\Omega ; L^2(0,T; V_1))} +\left\| Z_\lambda \right\| _{L^2_{\mathscr {P}}(\Omega ; L^2(0,T; {\mathscr {L}}^2(K,V_1)))} \le c.\nonumber \\ \end{aligned}$$

With this additional information, we can perform a classical refinement on the estimates going back to the inequality (6.12), repeating the same steps but this time taking first supremum in time and then expectations: the estimate (6.13) allows to apply the Burkholder–Davis–Gundy inequality on the stochastic integral, so that we obtain, thanks also to elliptic regularity,

6.14 $$\begin{aligned} \left\| P_\lambda \right\| _{L^2_{\mathscr {P}}(\Omega ; C^0([0,T]; V_1)\cap L^2(0,T; V_3))}+ \Vert {\tilde{P}}_\lambda \Vert _{L^2_{\mathscr {P}}(\Omega ; C^0([0,T]; V_1^*)\cap L^2(0,T; V_1))}\le c.\nonumber \\ \end{aligned}$$

### Passage to the Limit

From (6.13)–(6.14), we infer that there exists $(P,{\tilde{P}},Z)$ with

$$\begin{aligned}&P\in L^2_w(\Omega ; L^\infty (0,T; V_1))\cap L^2_{\mathscr {P}}(\Omega ; L^2(0,T; V_3))\,,\\&{\tilde{P}}={\mathcal {L}}P \in L^2_w(\Omega ; L^\infty (0,T; V_1^*))\cap L^2_{\mathscr {P}}(\Omega ; L^2(0,T; V_1))\,,\\&Z \in L^2_{\mathscr {P}}(\Omega ; L^2(0,T; {\mathscr {L}}^2(K,V_1)))\,, \end{aligned}$$

such that as $\lambda \searrow 0$, possibly on a subsequence,

6.15 $$\begin{aligned} P_\lambda {\mathop {\rightharpoonup }\limits ^{*}}P \qquad&\text {in } L^2_w(\Omega ; L^\infty (0,T; V_1))\cap L^2_{\mathscr {P}}(\Omega ; L^2(0,T; V_3))\,, \end{aligned}$$

6.16 $$\begin{aligned} {\tilde{P}}_\lambda {\mathop {\rightharpoonup }\limits ^{*}}{\tilde{P}}\qquad&\text {in } L^2_w(\Omega ; L^\infty (0,T; V_1^*))\cap L^2_{\mathscr {P}}(\Omega ; L^2(0,T; V_1))\,, \end{aligned}$$

6.17 $$\begin{aligned} Z_\lambda \rightharpoonup Z \qquad&\text {in } L^2_{\mathscr {P}}(\Omega ; L^2(0,T; {\mathscr {L}}^2(K,V_1))). \end{aligned}$$

Now, thanks to **C1** and the regularity of $\varphi$, we have $\Psi ''(\varphi ) \in L^3(\Omega ; L^\infty (0,T; L^3({\mathcal {O}})))$, so in particular

$$\begin{aligned} \Psi ''_\lambda (\varphi ) \rightarrow \Psi ''(\varphi ) \qquad \text {in } L^3(\Omega \times Q)\,, \end{aligned}$$

and also, thanks to (6.16),

$$\begin{aligned} \Psi ''_\lambda (\varphi ){\tilde{P}}_\lambda \rightharpoonup \Psi ''(\varphi ){\tilde{P}} \qquad \text {in } L^{6/5}_{\mathscr {P}}(\Omega ; L^{6/5}(0,T; L^{6/5}({\mathcal {O}}))). \end{aligned}$$

Similarly, since ${{\mathbf {u}}}_\lambda \rightarrow {{\mathbf {u}}}$ in $L^q_{\mathscr {P}}(\Omega ;L^p(0,T; U))$ for every $q\ge 1$, from (6.15) we have

$$\begin{aligned} {{\mathbf {u}}}_\lambda \cdot \nabla P_\lambda \rightharpoonup {{\mathbf {u}}}\cdot \nabla P \qquad \text {in } L^{\ell }_{\mathscr {P}}(\Omega ; L^{\frac{2p}{p+2}}(0,T;H)) \qquad \forall \,\ell \in [1,2). \end{aligned}$$

Lastly, convergence (6.17) readily implies that

$$\begin{aligned} DB(\varphi )^*Z_\lambda \rightharpoonup DB(\varphi )^*Z \qquad \text {in } L^2_{\mathscr {P}}(\Omega ; L^2(0,T; H))\,, \end{aligned}$$

while by the linearity and continuity of the stochastic integral we have

$$\begin{aligned} \int _\cdot ^TZ_\lambda (s)\,{\mathrm d}W(s) \rightharpoonup \int _\cdot ^TZ(s)\,{\mathrm d}W(s) \qquad \text {in } L^2_{\mathscr {P}}(\Omega ; C^0([0,T]; V_1)). \end{aligned}$$

Consequently, we can let $\lambda \searrow 0$ in the variational formulation of the approximated system (6.1)–(6.4) and deduce that $(P,{\tilde{P}}, Z)$ solve the limit adjoint problem (2.10)–(2.13). The pathwise continuity of *P*, hence by comparison also of ${\tilde{P}}$, follows by classical methods using Itô’s formula on the limit equation.

### Uniqueness

By linearity of the adjoint system, it is enough to show that if $(P,{\tilde{P}}, Z)$ is a solution of (2.10)–(2.13) with $\alpha _1=\alpha _2=0$, then $\nabla P=0$, ${\tilde{P}}=0$, and $\nabla Z=0$. To this end, Itô’s formula for $\frac{1}{2}\left\| \nabla P\right\| _H^2$ yields

$$\begin{aligned}&\frac{1}{2}\left\| \nabla P(t)\right\| _{H}^2 +\int _{Q_t^T}|\nabla {\tilde{P}}|^2 +\frac{1}{2}\int _t^T\left\| \nabla Z(s)\right\| ^2_{{\mathscr {L}}^2(K,H)}\,{\mathrm d}s\\&\quad = -\int _{Q_t^T}\Psi ''(\varphi )|{\tilde{P}}|^2 +\int _{Q_t^T}{\tilde{P}} {{\mathbf {u}}}\cdot \nabla P +\int _{Q_t^T}(DB(\varphi )^*Z){\tilde{P}}\\&\qquad -\int _t^T\left( {\tilde{P}}(s), Z(s)\,{\mathrm d}W(s)\right) _H \end{aligned}$$

Now, as the computations are similar to the ones of Sect. 6.3, we avoid details for brevity. The terms on the right-hand side can be treated using **A1**, the Hölder–Young inequalities, the embedding $V_1\hookrightarrow L^6({\mathcal {O}})$, and the compactness inequality (2.1) as

$$\begin{aligned}&-\int _{Q_t^T}\Psi ''(\varphi )|{\tilde{P}}|^2 +\int _{Q_t^T}{\tilde{P}} {{\mathbf {u}}}\cdot \nabla P\\&\quad \le \varepsilon \int _{Q_t^T}|\nabla {\tilde{P}}|^2 +c_\varepsilon \int _0^t\left( 1+\left\| {{\mathbf {u}}}(s)\right\| _U^2\right) \left\| \nabla P(s)\right\| _H^2\,{\mathrm d}s\,, \end{aligned}$$

and similarly, since $DB(\varphi ){\tilde{P}}$ is ${\mathscr {L}}^2(K,H_0)$-valued by **A3**, by the Poincaré–Wirtinger inequality and **C2** we have

$$\begin{aligned} \int _{Q_t^T}(DB(\varphi )^*Z){\tilde{P}}&= \int _0^t(Z(s), DB(\varphi (s)){\tilde{P}}(s))_{{\mathscr {L}}^2(K,H)}\,{\mathrm d}s\\&\le \frac{1}{4}\int _t^T\left\| \nabla Z(s)\right\| ^2_{{\mathscr {L}}^2(K,H)}\,{\mathrm d}s +\varepsilon \int _{Q_t^T}|\nabla {\tilde{P}}|^2 \\&\qquad + c_\varepsilon \int _t^T\left\| \nabla P(s)\right\| _H^2\,{\mathrm d}s. \end{aligned}$$

Rearranging the terms and taking conditional expectations with respect to ${\mathscr {F}}_t$, we get that

$$\begin{aligned}&\left\| \nabla P(t)\right\| _{H}^2 +{{\mathbb {E}}}\left[ \int _{Q_t^T}|\nabla {\tilde{P}}|^2 +\int _t^T\left\| \nabla Z(s)\right\| ^2_{{\mathscr {L}}^2(K,H)}\,{\mathrm d}s\,\Bigg |{\mathscr {F}}_t\right] \\&\quad \le c{{\mathbb {E}}}\left[ \int _t^T\left\| \nabla P(s)\right\| _H^2\,{\mathrm d}s\,\Bigg |{\mathscr {F}}_t\right] \,, \end{aligned}$$

so that applying the backward stochastic Gronwall Lemma 6.1 and then taking expectations yield $\nabla {\tilde{P}}=0$ almost everywhere in $\Omega \times Q$, hence also ${\tilde{P}}=0$ almost everywhere in $\Omega \times Q$ since ${\tilde{P}}_{\mathcal {O}}=0$. Consequently, the stochastic integral appearing in the estimate above vanishes, and we deduce also $\nabla P=0$ in $L^2_{\mathscr {P}}(\Omega ; C^0([0,T]; H^d))$, from which ${\tilde{P}}=0$ in $L^2_{\mathscr {P}}(\Omega ; C^0([0,T]; V_1^*))$. Also, $\nabla Z=0$ in $L^2_{\mathscr {P}}(\Omega ; L^2(0,T; {\mathscr {L}}^2(K,H^d)))$. This concludes the proof of Theorem 2.6.

## Necessary Conditions for Optimality

In this last section, we prove the two versions of necessary conditions for optimality contained in Theorems 2.7–2.8. Let then ${{\mathbf {u}}}\in {\mathcal {U}}_{ad}$ be an optimal control for problem **(CP)** and let us set $(\varphi ,\mu ):=S({{\mathbf {u}}})$ as its corresponding optimal state. Let us also fix an arbitrary ${{\mathbf {v}}}\in {\mathcal {U}}_{ad}$.

By convexity of ${\mathcal {U}}_{ad}$ we have ${{\mathbf {u}}}+\delta ({{\mathbf {v}}}-{{\mathbf {u}}})\in {\mathcal {U}}_{ad}$ for all $\delta \in [0,1]$. Hence, setting $(\varphi _\delta , \mu _\delta ):=S({{\mathbf {u}}}+\delta ({{\mathbf {v}}}-{{\mathbf {u}}}))$, for every $\delta \in [0,1]$ the minimality of ${{\mathbf {u}}}$ yields

$$\begin{aligned} J(\varphi ,{{\mathbf {u}}})\le \frac{\alpha _1}{2}{{\mathbb {E}}}\int _Q|\varphi _\delta - \varphi _Q|^2 +\frac{\alpha _2}{2}{{\mathbb {E}}}\int _{\mathcal {O}}|\varphi _\delta (T)-\varphi _T|^2 +\frac{\alpha _3}{2}{{\mathbb {E}}}\int _Q|{{\mathbf {u}}}+\delta ({{\mathbf {v}}}-{{\mathbf {u}}})|^2\,, \end{aligned}$$

which entails in turn

$$\begin{aligned}&\frac{\alpha _1}{2}{{\mathbb {E}}}\int _Q\left( |\varphi _\delta |^2 - |\varphi |^2 - 2(\varphi _\delta -\varphi )\varphi _Q\right) \\&\quad +\frac{\alpha _2}{2}{{\mathbb {E}}}\int _{\mathcal {O}}\left( |\varphi _\delta (T)|^2 - |\varphi (T)|^2 - 2(\varphi _\delta -\varphi )(T)\varphi _T\right) \\&\quad +\frac{\alpha _3}{2}{{\mathbb {E}}}\int _Q\left( \delta ^2|{{\mathbf {v}}}-{{\mathbf {u}}}|^2 + 2\delta {{\mathbf {u}}}\cdot ({{\mathbf {v}}}-{{\mathbf {u}}})\right) \ge 0. \end{aligned}$$

Now, the functions $\zeta \mapsto {{\mathbb {E}}}\int _Q|\zeta |^2$ and $\zeta \mapsto {{\mathbb {E}}}\int _{\mathcal {O}}|\zeta |^2$ are Fréchet-differentiable on $L^2_{\mathscr {P}}(\Omega ; L^2(0,T; H))$ and $L^2(\Omega , {\mathscr {F}}_T; H)$, respectively. Hence, the mean-value theorem yields

$$\begin{aligned}&\alpha _1{{\mathbb {E}}}\int _Q\frac{\varphi _\delta -\varphi }{\delta }\int _0^1 \left( (\varphi + \tau (\varphi _\delta -\varphi ))-\varphi _Q\right) \,{\mathrm d}\tau \\&\quad +\alpha _3{{\mathbb {E}}}\int _Q{{\mathbf {u}}}\cdot ({{\mathbf {v}}}-{{\mathbf {u}}}) + \frac{\alpha _3}{2}\delta {{\mathbb {E}}}\left\| {{\mathbf {v}}}-{{\mathbf {u}}}\right\| ^2_{L^2(0,T; H^d)}\\&\quad +\alpha _2{{\mathbb {E}}}\int _{\mathcal {O}}\frac{\varphi _\delta -\varphi }{\delta }(T)\int _0^1 \left( (\varphi (T) + \tau (\varphi _\delta -\varphi )(T))-\varphi _T\right) \,{\mathrm d}\tau \ge 0. \end{aligned}$$

At this point, as $\delta \rightarrow 0$, we have ${{\mathbf {u}}}+\delta {{\mathbf {v}}}\rightarrow {{\mathbf {u}}}$ in ${\mathcal {U}}$, so (2.3)–(2.4) imply that

$$\begin{aligned} \int _0^1\left( (\varphi + \tau (\varphi _\delta -\varphi ))-\varphi _Q\right) \,{\mathrm d}\tau \rightarrow \varphi - x_Q \qquad&\text {in } L^p_{\mathscr {P}}(\Omega ; L^2(0,T; V_1))\,,\\ \int _0^1\left( (\varphi (T) + \tau (\varphi _\delta -\varphi )(T))-\varphi _T\right) \,{\mathrm d}\tau \rightarrow \varphi (T) - \varphi _T \qquad&\text {in } L^{p/3}(\Omega , {\mathscr {F}}_T; H). \end{aligned}$$

Moreover, Theorem 2.5 ensures that

$$\begin{aligned} \frac{\varphi _\delta -\varphi }{\delta } \rightharpoonup \theta _{{{\mathbf {v}}}-{{\mathbf {u}}}} \qquad&\text {in } L^p_{\mathscr {P}}(\Omega ; L^2(0,T; H))\,,\\ \frac{\varphi _\delta -\varphi }{\delta }(T) \rightharpoonup \theta _{{{\mathbf {v}}}-{{\mathbf {u}}}}(T) \qquad&\text {in } L^{p/3}(\Omega , {\mathscr {F}}_T; H). \end{aligned}$$

Hence, noting that $\frac{p}{3}\ge 2$, letting $\delta \rightarrow 0$ we obtain exactly (2.14), and Theorem 2.7 is proved.

Lastly, we note that (2.15) follows directly from (2.14) provided to show the duality relation

$$\begin{aligned} \alpha _1{{\mathbb {E}}}\int _Q\theta _{{{\mathbf {v}}}-{{\mathbf {u}}}}(\varphi -\varphi _Q)+ \alpha _2{{\mathbb {E}}}\int _{\mathcal {O}}\theta _{{{\mathbf {v}}}-{{\mathbf {u}}}}(T)(\varphi (T)-\varphi _T)= {{\mathbb {E}}}\int _Q\varphi ({{\mathbf {v}}}-{{\mathbf {u}}})\cdot \nabla P. \end{aligned}$$

In order to prove this, we can take $g=0$ and ${{\mathbf {h}}}={{\mathbf {v}}}-{{\mathbf {u}}}$ in the duality relation (6.9), and then let $\lambda \searrow 0$ thanks to the convergences (5.9)–(5.10). This concludes the proof of Theorem 2.8.
